# The Arabidopsis *APOLO* and human *UPAT* sequence-unrelated long noncoding RNAs can modulate DNA and histone methylation machineries in plants

**DOI:** 10.1186/s13059-022-02750-7

**Published:** 2022-08-29

**Authors:** Camille Fonouni-Farde, Aurélie Christ, Thomas Blein, María Florencia Legascue, Lucía Ferrero, Michaël Moison, Leandro Lucero, Juan Sebastián Ramírez-Prado, David Latrasse, Daniel Gonzalez, Moussa Benhamed, Leandro Quadrana, Martin Crespi, Federico Ariel

**Affiliations:** 1 Instituto de Agrobiotecnología del Litoral, CONICET, Universidad Nacional del Litoral, Colectora Ruta Nacional 168 km 0, 3000 Santa Fe, Argentina; 2 Institute of Plant Sciences Paris Saclay IPS2, CNRS, INRA, Université Evry, Université Paris-Saclay, Bâtiment 630, 91405 Orsay, France; 3 Institute of Plant Sciences Paris-Saclay IPS2, Université de Paris, Bâtiment 630, 91405 Orsay, France; 4 Institut de Biologie de l’Ecole Normale Supérieure (IBENS), Centre National de la Recherche Scientifique (CNRS), Institut National de la Santé et de la Recherche Médicale (INSERM), Ecole Normale Supérieure, PSL Research University, 75005 Paris, France

**Keywords:** Long noncoding RNA, DNA methylation, PRC1, Polycomb, RdDM, Thermomorphogenesis, Auxin, R-loop, *APOLO*, LHP1, *UPAT*, VIM1, UHRF1, *YUCCA2*

## Abstract

**Background:**

RNA-DNA hybrid (R-loop)-associated long noncoding RNAs (lncRNAs), including the Arabidopsis lncRNA *AUXIN-REGULATED PROMOTER LOOP* (*APOLO*), are emerging as important regulators of three-dimensional chromatin conformation and gene transcriptional activity.

**Results:**

Here, we show that in addition to the PRC1-component LIKE HETEROCHROMATIN PROTEIN 1 (LHP1), *APOLO* interacts with the methylcytosine-binding protein VARIANT IN METHYLATION 1 (VIM1), a conserved homolog of the mammalian DNA methylation regulator UBIQUITIN-LIKE CONTAINING PHD AND RING FINGER DOMAINS 1 (UHRF1). The *APOLO*-VIM1-LHP1 complex directly regulates the transcription of the auxin biosynthesis gene *YUCCA2* by dynamically determining DNA methylation and H3K27me3 deposition over its promoter during the plant thermomorphogenic response. Strikingly, we demonstrate that the lncRNA *UHRF1 Protein Associated Transcript* (*UPAT*), a direct interactor of UHRF1 in humans, can be recognized by VIM1 and LHP1 in plant cells, despite the lack of sequence homology between *UPAT* and *APOLO*. In addition, we show that increased levels of *APOLO* or *UPAT* hamper VIM1 and LHP1 binding to *YUCCA2* promoter and globally alter the Arabidopsis transcriptome in a similar manner.

**Conclusions:**

Collectively, our results uncover a new mechanism in which a plant lncRNA coordinates Polycomb action and DNA methylation through the interaction with VIM1, and indicates that evolutionary unrelated lncRNAs with potentially conserved structures may exert similar functions by interacting with homolog partners.

**Supplementary Information:**

The online version contains supplementary material available at 10.1186/s13059-022-02750-7.

## Background

In eukaryotes, chromatin structure, composition, and dynamics determine the three-dimensional configuration of the genome and are critical for gene regulation, cell fate, and function [1, 2]. Chromatin conformation and related transcriptional states depend on coordinated shifts in DNA methylation, post-translational modifications of histone tails, and RNA interference (RNAi) pathways [3–5]. In mammalian genomes, DNA methylation is primarily observed at CpG dinucleotides and is estimated to occur at ~70–80% of CpG sites throughout the genome [6]. A quarter of non-CG methylation is found in embryonic stem cells, while the remaining unmethylated CpG sites are mostly found in dense clusters, near gene promoters, referred to as CpG islands [7–9]. Unlike in mammals, DNA methylation in plants predominantly occurs on transposons and other repetitive DNA elements, and exists in all possible sequence contexts: symmetric CpG and CpHpG—where H is any base except G—or asymmetric CpHpH [10, 11]. Non-CpG methylation is mainly distributed at heterochromatin regions, nevertheless, some euchromatic genes exhibit cytosine methylation in their promoter, a feature likely correlated with tissue specificity [10].

In *Arabidopsis thaliana*, the establishment of de novo methylation in all sequence contexts is catalyzed by DOMAINS REARRANGED METHYLTRANSFERASE 2 (DRM2), a plant homolog of mammalian DNA (CYTOSINE-5)-METHYLTRANSFERASE 3 (DNMT3) a and b [12, 13]. DRM2 is guided to chromatin by 24-nucleotide small interfering RNAs (24nt siRNAs) as part of a pathway known as RNA-directed DNA methylation (RdDM; [14]). RdDM involves two non-redundant plant specific RNA polymerases, Pol IV and Pol V, in addition to the canonical RNA interference machinery, which requires the activity of RNA-dependent RNA polymerase 2 (RDR2) and members of the Dicer and Argonaute families [15–18]. Once established, DNA methylation is maintained by three different pathways depending on the sequence context. CpG methylation depends on DNA METHYLTRANSFERASE 1 (MET1), a homolog of mammalian DNA METHYLTRANSFERASE 1 (DNMT1; [19, 20]). CpHpG methylation relies on CHROMOMETHYLASE 3 (CMT3), a plant specific DNA methyltransferase that associates with SU(VAR)3–9 HOMOLOG (SUVH) histone methyltransferases and recognizes dimethylated histone 3 tails at lysine 9 (H3K9me2; [11, 21–24]). Finally, CpHpH methylation is maintained through persistent de novo methylation by DRM2 and by the CMT3 homolog CHROMOMETHYLASE 2 (CMT2) that specifically reads the H3K9me2 mark [24–26].

Beside the contribution of methyltransferases, the DNA methylation process involves methylcytosine-binding proteins of the VARIANT IN METHYLATION (VIM/ORTH) family [27, 28]. VIM/ORTH proteins are homologous to the mammalian UBIQUITIN-LIKE CONTAINING PHD AND RING FINGER DOMAINS (UHRF) proteins known to regulate cytosine methylation through the recruitment of DNMT1 to target loci [29–32]. In particular, UHRF1 functions as an epigenetic regulator maintaining DNA methylation and histone modifications [33] and is stabilized by direct interaction with the long noncoding RNA (lncRNA) *UPAT* (*UHRF1 Protein Associated Transcript*; [34]). VIM proteins are characterized by the presence of a PHD domain recognizing trimethylated histone 3 tails at lysine 4 (H3K4me3; [35–38]), a SRA (SET [Su(var), Enhancer of Zeste, Trithorax], and RING [Really Interesting New Gene] Associated) domain that can associate with methylated DNA [11, 27, 39], and two RING domains conferring ubiquitin E3 ligase activity [31]. The Arabidopsis genome encodes five highly similar VIM proteins—named VIM1 to 5—and a related protein ORTH-LIKE1 (ORL1/VIM6; [31]). VIM1, VIM2, and VIM3 maintain MET1-mediated cytosine methylation at CpG dinucleotides throughout the genome and have been reported to function entirely redundantly to mediate epigenetic transcriptional silencing in collaboration with MET1 [25, 28, 40].

Interestingly, aberrant changes in active and repressive histone modifications have been observed in *vim1/2/3* and *met1* mutants, supporting that VIM proteins coordinate DNA methylation and histone modification [41–44]. In Arabidopsis, the transcriptionally repressive mark histone H3 lysine 27 trimethylation (H3K27me3) is largely restricted to the transcribed regions of single genes, exhibiting a global anti-correlated distribution with centromeric-enriched DNA methylation [11, 43, 45]. However, a loss of H3K27me3 was also reported at gene bodies in *met1* mutants and at specific VIM1 targets located in euchromatic regions in the *vim1/2/3* triple mutant [43]. Notably, the correlation between DNA hypomethylation and H3K27me3 reduction in the *vim1/2/3* mutation was more prevalent in promoter regions than in transcribed regions [44]. Collectively, these observations hint at non-canonical mechanisms linking DNA methylation and repressive histone modifications over specific transcriptionally active loci.

In *A. thaliana*, H3K27me3 is deposited by Polycomb group (PcG) proteins in euchromatic regions containing protein-coding genes and is maintained by LIKE HETEROCHROMATIN PROTEIN 1 (LHP1), a component of the plant Polycomb Repressive Complex 1 (PRC1) and a homolog of mammalian HETEROCHROMATIN PROTEIN 1 (HP1; [46–49]). LHP1 capacity to localize and mediate epigenetic repression of PcG target genes was shown to rely on its RNA-binding hinge region, suggesting that LHP1 activity could be modulated by interacting RNAs [50]. Consistently, it was shown that the lncRNA *APOLO* (*AUXIN-REGULATED PROMOTER LOOP*) can regulate local chromatin conformation by decoying LHP1 away from target loci. *APOLO* directly recognizes multiple distant and independent auxin-related loci across the *A. thaliana* genome by short sequence complementarity and the formation of DNA-RNA duplexes (R-loops) [51, 52].

Here, we demonstrated that in addition to the PRC1 component LHP1, *APOLO* lncRNA interacts in vivo with VIM1, the plant homolog of UHRF1, linking Polycomb and DNA methylation machineries. RNA sequencing analyses of *APOLO* over-expression and *vim1* mutant lines revealed that *APOLO* and VIM1 control a large common set of genes related to the thermomorphogenic response. In particular, the *APOLO*-VIM1-LHP1 complex directly targets the heat-responsive auxin-biosynthetic gene *YUCCA2* and conjointly mediates cytosine methylation and H3K27me3 deposition at its promoter, representing a new epigenetic mechanism regulating the plant response to warm temperatures. Strikingly, we also demonstrate that the lncRNA *UPAT*, known to recognize UHRF1 in humans, can interact with VIM1 and LHP1 in plant cells despite the lack of sequence homology between *UPAT* and *APOLO*. Furthermore, the over-expression of *APOLO* or *UPAT* trigger a similar transcriptional reprogramming in Arabidopsis, and *UPAT* constitutive transcription in plants precludes LHP1 and VIM1 binding to the *YUCCA2* promoter region. Hence, sequence-unrelated lncRNAs with potentially similar structures may exert similar molecular functions across kingdoms, integrating the epigenetic regulation of gene expression.

## Results

### Long noncoding RNA *APOLO* associates with the methylcytosine-binding protein VIM1 in vivo

**Fig. 1:**
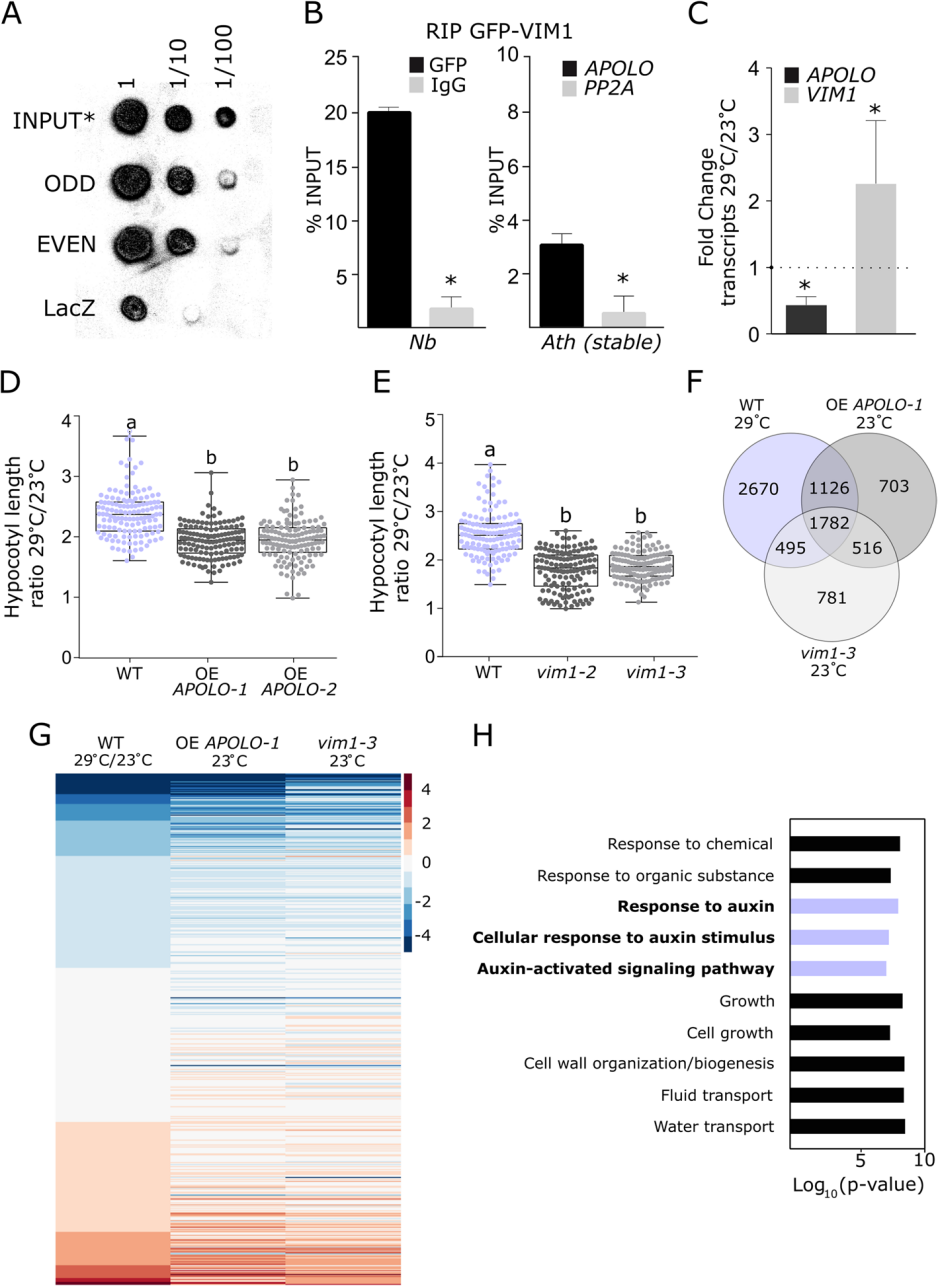
The lncRNA *APOLO* and the methylcytosine-binding protein VIM1 are thermomorphogenesis regulators. **A** Chromatin isolation by RNA purification (ChIRP)-Dot blot analysis of *APOLO* interaction with GFP-VIM1. ChIRP was performed using ODD and EVEN sets of probes against *APOLO* or using LacZ probes as a negative control. Dot blots are revealed with an anti-GFP antibody and an HRP-conjugated secondary antibody. Diluted INPUT (*1/50) were used as loading control. **B** RNA immunoprecipitation (RIP) assay in *Nicotiana benthamiana* leaves transiently co-transformed with *APOLO* and *GFP-VIM1* translational fusion expressed under the control of the 35S-CaMV promoter, or in *Arabidopsis thaliana* 2-week-old *VIM1* over-expression (OE *VIM1-1*) seedlings. Results are expressed as a percentage of the INPUT fraction. Anti-IgG antibodies were used as a negative control, and the asterisks indicate Student’s *t* test ≤ 0.05 (*n* = 3) between anti-GFP and anti-IgG RIPs. The non-specific background level of RNA precipitation (*PP2A*) is also shown in Arabidopsis. **C** Fold change of *APOLO* and *VIM1* expression levels in relation to 23 °C control conditions in 4-day-old wild-type (WT) seedlings treated with heat (29 °C) for 6 h. Asterisks indicate Student’s *t* test ≤ 0.05 (*n* = 3) for levels of each corresponding gene between 23 and 29 °C. **D**, **E** Boxplots showing hypocotyl length quantification ratio at 29 °C over 23 °C of 4-day-old OE *APOLO-1*, OE *APOLO-2* (**D**) or *vim1-2*, *vim1-3* (**E**) seedlings and their associated WT. Values are represented by colored points. **F** Venn diagram of differentially expressed transcripts in WT treated with heat (29 °C) for 6 h, and in untreated OE *APOLO-1* and *vim1-3* seedlings. **G** Heatmap showing log_2_(fold change) compared to WT 23 °C. A correlation of up- and downregulated genes in WT in response to 29 °C is observed for OE *APOLO-1* and *vim1-3* at 23 °C. **H** Gene Ontology (GO) enrichment analysis of upregulated transcripts in WT treated with heat. Top ten GO categories with more significant *p*-values are shown. Violet bars: auxin-related pathways. The ShinyGO Browser is located at bioinformatics.sdstate.edu and published in [54]. In **A**, each sample was serially diluted as indicated in the plot, and two additional biological replicates are shown in Additional file 1: Fig. S1A. In **B**, bars represent average ± SD (*n* = 3 biological replicates). In **C**, transcript levels are normalized relatively to the untreated control to show fold changes. Bars represent average ± SD (*n* = 3 biological replicates). In **D**, **E**, results are the mean of three biological replicates and letters indicate significant differences compared to WT, based on a Kruskal-Wallis test (*α* = 0.05; *n* ≥ 134)

In order to investigate the composition of the ribonucleoprotein complexes integrated by the lncRNA *APOLO*, we performed an exploratory Chromatin isolation by RNA purification (ChIRP) followed by protein precipitation and mass spectrometry. We used two independent sets of biotinylated DNA probes against *APOLO* (ODD and EVEN sets) and LacZ probes as a negative control [53]. Among the proteins identified by at least two positive hits of unique peptides in ODD and EVEN, excluded from LacZ ChIRPs, we found the protein VARIANT IN METHYLATION 1 (VIM1, AT1G57820). This interaction was confirmed by *APOLO*-ChIRP-dot blot in stably transformed *Arabidopsis thaliana* seedlings over-expressing *GFP-VIM1* (OE *VIM1-1*) (Fig. 1A and Additional file 1: Fig. S1A). Transient expression of *GFP*-*VIM1* in *Nicotiana benthamiana* and *A. thaliana* leaves revealed that VIM1 is located in the cell nucleus, in agreement with previous observations [27]. Moreover, this localization was observed regardless of the co-expression with nuclear-enriched *APOLO* transcripts (Additional file 1: Fig. S1B-C). Anti-GFP RNA immunoprecipitation (RIP) in *N. benthamiana* leaves transiently co-transformed with *GFP*-*VIM1* and *APOLO*, or in Arabidopsis OE *VIM1-1* (Fig. 1B; *VIM1* levels shown in Additional file 1: Fig. S2A), revealed a high enrichment of *APOLO* transcripts in VIM1 immunoprecipitated samples. These results indicate that VIM1 is part of a novel ribonucleoprotein complex integrated by *APOLO* lncRNA.

### *APOLO* and VIM1 are important thermomorphogenesis regulators in *Arabidopsis thaliana*

Abnormal morphological phenotypes, including DNA methylation-dependent late flowering, were previously reported for the *vim1/2/3* triple mutant in contrast to the *vim1* single mutant exhibiting no evident phenotype [28]. We thus wondered in what developmental context the *APOLO*-VIM1 interaction may occur and exert a regulatory role over target genes. By exploring the eFP Arabidopsis transcriptomic database [55], we found that *VIM1*, *VIM2*, and *VIM3* genes showed different transcriptional dynamics following heat stress (38 °C followed by recovery at 25 °C; Additional file 1: Fig. S3A, blue box). Therefore, we evaluated the expression of these three genes and *APOLO* at 29 °C, a warm temperature usually faced by Arabidopsis in the wild [56]. After 6 h of exposure to 29 °C, transcript levels of *APOLO* decreased while those of *VIM1*, as well as the warmth-response markers *PIF4* and *YUC8*, increased (Fig. 1C; Additional file 1: Fig. S3B-C). Unlike *VIM1*, expression of *VIM2* and *VIM3* were repressed or unaffected, respectively (Additional file 1: Fig. S3C). Interestingly, anti-GFP RIP in *N. benthamiana* leaves transiently co-transformed with *GFP*-*VIM2* and *APOLO* revealed that *APOLO* can also interact in vivo with VIM2 (although showing a weaker interaction than VIM1; Additional file 1: Fig. S3D for VIM2 nuclear localization and Additional file 1: Fig. S3E for RIPs), suggesting that this complex may also form during Arabidopsis development or in response to the environment.

To assess whether the ribonucleoprotein complex integrated by *APOLO* and *VIM1* is involved in the response to warmth, we tested the effect of their deregulation on the thermomorphogenesis response. Two independent *APOLO* over-expression (OE) lines (OE *APOLO-*1 and OE *APOLO-*2; [52]), together with two independent *vim1* insertional mutants resulting in a knockdown of *VIM1* (*vim1-2* and *vim1-3*, respectively; Additional file 1: Fig. S2B-C), exhibited an impaired hypocotyl elongation after 4 days at 29 °C (Fig. 1D, E; Additional file 1: Fig. S4A-B; Additional file 2: Table S1). *vim1-3* mutant plants transformed with *proVIM1:GFP:VIM1* exhibited a partially rescued hypocotyl elongation phenotype (Additional file 1: Fig. S4C-D; Additional file 2: Table S1). Additionally, we observed a slight but significant reduction of hypocotyl elongation at 29 °C in one out of two independent RNAi *APOLO* lines (RNAi *APOLO-*1 and RNAi *APOLO-*2; [51]) as well as in the knockout line obtained by CRISPR-Cas9-mediated deletion of the full *APOLO* locus (*CRISPR*-APOLO; Additional file 1: Fig. S2D, S4E-H; Additional file 2: Table S1), and a minor but significant induction of hypocotyl elongation in OE *VIM1* lines (OE *VIM1-*1 and OE *VIM1-*2; Additional file 1: Fig. S2A, Fig. S4F, S4I; Additional file 2: Table S1). Altogether, these results suggest that *APOLO* and VIM1 regulate thermomorphogenesis and incidentally indicate that *VIM1* is not redundant with *VIM2* or *VIM3* in this developmental context.

To pinpoint molecular mechanisms that could explain impaired thermomorphogenesis upon *APOLO* or *VIM1* deregulation, we profiled the transcriptomes of 4-day-old OE *APOLO-*1 and *vim1-3* seedlings grown at 23 °C with RNA-Seq of 4-day-old wild-type (WT) seedlings grown at 23 °C and subjected or not to heat (29 °C) for 6 h. In WT seedlings, warmth caused the downregulation of over 3400 transcripts and the upregulation of approximately 2650 transcripts (Additional file 2: Table S2). Remarkably, 56% of the total set of up- and downregulated transcripts at 29 °C in WT were already deregulated at 23 °C in OE *APOLO*-1 and/or *vim1-3* (3403 genes over 6073; Fig. 1F), whereas 52% of these transcripts (1782 out of 3403) were deregulated in both OE *APOLO*-1 and *vim1-3* at 23 °C (Fig. 1F, central intersection). Moreover, a subset of up- and downregulated genes in response to warmth in WT showed similar expression profiles in OE *APOLO*-1 and *vim1-3* at ambient temperature (Fig. 1G). Upregulated genes in WT in response to warmth were enriched in “Response to auxin,” “Cellular response to auxin stimulus,” and “Auxin-activated signaling pathway” GO categories (Fig. 1H; Additional file 1: Fig. S5), consistent with the well-established role of auxin in thermomorphogenesis [57]. Altogether, these transcriptomic analyses hint at a critical role for the *APOLO*-VIM1 complex in the transcriptional reprogramming of gene expression in response to warm temperatures.

**Fig. 2:**
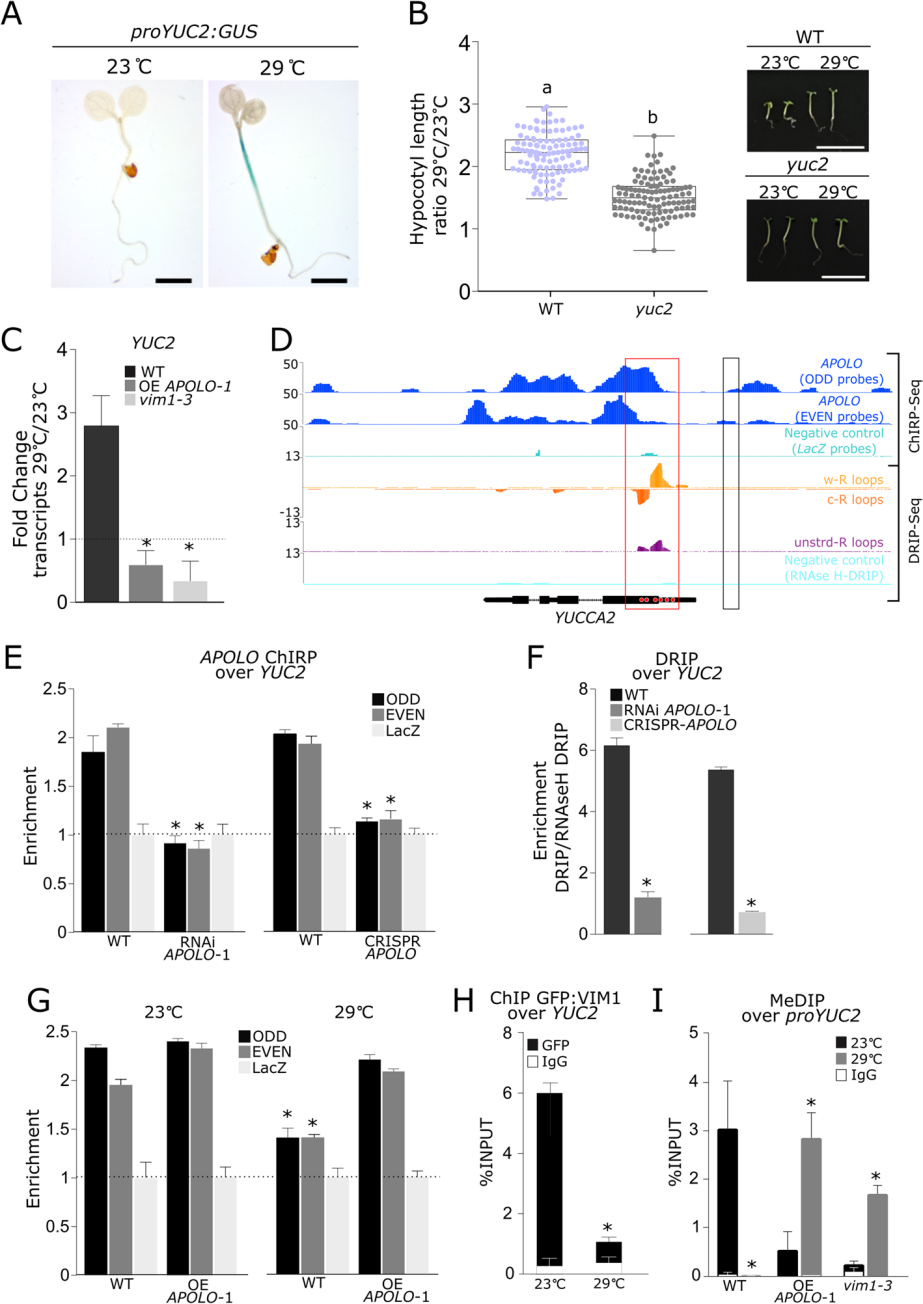
The thermomorphogenesis-related gene *YUCCA2* is directly co-regulated by *APOLO* and VIM1. **A** Histochemical localization of GUS activity in 4-day-old seedlings containing the pro*YUCCA2:GUS* construct, grown at 23 °C or 29 °C. Scale bars, 0.1 cm. **B** Boxplots showing hypocotyl length quantification ratio at 29 °C over 23 °C of 4-day-old *yuc2* seedlings and their associated wild-type (WT). Values are represented by colored points. Representative morphological phenotypes are shown on the right. Scale bars, 1 cm. **C***YUCCA2* (*YUC2*) transcript levels in 4-day-old WT, OE *APOLO-1*, and *vim1-3* seedlings treated or not with heat (29 °C) for 6 h. Asterisks indicate Student’s *t* test ≤ 0.05; *n* = 3 between each corresponding genotype and WT. **D** Epigenetic profile at the *YUC2* locus. Tracks 1 to 3 [52]: *APOLO* recognition by chromatin isolation by RNA purification (ChIRP) sequencing, using ODD (Track 1) and EVEN (Track 2) sets of probes against *APOLO*. ChIRP negative control using LacZ probes is shown in Track 3. Tracks 4 to 7 [61]: R-loop formation by DNA:RNA immunoprecipitation (DRIP) sequencing, on Watson (Track 4), Crick strand (Track 5), or unstranded sequencing (Track 6). DRIP negative control after RNAseH treatment is shown in Track 7. Gene annotation is shown at the bottom. On the *YUCCA2* schematic representation in the bottom, red dots indicate the presence of six GAAGAA/TTCTTC boxes which may mediate *APOLO* recognition according to Ariel et al. [52]. **E***APOLO* association to DNA of the *YUC2* locus by ChIRP-qPCR in WT, RNAi *APOLO-1*, and CRISPR-*APOLO* plants. The background level was determined using a set of probes against LacZ RNA. **F** RNA-DNA hybrid (R-loop) formation at the *YUC2* locus by DRIP-qPCR in WT and RNAi *APOLO-1* plants. **G** R-loop formation at the *YUC2* locus by DRIP-qPCR in WT and OE *APOLO-1* plants at 23 °C or 29 °C. Asterisks indicate a significant reduction (Student’s *t* test ≤ 0.05; *n* = 3) between R-loop levels at 23 °C or 29 °C in WT plants. **H** Chromatin immunoprecipitation (ChIP)-qPCR analysis of VIM1 binding at the *YUC2* promoter in 4-day-old *VIM1* over-expression (OE *VIM1-1*) seedlings treated or not with heat (29 °C) for 6 h. **I** Methylated DNA immunoprecipitation (MeDIP)-qPCR analysis at the *YUC2* promoter in 4-day-old *APOLO* over-expression (OE *APOLO-1*) or *vim1-3* mutant seedlings treated or not with heat (29 °C) for 6 h. In **A**, one representative picture out of ten stained seedlings is shown. In **B**, results are the mean of three biological replicates and letters indicate significant differences compared to WT, based on a Mann-Whitney test (*α* = 0.05; *n* ≥ 110). In **C**, transcript levels are normalized relatively to the untreated control to show fold changes. Bars represent average ± SD (*n* = 3 biological replicates). In **E**, **F**, bars represent average ± SD (*n* = 3 biological replicates). In **G**, **H**, results are expressed as a percentage of the INPUT fraction. Anti-IgG antibodies were used as a negative control. Bars represent SD (*n* = 3 biological replicates), except for **H** (*n* = 2 closest biological replicates out of 3 performed, all showing the same trend) and the asterisk indicates the Student’s *t* test ≤ 0.05, between 23 and 29 °C. In **E** and **G**, asterisks indicate the Student’s *t* test ≤ 0.05; *n* = 3, between WT and the corresponding genotype. In **I** asterisks indicate the Student’s *t* test ≤ 0.05; *n* = 3, between 23 and 29 °C for each genotype

We then aimed at identifying the potential direct targets of the *APOLO*-VIM1 complex during the thermomorphogenic response. Given the well-known role of VIM1 in DNA methylation, we compared the list of hypomethylated genes in *vim1 vs*. WT identified by bisulfite sequencing analyses (BiS-Seq; [25]), with potential *APOLO* direct targets identified by *APOLO*-ChIRP-Seq [52]. Significant enrichment of hypomethylated genes in the CpG and CpHpH contexts was observed among *APOLO* potential targets (Additional file 2: Table S3). Interestingly, among the eleven potential *APOLO* targets exhibiting a differential CpHpH methylation pattern in the *vim1* mutant, we found *YUCCA2* (*YUC2*, AT4G13260; Additional file 2: Table S3), a heat-responsive gene involved in auxin biosynthesis [58, 59]. By using a *proYUC2:GUS* transcriptional fusion [60], we observed that the *YUC2* promoter region is activated in the hypocotyl of 4-day-old seedlings grown at 29°C (Fig. 2A). In addition, *yuc2* loss-of-function mutant seedlings [60] exhibited a reduced hypocotyl elongation after 4 days at 29°C (Fig. 2B; Additional file 2: Table S1), indicating that *YUC2* is required for a proper thermomorphogenic response. Consistently, we observed that the transcriptional activation of *YUC2* after 6 h at 29 °C was impaired in OE *APOLO*-1 and *vim1-3* showing an altered hypocotyl elongation (Fig. 2C) and a generally altered transcriptional behavior in *APOLO*- and *VIM1*-deregulated lines (Additional file 1: Fig. S6A). Among the other six genes assessed together with *YUC2*, three were induced by warmth in WT, whereas all of them exhibited an altered expression upon deregulation of *APOLO* and/or *VIM1* (Additional file 1: Fig. S6A). Altogether, our results further suggest that the *APOLO*-VIM1 complex directly regulates a common subset of genes including *YUC2* to trigger the plant auxin-related thermomorphogenic response at warm temperatures.

### *APOLO* and VIM1 directly mediate heat-dependent methylation at the *YUCCA2* promoter

As *APOLO* recognizes its target loci by sequence complementarity and R-loop formation [52], we first investigated the *APOLO* binding profile over the *YUC2* locus by ChIRP-Seq. Compared to the LacZ probes used as a negative control, ODD and EVEN probes showed specific binding of *APOLO* across the *YUC2* locus, notably to the exon 1, displaying six *APOLO*-binding motifs “GAAGAA/TTCTTC” (Fig. 2D, first three tracks in blue and light blue, binding motifs indicated as red dots over the *YUC2* locus). *APOLO*-ChIRP-qPCR over *YUC2* gene body in WT, RNAi *APOLO-*1, and CRISPR-*APOLO* seedlings confirmed the specificity of the interaction (Fig. 2E). Furthermore, DNA-RNA immunoprecipitation followed by sequencing (DRIP-Seq; [61]) revealed that R-loop formation occurs in the exon 1 of *YUC2*, in correlation with *APOLO* binding (Fig. 2D, red box), suggesting that *APOLO* directly recognizes *YUC2* through the formation of R-loops. Consistently, DRIP-qPCR in WT, RNAi *APOLO-*1 and CRISPR-*APOLO* seedlings confirmed that R-loop formation over the *YUC2* locus depends on *APOLO* expression (Fig. 2F). Consistently, DRIP-qPCR revealed that R-loop formation over *YUC2* diminishes significantly more in WT than in OE *APOLO*-1 at 29°C, further supporting that *YUC2* R-loop at 23°C is mediated by *APOLO* (Fig. 2G).

Interestingly, a NRPE1 Pol V-subunit chromatin immunoprecipitation (ChIP)-Seq [62] indicated that the *YUC2* promoter region, located 560 to 1010 bp upstream of *YUC2* TSS, is directly regulated by Pol V in a MET1-dependent manner (Fig. 2D, black box indicating the region for results in Additional file 1: Fig. S6B). In addition, a small RNA-Seq profiling reported a temperature-dependent accumulation of RdDM-related 24nt siRNAs over this locus ( [63]; Additional file 1: Fig. S6C), suggesting that DNA methylation in this regulatory region may contribute to the temperature-induced transcriptional regulation of *YUC2*. We thus assessed VIM1 binding and DNA methylation at the *YUC2* promoter region in the same developmental stage as our phenotypic characterization. GFP-VIM1 ChIP performed in 4-day-old seedlings grown at 23 °C and treated or not at 29 °C for 6 h revealed that VIM1 binds to the region located 357 to 494 bp upstream of *YUC2* TSS and that this binding is reduced at 29 °C compared to 23 °C (Fig. 2H). Methylated DNA immunoprecipitation (MeDIP) demonstrated that DNA methylation is reduced in this region in the WT after 6 h at 29 °C (Fig. 2I). Furthermore, in OE *APOLO-*1 and *vim1-3*, DNA methylation levels resulted to be lower at 23 °C and increased at 29 °C, exhibiting the opposite behavior to the WT (Fig. 2I).

To further support the relevance of RdDM on *YUC2* regulation in the context of thermomorphogenesis, we additionally characterized the physiological response to warm temperatures of RdDM mutants *nrpd2a* (a common subunit of Pol IV and Pol V), *rdr2-5*, *dcl3-1* and *ago4-8*, as well as CpG methyltransferase mutant *met1*-*2* and non-CpG methyltransferase mutants *cmt2-7* and *cmt3-11* (for a visual summary of their respective roles in DNA methylation pathways, see Additional file 1: Fig. S7A). Hypocotyl elongation at 29 °C was reduced in *nrpd2a*, *met1-2*, *rdr2-5*, *dcl3-1*, and *ago4-8* backgrounds, whereas it was unaffected in *cmt3-11* and slightly enhanced in *cmt2-7* backgrounds (Additional file 1: S7B-J; Additional file 2: Table S1). In agreement, BiS-seq analyses [25] revealed that RdDM and *met1-2* mutants showing impaired thermomorphogenesis also displayed reduced CpHpH methylation levels in the *YUC2* regulatory region (Additional file 1: Fig. S6D-E, blue box), further supporting that epigenetic modifications regulating DNA methylation at *YUC2* promoter are critical to modulate thermomorphogenesis. Taken together, our results indicate that *APOLO* and VIM1 directly mediate DNA methylation at *YUC2* promoter in response to warm temperatures, in a process likely modulated by the RdDM pathway.

### VIM1 and LHP1 cooperate to regulate temperature-dependent histone and DNA methylation at the *YUCCA2* promoter

**Fig. 3:**
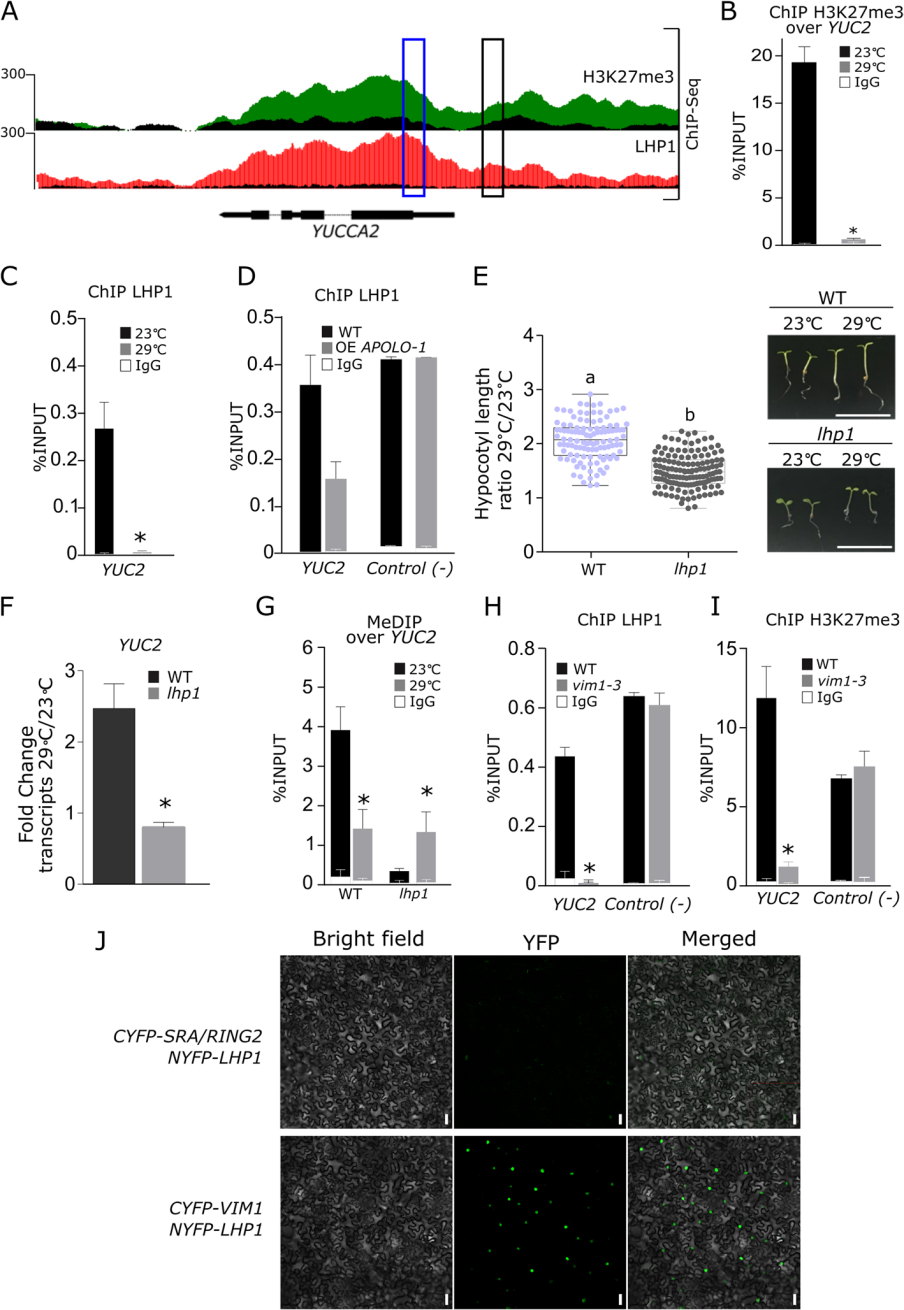
VIM1 and LHP1 co-regulate histone and DNA methylation at the *YUCCA2* promoter. **A** Epigenetic landscape at the *YUCCA2* (*YUC2*) locus. Track 1: H3K27me3 deposition by ChIP-sequencing. Track 2: LHP1 binding by ChIP-sequencing [49]. The black signal represents the input sequencing for each respective track. Gene annotation is shown in the bottom. **B** ChIP-qPCR analysis of H3K27me3 deposition at the *YUC2* promoter in 4-day-old wild-type (WT) seedlings treated or not with heat (29 °C) for 6 h. The asterisk indicates the Student’s *t* test ≤ 0.05, *n*=3, between 23 and 29 °C. **C** ChIP-qPCR analysis of LHP1 binding at the *YUC2* promoter in 4-day-old WT seedlings treated or not with heat (29 °C) for 6 h. **D** ChIP-qPCR analysis of LHP1 binding at the *YUC2* gene body in WT and *APOLO* over-expression (OE *APOLO-1*) plants. The negative control corresponds to an *APOLO*-independent LHP1 target gene AT4G23720 [49]. **E** Boxplots showing hypocotyl length quantification ratio at 29 °C over 23 °C of 4-day-old *lhp1* seedlings and their associated WT. Values are represented by colored points. Representative morphological phenotypes are shown on the right. Scale bars, 1 cm. **F***YUC2* transcript levels in 4-day-old WT and *lhp1* seedlings treated or not with heat (29 °C) for 6 h. **G** Methylated DNA immunoprecipitation (MeDIP)-qPCR analysis at the *YUC2* promoter in 4-day-old *lhp1* mutant seedlings treated or not with heat (29 °C) for 6 h. **H**, **I** Chromatin immunoprecipitation (ChIP)-qPCR analyses of LHP1 binding (in **H**) and H3K27me3 deposition (in **I**) at the *YUC2* promoter in 4-day-old WT and *vim1-3* mutant seedlings. The negative control corresponds to an *APOLO*-independent LHP1 target gene AT4G23720 [49]. **J** Bimolecular fluorescence complementation (BiFC) assay in transiently transformed *Nicotiana benthamiana* leaves. CYFP was fused to VIM1 or SRA/RING2, and NYFP was fused to LHP1. In both panels, bright-field images (left), YFP fluorescence (middle), and merged images (right) are shown. A zoom-in including mCherry constitutive expression for nuclei and membrane visualization is shown in Additional file 1: Fig. S8A. Scale bars, 50 μm. In **B**–**D** and **G**–**I**, results are expressed as a percentage of the INPUT fraction. Anti-IgG antibodies were used as a negative control. Bars represent standard deviation (*n* = 3 biological replicates). In **E**, results are the mean of three biological replicates and letters indicate significant differences compared to WT, based on a Mann–Whitney test (*α* = 0.05; *n* ≥ 111). In **F**, transcript levels are normalized relatively to the untreated control to show fold changes. Bars represent average ± SD (*n* = 3 biological replicates) except for **G** (*n* = 2 closest biological replicates out of 3 performed, all showing the same trend). In **J**, one representative picture out of six biological replicates is shown

Given that the plant PRC1 component LHP1 recognizes *APOLO* in vivo and co-regulates common target loci across the Arabidopsis genome [51, 52], we explored whether the *YUC2* locus was regulated by LHP1 and the related transcriptionally repressive mark H3K27me3. ChIP-Seq analyses [49] showed that the *YUC2* locus is enriched in LHP1 and H3K27me3 in standard growing conditions (Fig. 3A), whereas both marks are drastically reduced at 29 °C over the promoter region (Fig. 3A, black box; Fig. 3B, C). Moreover, LHP1 binding to the *YUC2* gene body was impaired by *APOLO* over-expression (Fig. 3A, blue box; Fig. 3D). Consistent with an involvement of LHP1 in the thermomorphogenic response, 4-day-old *lhp1* loss-of-function mutant seedlings [49] exhibited a reduced hypocotyl elongation at 29 °C and no induction of *YUC2* after 6 h at 29 °C (Fig. 3E, F; Additional file 2: Table S1). Taken together, these results suggest that in addition to VIM1-dependent DNA methylation, the PcG-dependent histone methylation machinery also directly regulates *YUC2* expression in response to warm temperatures.

Considering the similarities between VIM1 and LHP1 behaviors at the *YUC2* locus, we wondered if VIM1-associated DNA methylation and LHP1-related H3K27me3 transcriptional regulations were dependent on each other. We observed that *YUC2* regulatory region in the proximal promoter displayed lower levels of cytosine methylation in the *lhp1* mutant at 23 °C, which resulted to be restored at 29 °C in *lhp1*, exhibiting opposite behavior to the WT (Fig. 3G) and the same trend as OE *APOLO-*1 and *vim1-3* (Fig. 2H). To further elucidate the link between LHP1 and VIM1, we assessed the levels of H3K27me3 and LHP1 capacity to bind to the *YUC2* promoter region in 4-day-old *vim1-3* seedlings. Notably, LHP1 binding and H3K27me3 levels were impaired in *vim1-3* (Fig. 3H, I). In combination, these results suggest that while *lhp1* mutation affects DNA methylation of a VIM1-target region, *vim1* mutation impairs LHP1 binding and H3K27me3 deposition on the same locus, hinting at a cooperative interaction between these two epigenetic factors. To test any direct cooperative interaction, we performed bimolecular fluorescence complementation (BiFC) assays in *N. benthamiana* leaves transiently transformed with *CYPF*-*VIM1* and *NYFP-LHP1*, which demonstrated that both proteins can interact in vivo (Fig. 3J; Additional file 1: Fig. S8). Altogether, our results established that the lncRNA *APOLO* associates with both a regulator of DNA methylation (VIM1) and a PcG component (LHP1), which further cooperate to mediate DNA methylation and H3K27me3 deposition at a specific *APOLO* target locus.

### Human lncRNA *UPAT* can exert similar regulatory functions as *APOLO* in planta

**Fig. 4:**
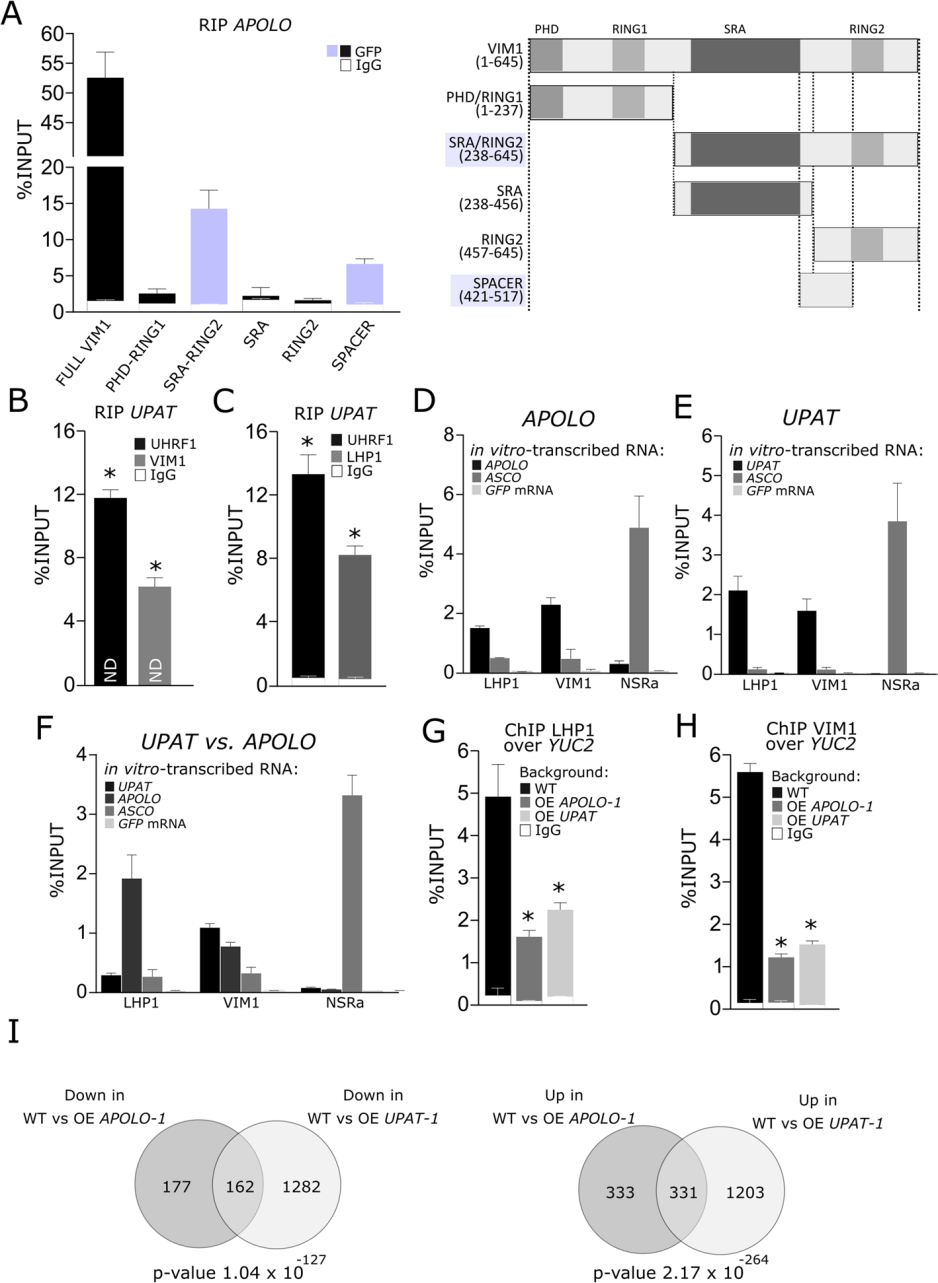
The UHRHF1-interacting lncRNA *UPAT* binds to VIM1 and LHP1 in plant cells and decoys the complex away from chromatin. **A** RNA immunoprecipitation (RIP) assay in *Nicotiana benthamiana* leaves transiently co-transformed with *APOLO* and translational fusions expressing *GFP-VIM1* or derivatives (*GFP-PHD*/*RING1*, *GFP-SRA/RING2*, *GFP-SRA*, *GFP-RING2*, *GFP-SPACER*) under the control of the 35S-CaMV promoter. Schematic representation of VIM1 protein and derivatives tested for RIP is shown on the right. Amino acid coordinates are indicated between brackets. **B**, **C** RIP assay in *N. benthamiana* leaves transiently co-transformed with *UPAT* and *GFP-UHRF1*, *GFP-VIM1***B**, or *LHP1-GFP***C** translational fusions expressed under the control of the 35S-CaMV promoter. **D**–**F** In vitro interaction of recombinant VIM1:GFP, LHP1: and the canonical RNA-binding protein NSRa, with *APOLO*, *UPAT*, GFP mRNA, and the *ASCO*-interacting lncRNA *ASCO*. Equimolar concentrations of each RNA (for 100 ng of *APOLO* as a reference) were incubated with purified proteins before proceeding with a regular RIP. **G**–**H** Chromatin immunoprecipitation (ChIP)-qPCR analysis of LHP1 (in **D**) and VIM1 (in **E**) binding at the *YUC2* promoter in wild-type (WT), *APOLO* over-expression (OE *APOLO-1*), and *UPAT* over-expression (OE *UPAT*) plants transiently transformed or not with *GFP-VIM1*. In **A**–**H**, results are expressed as a percentage of the INPUT fraction. Anti-IgG antibodies were used as a negative control. Bars represent average ± SD (*n* = 3 biological replicates). **I** Venn diagrams showing the overlap of upregulated and downregulated genes in plants constitutively expressing *APOLO* or *UPAT* vs. WT 4-day-old plants grown in standard conditions (23 °C). The *p*-value indicated below was calculated by hypergeometric test considering all genes annotated in Araport11. Fifty-three percent of common DEG are responsive to warmth in WT plants (Additional file 2: Table S5b)

Arabidopsis VIM1 is a homolog of the mammalian methylcytosine-binding protein UHRF1 [31, 64]. Interestingly, UHRF1 was shown to directly recognize in vivo the human lncRNA *UPAT*, through its flexible spacer region positioned between the SRA and RING domains [34, 65, 66]. This interaction stabilizes UHRF1 by interfering with its ubiquitination and subsequent degradation [34]. In order to determine if the capacity of VIM1 and UHRF1 to interact with lncRNAs is conserved between plants and animals, we first delimited the minimal region of VIM1 that interacts with *APOLO.* We generated independent GFP fusion constructs bearing different *VIM1* coding regions, according to Woo et al. [27] (Fig. 4A right panel). All the construct-encoded proteins accumulated exclusively or partially in the nucleus of *N. benthamiana* cells, with or without *APOLO* co-expression (Additional file 1: Fig. S9A-B). Similar to the reported interaction between UHRF1 and *UPAT* [34], an anti-GFP RIP performed in *N. benthamiana* leaves transiently co-transformed with *APOLO* and GFP-VIM1 derivatives revealed that the two VIM1 portions containing the full spacer region (GFP-SRA/RING2 and GFP-Spacer) bind to *APOLO* in vivo, although with lower efficiency than the full-length VIM1 (Fig. 4A). Thus, our results indicated that the lncRNAs-binding capacity of the SRA/RING-spacer regions of VIM1 and UHRF1 is evolutionary conserved between plants and animals.

Considering the common ability of the homologous VIM1 and UHRF1 to interact with lncRNAs, we wondered whether the lncRNA *UPAT*, exhibiting no apparent sequence homology with *APOLO* (Additional file 1: Fig. S9C) but potentially sharing functional secondary structures (predicted by using RNAfold; [67, 68]; Additional file 1: Fig. S9D), could also interact with VIM1 and its partner LHP1 in planta. Strikingly, an anti-GFP RIP in *N. benthamiana* leaves transiently co-transformed with *UPAT* and *GFP*-*UHRF1*, *GFP*-*VIM1*, or *LHP1-GFP* demonstrated that *UPAT* is able to interact with UHRF1, as well as with VIM1 and LHP1 in plant cells (Fig. 4B, C). In order to further characterize the interaction between VIM1 and LHP1 with *APOLO* and *UPAT*, we purified recombinant GFP-tagged proteins and incubated them with different combinations of in vitro-transcribed *APOLO*, *UPAT*, and *GFP* mRNA, and with the unrelated lncRNA *ASCO*, previously linked to alternative splicing regulation [69]. We compared VIM1 and LHP1 binding to each transcript with the *ASCO*-partner NSRa, a canonical RNA-binding protein [70]. VIM1 and LHP1 exhibited specific recognition of *APOLO* and *UPAT* over *GFP* mRNA and *ASCO*. Interestingly, in the presence of equivalent molar concentrations of *APOLO* and *UPAT*, LHP1 showed a preferential binding to *APOLO*, in contrast to VIM1 (Fig. 4D–F).

**Fig. 5:**
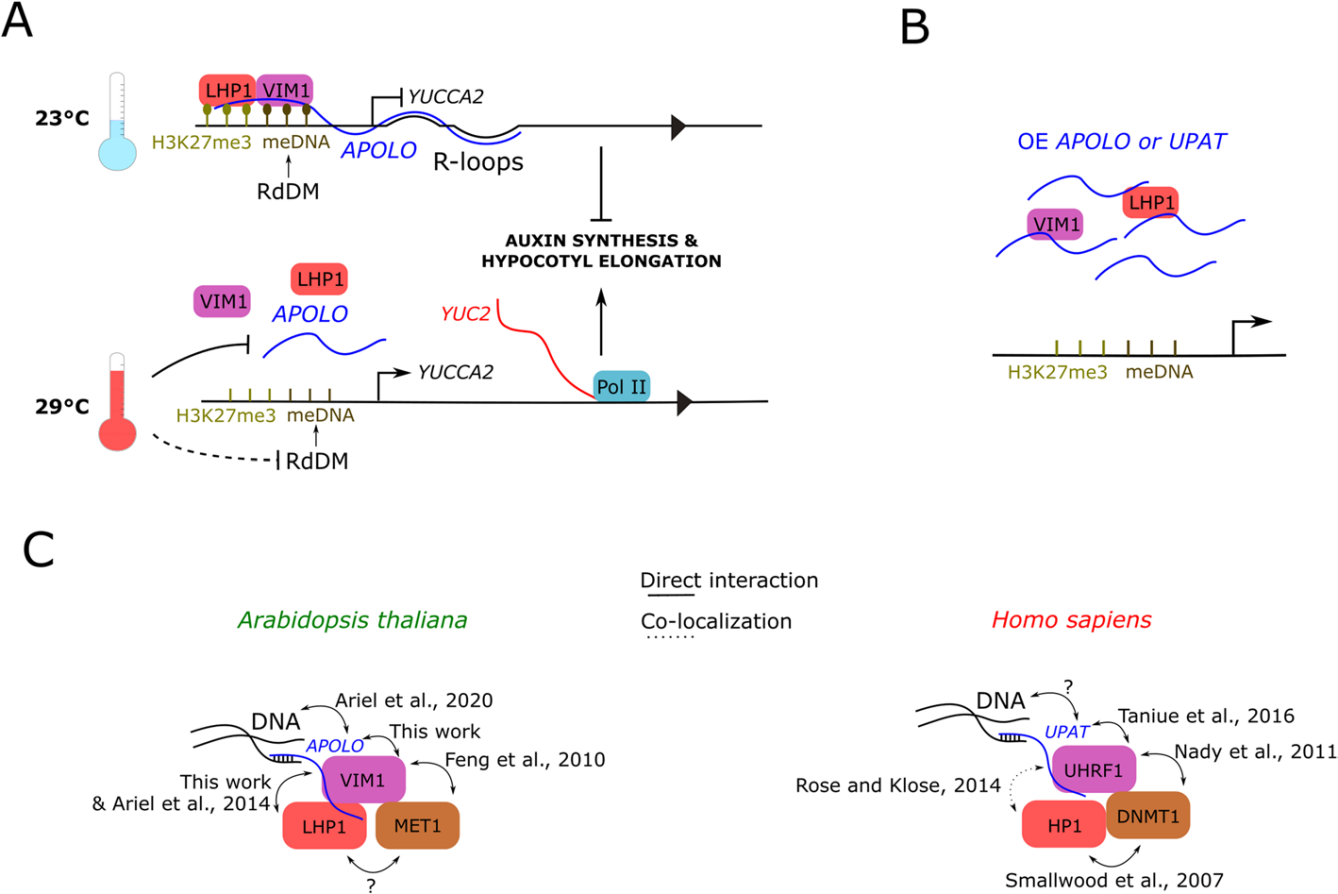
Long noncoding RNA-mediated regulation of DNA and histone methylation in plants and animals uncovers mechanistic commonalities across kingdoms. **A** Model for the regulation of *YUC2* expression in response to heat by the *APOLO*-VIM1-LHP1 complex: at 23 °C, *APOLO* lncRNA recognizes the *YUCCA2* (*YUC2*) locus by sequence complementarity and R-loop formation. *APOLO* interacts with VIM1 and LHP1 over the *YUC2* promoter region, which exhibits RdDM and H3K27me3 deposition, blocking *YUC2* transcription. meDNA (light brown balls) and H3K27me3 (dark brown balls) are cooperatively maintained by the VIM1-LHP1 complex. At 29 °C, *APOLO* transcript levels decrease and VIM1-LHP1 binding to the *YUC2* promoter region is reduced. Conjointly, 24nt siRNA accumulation is decreased, impairing RdDM over the *YUC2* promoter. As a result, *YUC2* transcriptional activity increases. YUC2 participates in auxin synthesis, promoting hypocotyl elongation in response to heat. **B** Proposed explanation for the molecular impact of *APOLO* or *UPAT* constitutive expression. Although the ability of *UPAT* to form R-loops in animals (or when expressed in plants) remains unknown, high levels of both lncRNAs in Arabidopsis achieve to decoy VIM1 and LHP1 away from chromatin, hinting at a stoichiometrically modulated mechanism. **C** Comparison between conserved lncRNA-integrated epigenetic machineries across kingdoms. Homolog proteins between *Homo sapiens* and *Arabidopsis thaliana* are shown in the same color. References of the articles demonstrating direct interactions or co-localizations are indicated. Question marks “?” mean that the interaction has not been proved yet

Finally, we assessed if the constitutive expression of *UPAT* or *APOLO* could modulate VIM1 and LHP1 binding to *YUC2* promoter. ChIP-qPCR analyses performed in WT, *OE APOLO-1*, and *OE UPAT* Arabidopsis stable lines transiently transformed or not with *GFP-VIM1* (Additional file 1: Fig. S1B) revealed that both *APOLO* and *UPAT* over-expression impairs the interaction of VIM1 and LHP1 with *YUC2* promoter (Fig. 4G, H). Furthermore, *UPAT* constitutive expression in stable Arabidopsis seedlings resulted in decreased transcript levels of *YUC2* (Additional file 1: Fig. S10A-B). Moreover, by performing a new RNA-Seq comparing the transcriptional profile of WT vs. OE *APOLO-1* and OE *UPAT-1* plants, a significant overlap between *APOLO* and *UPAT*-deregulated genes emerged (both for up and downregulated genes), including 53% of common differentially expressed genes (DEG) which are modulated by warmth, strongly suggesting that they exert similar molecular mechanisms (Fig. 4I; Additional file 2: Table S5a and b). Altogether, our results revealed a functional interaction between sequence-unrelated long noncoding transcripts and the key epigenetic regulators LHP1 and VIM1, which are remarkably conserved across kingdoms (Fig. 5 for a model in plants and a comparison of *UPAT* and *APOLO* integrated complexes across kingdoms).

## Discussion

### Epigenetic transcriptional reprogramming in the thermomorphogenic response

When plants are exposed to warmer nonstressful temperatures, some organs grow and develop at a faster rate without affecting their final dimensions, whereas others suffer morphological or developmental changes. The latest response is known as thermomorphogenesis and includes petiole elongation, leaf hyponasty, and auxin-dependent hypocotyl elongation [71–73]. These modifications of the plant architecture rely on a deep transcriptional reprogramming determined by dynamic changes of chromatin organization [74–79].

A strong correlation between temperature and DNA methylation was first established in Swedish *A. thaliana* accessions, where higher levels of CpHpH methylation were observed in plants grown at 16 °C compared to 10 °C [80, 81]. Interestingly, CpHpH methylation is conjointly regulated by CMT2 and the RdDM pathway [24–26] and both have been involved in the heat response. It was shown that *cmt2* mutants are more tolerant to heat stress [82] and that the heat-stress response relies on the integrity of the RdDM pathway [83, 84]. In agreement, we observed that *cmt2* mutants displayed a slight but significantly increased response to warm temperatures in contrast to RdDM mutants exhibiting hypocotyl elongation defects. We also observed that *met1* mutants exhibited the same developmental phenotype as RdDM mutants. Accordingly, it was demonstrated that *met1* knockout causes a loss of DNA methylation, a global loss of Pol V at its normal locations and a Pol V redistribution to sites that become hypermethylated [62]. Furthermore, we observed a reduction in CpHpH methylation at the *YUC2* promoter in the *vim1-3* mutant, linking the canonical CpG-related machinery with the RdDM pathway.

Importantly, *met1* was also shown to suffer H3K9 hypermethylation at PcG target genes and a redistribution of H3K27me3 to transposons [43], hinting at a relation between DNA methylation and PRC-dependent histone modifications. Consistently, we uncovered here a new molecular link between the DNA methylation-associated protein VIM1 and the H3K27me3-related protein LHP1, which can interact together and with the lncRNA *APOLO*. An indirect relation between LHP1 and VIM1 had already been described through the histone methyltransferase NtSET1, a common interactor. It was first shown in *Nicotiana tabacum* that NtSET1 directly interacts with LHP1 [85], and it was then reported that NtSET1 can bind to AtVIM1 in planta [86], although no link was established between LHP1 and DNA methylation.

Although VIM1, VIM2, and VIM3 have been reported to function redundantly to mediate epigenetic transcriptional silencing [28], we showed here that *vim1* single mutants exhibit particular molecular and related physiological phenotypes in response to warmth. The expression of *VIM1* is induced by warmth, whereas *VIM2* is repressed. Interestingly, we showed that although only VIM1 was first identified by ChIRP-MS as a potential *APOLO* partner, VIM2 is also able to weakly but significantly recognize *APOLO* in *N. benthamiana* leaves, suggesting that the VIM2-*APOLO* interaction may also occur during Arabidopsis development and that lncRNA-mediated regulation of VIM protein activity is a general feature in plants. Further research will be needed to uncover if VIM2 and VIM3 are also involved in thermomorphogenesis.

Here, we related the hypocotyl elongation defect observed in *vim1* to the transcriptional activation of the *YUC2* gene [58, 59], likely among other auxin-related genes emerging as potential *APOLO* and VIM1 common targets (Additional file 2: Table S3). The control of *APOLO* over auxin synthesis and transport may affect global auxin homeostasis exceeding *YUC2*. Therefore, as expected, we cannot mimic all *APOLO*-related phenotypes in the *yuc2* single mutant. *YUC2* regulation in response to heat was first observed in flowers, where heat shock (33 °C) repressed its expression and caused plant male sterility [59]. At milder temperatures (27 °C), *YUC2* transcript levels were reported to increase, in correlation with the demethylation of *YUC2* proximal promoter region targeted by RdDM [63], suggesting that cytosine methylation at the *YUC2* regulatory region is critical for the control of its expression in response to heat. In addition to DNA methylation, a correlation was established between the deposition of H3K27me3 and genes exhibiting either high or low transcription rates under warm temperatures [87]. Consistently, we observed that the *lhp1* mutant displayed impaired hypocotyl elongation and showed no activation of *YUC2* at 29 °C. Interestingly, LHP1 was previously reported to exert a positive role in the regulation of *YUCCA* genes under standard growth conditions. *YUC5*, *YUC8*, and *YUC9* displayed an abnormal transcriptional activity in the *lhp1* mutant [88], correlating with a reduction of H3K27me3, as revealed by genome-wide approaches [49]. In addition, LHP1 was found to localize to *YUC2*, whose expression was not affected by the *lhp1* mutation at this developmental stage, in an auxin-dependent fashion [88].

It was recently shown in Arabidopsis that the 5′ untranslated region of several mRNAs may adopt alternative hairpin conformations under warm cycling daytime temperatures, modulating translation efficiency. It was proposed that mRNA thermoswitches enhancing protein synthesis may constitute a conserved mechanism enabling plants to respond rapidly to high temperatures [89]. In contrast, the potential role of lncRNAs as thermosensors in higher organisms remains unexplored. In this work, we showed that *APOLO* participates in the thermomorphogenic response in Arabidopsis. Interestingly, *APOLO* transcriptional accumulation increases in response to cold [53], whereas we showed here that it decreases under warm temperatures. Further research will be needed to uncover the effect of temperature on the structure of *APOLO* and other lncRNAs that determines their molecular role and contributes to the plant adaptation to environmental constraints.

### R-loop-associated long noncoding RNAs modulate DNA methylation and histone modifications

RNA-DNA hybrids (R-loops) have been identified as important regulators of chromatin conformation and gene transcriptional regulation [90, 91]. *APOLO* is known to recognize its multiple *trans* targets by sequence complementarity and R-loop formation. Upon interaction, it decoys LHP1, shaping local chromatin 3D conformation and subsequently modulating gene transcriptional activity [52]. Here, we showed that *APOLO* recognizes *YUC2* gene body through the formation of R-loops. Recently, it was shown that the human lncRNA *HOTTIP* can form R-loops in *trans* at the base of CTCF-related topologically associated domains (TADs), suggesting that lncRNA-DNA duplexes may exert a general role in 3D chromatin conformation [92].

Interestingly, *APOLO* additionally interacts with VIM1 to co-regulate DNA methylation at the *YUC2* promoter, mediating *YUC2* transcriptional response to warm temperatures. Although the involvement of lncRNAs in the regulation of DNA methylation in plants remains largely unknown, previous reports have linked lncRNA activity to promoter methylation in mammals, through the interaction with protein partners [93] or the formation of R-loops [94, 95]. Notably, it was reported that DNMT1-interacting lncRNAs can promote or block local DNA methylation [93, 96, 97], whereas it was proposed that R-loops formed by nascent transcripts can preclude DNA methylation from promoter regions by repelling DNA methyltransferases [98, 99].

In addition to DNA methylation, a link between R-loops and the activity of PRC complexes has also been proposed. In mammals, it was shown that R-loops can form at a subset of PcG target genes, resulting in alternative regulatory outputs depending on the interplay between the DNA-RNA duplex and PRC components [100]. In this work, we showed that the over-accumulation of *APOLO* can titer VIM1 and LHP1 away from the *YUC2* promoter region. It is thus tempting to hypothesize that R-loops formed by *APOLO* at the *YUC2* locus stoichiometrically modulate VIM1 and LHP1 recruitment to DNA in response to changes in ambient temperature. At 23 °C, a precise amount of *APOLO* over the *YUC2* locus may guide the LHP1-VIM1 complex to the target region. In contrast, at 29 °C *APOLO* transcript levels decrease, hindering VIM1 and LHP1 efficient recognition of *YUC2*. On the other hand, over-accumulation of *APOLO* can decoy LHP1 and VIM1 away from the *YUC2* locus, explaining the similar hypocotyl elongation phenotypes of OE *APOLO*, *vim1*, and *lhp1*, as well as the similar DNA methylation levels observed at the *YUC2* promoter in these three backgrounds (Fig. 5A, B). The re-methylation of the *YUC2* promoter in OE *APOLO*, *vim1*, and *lhp1* at warm temperatures, in contrast to WT, might be due to altered RdDM activity when any component of the regulatory complex is affected. Thereby, we propose that *APOLO* functions as a key mediator of the PRC and DNA methylation machineries, hinting at a stoichiometric factor fine-tuning the activity of R-loop-related lncRNAs.

### Long noncoding RNAs as key mediators of the Polycomb and DNA methylation machineries

Connections between Polycomb and DNA methylation machineries have been reported in few studies in mammals. DNMT1 was shown to directly interact with HP1 to mediate silencing of euchromatic genes [101], and its enzymatic activity is also stimulated by direct interaction with UHRF1 [102–106]. Interestingly, HP1 and UHFR1 have been shown to co-exist on chromatin [107], although their direct interaction has not been proven. Here, we showed that LHP1 and VIM1 interact in planta, suggesting that a direct interaction between their homologs HP1 and UHRF1 may occur in mammals. As in mammals, it was shown in Arabidopsis that VIM1 recruits MET1 to hemi-methylated DNA [40, 44, 64, 108], hinting at the existence of analogous complexes formed by MET1/DNMT1, VIM1/UHRF1, and LHP1/HP1 in plants and animals (Fig. 5C).

Remarkably, DNMT1, UHRF1, and HP1 were all shown to interact with coding or long noncoding RNAs [34, 97, 109, 110], whereas only LHP1 was previously shown to bind to RNAs in vitro [50] and in vivo [51, 52, 111] in plants. Here, we uncovered the interaction between VIM1 and lncRNAs. Notably, UHRF1 can interact with the lncRNA *UPAT* whose scaffold function prevents UHRF1 from ubiquitination and proteasomal degradation [34]. Knockdown of *UPAT* results in a drastic decrease in the levels of DNMT1 protein and a reduction of hemimethylation at its target genes [34]. Here, we showed that *UPAT* can interact with VIM1 and LHP1 in plant cells, and high amounts of *UPAT* can decoy VIM1 and LHP1 from *YUC2* promoter, as the sequence-unrelated transcript *APOLO*. Furthermore, the transcriptomics of plants constitutively expressing *UPAT* significantly resemble the output of *APOLO* over-expression, further supporting that common molecular mechanisms can be mediated by both noncoding transcripts.

Although a global anti-correlation is observed in the Arabidopsis genome between DNA methylation and H3K27me3 deposition, characterizing heterochromatic and euchromatic regions respectively [11, 43, 45, 112], DNA hypomethylation was associated to H3K27me3 reduction at specific genes in *met1* and *vim1/2/3* mutants [43, 44]. The increasing evidence that PRC2 also targets transposons across distantly related eukaryotic lineages has recently served to hypothesize that an ancestral role of Polycomb is linked to silencing repetitive genomic elements [113]. Consistently, our results indicate that *APOLO* lncRNA interacts with LHP1 and VIM1 to regulate *YUC2*, constituting a lncRNA-mediated non-canonical cooperative interaction between Polycomb and DNA methylation machineries. Here we showed that H3K27me3 deposition and LHP1 binding to *YUC2* was impaired in *vim1* mutants, whereas DNA methylation was altered in *lhp1* mutants. We cannot exclude that indirect effects mediated by transcriptional activation of the *YUC2* gene may be affecting the interplay between the two epigenetic marks. Although other lncRNAs have been linked to thermomorphogenesis or light-dependent hypocotyl elongation, their mechanisms of action or target recognition remain unknown [114, 115]. Considering that the sequence-unrelated lncRNA *UPAT* mimicked the action of *APOLO* over the chromatin-binding capacity of the VIM1-LHP1 complex and global transcriptional reprogramming, it is likely that additional yet-uncovered lncRNAs exert a similar role as *APOLO* in Arabidopsis.

## Conclusions

Altogether, our results hint at a mechanism possibly conserved across kingdoms, which is likely based on the structure rather than the sequence of long noncoding transcripts. A deeper knowledge about the molecular basis behind lncRNA-related regulatory mechanisms will likely push back the frontiers of human therapeutics and will allow the design of innovative strategies for sustainable agriculture in a climate change context.

## Methods

### Plant material and growth conditions

All the *Arabidopsis thaliana* lines used were in the Columbia-0 (Col-0) background. *vim1-2* (SALK_050903) and *vim1-3* (SALK_149277c) seeds were obtained from the Arabidopsis Biological Resource Center (http://www.arabidopsis.org/). Mutant plants were genotyped by PCR using specific primers to amplify the endogenous locus and T-DNA borders (primers used are listed in Additional file 2: Table S4). *VIM1* and *UPAT* over-expression (OE), as well as CRISPR-Cas9-*APOLO* transgenic plants, were generated through *Agrobacterium tumefaciens* (strain EHA105 or ASE containing the pSOUP helper plasmid respectively)-mediated transformation [116]. Two independent lines of transformants harboring *GFP-VIM1* or *UPAT* were selected on MS/2 medium supplemented with kanamycin (40 μg/mL). *VIM1* expression levels were measured by RT-qPCR (primers used are listed in Additional file 2: Table S4). Transgenic CRISPR-Cas9-*APOLO* lines were selected for Basta resistance. T2 plants were genotyped for heterozygous deletion of *APOLO*, and T3 plants were selected for homozygous deletion of *APOLO* (confirmed by PCR followed by Sanger sequencing) and for the absence of T-DNA (primers used are listed in Additional file 2: Table S4).

Seeds were surface-sterilized by treatments with 70% EtOH and 5% hypochloryte-1% SDS, washed, and stratified at 4 °C for 2 days to obtain homogeneous germination. Seedlings were grown at 23 °C or 29 °C on solid half-strength Murashige and Skoog (MS/2) medium (Duchefa), under long day conditions (16 h light, 95 μE m^−2^ s^−1^/8 h dark, ARALAB Fitoclima 600, LED light). For phenotypic characterization of hypocotyl elongation, seedlings were grown for 3 h at 23 °C and transferred to 29 °C or maintained at 23 °C for 4 days. Hypocotyl lengths were measured using the ImageJ software. For RNA sequencing, chromatin immunoprecipitation, and methylated DNA immunoprecipitation assays performed in Arabidopsis stable lines, seedlings were grown for 4 days at 23 °C and transferred to 29 °C or maintained at 23 °C for 6 h. For chromatin isolation by RNA purification and DNA-RNA immunoprecipitation, seedlings were grown for 11 days at 23 °C.

### Cloning procedures

The entire coding region of *VIM1* (AT1G57820), *VIM1* derivatives (PHD/RING1, SRA/RING2, SRA, RING2; [27]), *LHP1* (AT5G17690), and the lncRNA *UPAT* [34] were amplified by PCR on genomic DNA or pBluescript:UPAT vector [34] respectively and cloned into the Gateway entry vector pENTR/D-TOPO (Invitrogen). The entire coding region of *VIM2* (AT1G66050) was amplified by PCR on cDNA and cloned into the pDONOR207 vector by Gateway technology (BP reaction) (Invitrogen). Entry clones were recombined by Gateway technology (LR reaction) into the pK7WGF2, pK7FWG2, or pK7WG2 vectors containing respectively a p35S-GFP-GW, a p35S-GW-GFP, or a p35S-GW cassette (http://www.psb.ugent.be/gateway/index.php). pENTR/VIM1, pENTR/SRA/RING2, and pENTR223/LHP1 were recombined by LR reaction into the pGPTVII.Bar-C/NYFP vectors containing respectively a p35S-CYFP or a p35S-NYFP cassette (http://www.psb.ugent.be/gateway/index.php). The spacer of *VIM1* and the entire coding region of *UHFR1* were amplified by PCR on cDNA and pGE-HIS-MBP *UHRF1* plasmid respectively and cloned into the GreenGate entry vector pGGC000. Destination vectors were constructed associating the modules: 35S promoter, GFP, Ubiquitin 10 terminator, and pNOS:BastaR, tNOS, according to Lampropoulos et al. [117]. p35S-mCherry was constructed associating the modules: 35S promoter, mCherry, and NOS terminator [117]. All Gateway constructs were subsequently transformed into the *A. tumefaciens* strain EHA105 and GreenGate constructs into the *A. tumefaciens* strain ASE containing a pSOUP helper plasmid.

To generate the CRISPR/Cas9 *APOLO* system, modified Greengate vectors [117] were used. A fragment containing two BpiI recognition sites between the AtU6-26 promotor and the sgRNA scaffold was synthetize by the Eurofins genomics service and further subcloned into pGGA000, pGGB000, pGGC000, and pGGD000. The obtained pGGA010, pGGB010, pGGC010, or pGGD010 plasmids were further used to clone specific guide RNA sequences (20 nt in length-NGG) in a one-step digestion-ligation reaction with the BpiI restriction enzyme. Four specific guide RNAs targeting *APOLO* genomic sequence were designed using the TEFOR website (http://crispor.tefor.net/crispor.py). For each sgRNA, forward and reverse primers were designed with complementary overhangs to BpiI digested plasmids, annealed, and subcloned into pGGA/B/C/D010 vectors (primers used are listed in Additional file 2: Table S4). Cas9 sequence [118, 119] from Streptococcus pyogenes (SpCas9) was subcloned in pGGE000 under the control of an AtUbi10 promotor using the pDe-CAS9 vector [118] as a matrix and 5′-TGTGAAGACAACCATGGATAAGAAGTACTCTATC-3′ and 5′-TGTGAAGACTTTAGTAAGCCTATACTGTACTTAAC-3′ as forward and reverse primers to create the pGGE011 entry plasmid. The four pGGA/B/C/D-sgRNA specific entry vectors, pGGE011, and pGGF008 (containing a Basta resistance cassette) were subsequently assembled into the pGGZ001 destination vector in a one-step digestion-ligation reaction using BsaI (Additional file 1: Fig. S11).

### Transient expression and confocal microscopy

Leaves of *Nicotiana benthamiana* were transiently transformed as described in Waadt and Kudla [120]. For RNA immunoprecipitation, *GFP-VIM1* or derivatives (*GFP-PHD/RING1*, *GFP-SRA/RING2*, *GFP-SRA*, *GFP-RING2*, and *GFP-Spacer*) were co-transformed with p35S-*APOLO* [52], or *GFP-UHRF1*, *GFP-VIM1*, or *LHP1-GFP* were co-transformed with p35S-*UPAT*. For sub-cellular localization, VIM1 protein or derivatives were co-transformed or not with p35S-*APOLO*. For bimolecular fluorescence complementation, *YFPC-VIM1* or *YFPC-SRA/RING2* were co-transformed with *YFPN-LHP1*. mcherry was co-transformed and used as a membrane and nuclear marker. Leaves were harvested or cells were analyzed 2 days after infiltration using a Zeiss confocal microscope equipped with Plan-Apochromat 10x/NA 0,45/M27 or Plan-Apochromat 20x/NA 0,8/r M27 US-VIS-IR dry lens. eGFP, eYFP, and mCherry fluorescence were excited with 488- and 514-nm argon laser lines and 561-nm diode, emission recorded between 490 and 580 nm, 520 and 600 nm, and 580 and 640 nm respectively. Image acquisitions and analyses were performed on the IPS2 Imaging Facility.

Leaves of 3-week-old *Arabidopsis thaliana* were transiently transformed as described in Zhang et al. [121]. Transformation with *GFP-VIM1* was carried out in leaves > 0.5 cm in length, in 10 to 15 plants per genotype (WT, OE *APOLO-1*, and OE *UPAT*). Leaves were crosslinked and harvested (for ChiP) or cells were analyzed 3 days after infiltration, using a Leica TCS SP8 confocal laser scanning microscope. eGFP was excited at 488 nm (intensity=8%) and emission recorded between 495 and 530 nm for GFP and 610 and 670 nm (gain 650) for chlorophyll fluorescence. Images were processed using the Fiji software [122].

### Chromatin isolation by RNA purification and mass spectrometry or dot blot

ChIRP-qPCR was performed as previously described [51, 52]. Formaldehyde-crosslinked seedlings were ground, and nuclei were isolated and sonicated using a water bath Bioruptor Pico (Diagenode; 30 s on/ 30 s off pulses, at high intensity for 10 cycles). *APOLO*-associated chromatin was purified using 100 pmol of two independent sets of primers (ODD and EVEN) and a negative control (primers matching the LacZ RNA), respectively [51, 52]. Primers used are listed in Additional file 2: Table S4.

A method adapted from the ChIRP protocol [123] was developed to allow identification of plant nuclear proteins bound to specific lncRNAs, as described in Rigo et al. [69]. Briefly, plants were in vivo crosslinked, and cell nuclei were purified and extracted through sonication. The resulting supernatant was hybridized against biotinylated complementary oligonucleotides that tile the lncRNA of interest, and putative lncRNA-containing protein complexes were isolated using magnetic streptavidin beads. Co-purified ribonucleoprotein complexes were eluted and used to purify RNA or proteins, which were later subject to downstream assays for identification and quantification.

### Crosslinking and ribonucleoprotein complex purification

For protein extraction, approximately 250 g of 7-day-old Col-0 plants grown on solid half-strength MS medium was irradiated three times with UV using a CROSSLINKERCL-508 (Uvitec) at 0.400 J/ cm^2^. For RNA extraction, 10 g of 7-day-old Col-0 plants grown on solid MS/2 medium was crosslinked under vacuum for 15 min with 37 ml of 1% (v/v) formaldehyde. The reaction was stopped by adding 2.5 ml of 2M glycine, and seedlings were rinsed with Milli-Q purified water. For both crosslinking methods, 6 g of the fixed material was ground in liquid nitrogen (representing 15 ml of plant material ground to fine dust) and added to 50-ml tubes with 25 ml of extraction buffer 1– (the nuclei were prepared starting with 30 tubes; buffer 1: 10 mM Tris–HCl pH 8, 0.4 M sucrose, 10 mM MgCl_2_, 5mM β-mercaptoethanol, 1 ml/30 g of sample powder Plant Protease Inhibitor Sigma P9599). The solution was then filtered through Miracloth membrane (Sefar) into a new tube and 5 ml of extraction buffer 2 (10mM Tris–HCl pH8, 0.25 M sucrose, 10 mM MgCl_2_, 5 mM β-mercaptoethanol, 1% Triton X-100, 10 μl 100 μl PMSF) was added. The solution was then centrifuged, the supernatant discarded, and the pellet was resuspended in 500 μl of extraction buffer 3 (10 mM Tris–HCl pH 8, 1.7 M sucrose, 2 mM MgCl_2_, 5 mM β-mercaptoethanol, 0.15% Triton X-100, 50 μl protease inhibitor) and layered on top of 600 μl of fresh extraction buffer 3 in a new tube. After centrifugation at 13000 rpm for 2 min at 4 °C to pellet the nuclei, the supernatant was discarded and the pellet resuspended in 300 μl of nuclei lysis buffer (50 mM Tris–HCl pH7, 1% SDS, 10 mM EDTA, 1 mM DTT, 50 μl protease inhibitor, 10 μl RNAse inhibitor per tube) to degrade nuclear membranes. Samples were sonicated three times in refrigerated Bioruptor Plus (Diagenode), 30 cycles 30 s ON/30 s OFF in a Diagenode TPX microtube M-50001. After centrifugation, the supernatant was transferred to a new tube and diluted two times volume in hybridization buffer (50 mM Tris–HCl pH 7, 750 mM NaCl, 1% SDS, 15% formamide, 1 mM DTT, 50 μl protease inhibitor, 10 μl RNAse inhibitor). One hundred picpmoles of probes against *APOLO* (ODD and EVEN set of probes, [51, 52]) and the corresponding negative set against LacZ were added to samples and incubated 4 h at 50°C in a thermocycler. Samples were transferred to tubes containing Dynabeads- Streptavidin C1 (Thermo Fisher Scientific) and incubated 1 h at 50 °C. Then, samples were placed on a magnetic field and washed three times with 1 ml of wash buffer (2× SSC, 0.5% SDS, 1 mMDTT, 100 μl protease inhibitor). Protein purification samples for protein extraction were DNase-treated according to the manufacturer (Thermo Scientific). After addition of 1.8 ml of TCA-acetone (5 ml 6.1N TCA + 45 ml acetone + 35 μl β-mercaptoethanol), samples were incubated overnight at −80 °C. After centrifugation at 20,000 rpm for 20 min at 4 °C, the supernatant was discarded and 1.8 ml of acetone wash buffer (120 ml acetone, 84 μl β-mercaptoethanol) was added to the samples. Then, samples were incubated 1 h at −20 °C and centrifuged again at 20,000 rpm for 20 min at 4 °C. The supernatant was discarded, and the dry pellet was used for mass spectrometry analyses.

For ChIRP-dot blot analyses, three independent samples of 10 g each of *OE VIM1-1* seedlings were used for *APOLO*-ChIRP. Co-purified proteins without precipitation were deposed on PVDF membrane (Roche, Basel, Switzerland) pre-activated with methanol. Twenty microliters of ODD, EVEN, and LacZ samples was deposed, and the same amount of a 1/50 dilution of the input fraction, to avoid saturation of the signal. Blots were hybridized with polyclonal rabbit antibodies against GFP (Abcam ab290; dilution 1:1,000) and developed with anti-rabbit immunoglobulin (Agrisera, dilution 1:15,000) conjugated with horseradish peroxidase using the AgriseraECL SuperBright western blot detection reagent.

### DNA-RNA immunoprecipitation

DNA-RNA immunoprecipitation (DRIP) was performed as described in Ariel et al. [52]. Non-crosslinked seedlings were used for nuclei purification, and samples were sonicated using a water bath Bioruptor Pico (Diagenode; 30 s on/ 30 s off pulses, at high intensity for 4 cycles). Chromatin samples were incubated with 50 ml of washed Protein G Dynabeads pre-coated with 1 mg of S9.6 antibody (Millipore MABE1095) for 16 h at 4 °C. Samples treated with RNAseH (Invitrogen) for 2 h at 37 °C were used for DRIP in parallel as a negative control. After washing, DNA was recovered for qPCR over R-loop-related loci (primers used are listed in Additional file 2: Table S4).

### RNA sequencing analysis

Total RNA was prepared from 4-day-old *A. thaliana* wild-type (WT), OE *APOLO*-1 [51], and *vim1-3* seedlings (grown in ARALAB Fitoclima 600, 95 μE m^−2^ s^−1^ of LED light) using the QIAGEN RNeasy plant mini kit and treated with DNase (Fermentas). Three independent biological replicates were produced per genotype. For the RNA-Seq of WT, OE *APOLO*-1, and OE *UPAT*-1 (Fig. 4, Additional file 2: Table S5), plants were grown in ARALAB Fitoclima 600, 95 μE m^−2^ s^−1^ of fluorescent light. After RNA extraction, libraries were constructed using the Tru-Seq Stranded mRNA Sample Prep kit (Illumina®). Sequencing was carried out at the IPS2. The Illumina NextSeq500 technology was used to perform 75-bp sequencing. A minimum of 15 million of single end reads by sample was generated. RNA-seq preprocessing included trimming library adapters, and quality controls were performed with Trimmomatic [124]. Paired end reads with Phred Quality Score Qscore > 20 and read length > 30 bases were kept, and ribosomal RNA sequences were removed with SortMeRNA [125]. Processed reads were aligned using STAR with the following arguments: --alignIntronMin 20 --alignIntronMax 3000 --outSAMtype BAM SortedByCoordinate --alignIntronMax 3000 --outSAMtype BAM SortedByCoordinate --alignIntronMax 3000 --outSAMtype BAM SortedByCoordinate. Read overlapping exons per genes were counted using the featureCounts of the subreads package using the GTF annotation files from the Araport11 project [126]. Significance of differential gene expression was estimated using DEseq2 [127] and the FDR correction of the *p*-value was used during pairwise comparison between genotypes. A gene was declared differentially expressed if its adjusted *p*-value (FDR) was < 0.01. Heatmap was generated using log2 transformed fold change values compared WT 23 °C and computed with the pheatmap R [128].

### RNA immunoprecipitation assay

RNA immunoprecipitation (RIP) assays were performed on transiently transformed *N. benthamiana* leaves as described in Sorenson and Bailey-Serres [129], or in 2-week-old *A. thaliana* OE *VIM1.1* seedlings as described in Bardou et al. [70], using anti-GFP (Abcam ab290) and anti-IgG (Abcam ab6702). RIP was performed using Invitrogen Protein A Dynabeads. Precipitated RNAs were prepared using TRI Reagent (Sigma-Aldrich), treated with DNaseI (Fermentas) and subjected to RT-qPCR (High Capacity cDNA Reverse Transcription Kit (Thermo); primers used are listed in Additional file 2: Table S4). Total RNAs were processed in parallel and considered as the input sample.

### Chromatin immunoprecipitation assay

Chromatin immunoprecipitation (ChIP) assays were performed using anti-GFP (Abcam ab290), anti-LHP1 (Covalab pab0923-P), anti-H3K27me3 (Diagenode pab-195-050), and anti-IgG (Abcam ab6702), as described in Ariel et al. [52]. Crosslinked chromatin was sonicated using a water bath Bioruptor Pico (Diagenode; 30 s ON/30 s OFF pulses; 10 cycles; high intensity). ChIP was performed using Invitrogen Protein A Dynabeads. Precipitated DNA was recovered using Phenol:Chloroform:Isoamilic Acid (25:24:1; Sigma) and subjected to RT-qPCR (primers used are listed in Additional file 2: Table S4). Untreated sonicated chromatin was processed in parallel and considered as the input sample.

### In vitro protein-RNA interaction assay

Recombinant VIM1:GFP, LHP1:GFP, and NSRa:GFP were purified from 21-day-old stably transformed Arabidopsis plant leaves without crosslinking. Nuclei were isolated as previously described and immunoprecipitation was performed using the anti-GFP Abcam ab290 antibody and 20 μg of RNAseA (Thermo) and 10 U of DNAseI (Fermentas) per sample. Protein purification included astringent washes as for histone ChIP protocol [52]. For in vitro transcription of the *APOLO*, *UPAT*, *ASCO*, and *GFP* RNAs, 1 μg of purified DNA of each template including the T7 promoter at the 5′ end was used following the manufacturer instructions (HiScribe T7 High Yield RNA Synthesis kit, NEB). One hundred nanograms of each of the corresponding RNAs was incubated with protein A Dynabeads (Thermo) containing the recombinant purified proteins, as previously described [70]. After 1 h of incubation at 4 °C in binding buffer 1X (10 mM HEPES, pH 7.0; 50 mM KCl; 10% glycerol; 1 mM EDTA; 1 mM dithiothreitol; 0.5% Triton X-100), three 5-min washes were done with the same buffer in rotation at 4 °C. Precipitated RNAs were purified using TRI Reagent (Sigma-Aldrich), treated with DNaseI (Fermentas) and subjected to RT-qPCR (High Capacity cDNA Reverse Transcription Kit (Thermo); primers used are listed in Additional file 2: Table S4).

### Methylated DNA immunopreciptation

Methylated DNA immunoprecipitation (MedIP) assays were performed using anti 5-mC (Diagenode Mab-081-100) and anti-IgG (Abcam ab6702), as described in Nagymihály et al. [130]. Genomic DNA (1 μg) was sonicated using a water bath Bioruptor Pico (Diagenode; 30 s on/30 s off pulses; 4 cycles; high intensity). MedIP was performed using Invitrogen Protein G Dynabeads. Precipitated DNA was recovered using Phenol:Chloroform:Isoamilic Acid (25:24:1; Sigma) and subjected to RT-qPCR (primers used are listed in Additional file 2: Table S4). Untreated sonicated genomic DNA was processed in parallel and considered as the input sample.

### RT-qPCR

RT-qPCR were performed using the LightCycler 480 SYBR Green I Master Kit on a StepOne Plus apparatus (Applied Biosystems) using standard protocols (40 to 45 cycles, 60 °C annealing). *PP2A* (AT1G13320) was used for normalization (primers used are listed in Additional file 2: Table S4) for ∆∆Ct quantification method. The efficiency of all primers was verified by consecutive dilutions of standardized samples. *PP2A* exhibited a homogenous behavior in all the RNA-Seq approaches included in this work (WT 23 or 29 °C, OE *APOLO*, *vim1-3*, and OE *UPAT* plants).

### Histochemical staining

Four-day-old *pYUCCA2:GUS* transgenic seedlings [60] grown at 23 or 29°C were infiltrated with GUS staining solution (0.1 M phosphate buffer pH 7.5; 100 mM K_3_Fe(CN)_6_; 100 mM K_4_Fe(CN)_6_; 20% Triton X-100; 50 mM X-Gluc) under vacuum and subsequently incubated at 37 °C for 12 h. Stained tissues were cleared in 70% EtOH for 24 h at 37 °C and observed under a light microscope.

### Statistical analyses

Statistical analyses were performed with non-parametric tests, Mann–Whitney when *n*=2 independent groups, and Kruskal-Wallis test when *n*>2 independent groups.

## Supplementary Information

Additional file 1: Figure S1. GFP-VIM1 localizes at the nucleus. Figure S2. Characterization of *Arabidopsis thaliana VIM1* over-expression and *vim1* homozygous T-DNA insertion lines. Figure S3. *VIM1*, *VIM2* and *VIM3* are differentially regulated in reponse to heat. Figure S4. *APOLO* and VIM1 regulate hypocotyle elongation in response to heat. Figure S5. Gene categories transcriptionally regulated in thermomorphogenesis. Figure S6. Epigenetic profile of the *YUCCA2* locus. Figure S7. de novo DNA methylation and its maintenance regulate thermomorphogenesis. Figure S8. Bimolecular Fluorescence Complementation (BiFC) assay in transiently transformed *Nicotiana benthamiana* leaves. Figure S9. *APOLO* and *UPAT* lncRNAs share mechanisms of interaction with methylcytosine-binding proteins but no sequence similarity [133]. Figure S10. Constitutive expression of the lncRNA *UPAT* in Arabidopsis seedlings impairs *YUCCA2* transcriptional accumulation. Figure S11. CRISPR/Cas9 strategy for *APOLO* deletion.

Additional file 2: Table S1. Quantification of hypocotyl length of Arabidopsis over-expression or mutant lines grown at 23°C or 29°C. Table S2. Lists of differentially expressed genes in WT (23°C vs 29°C), *OE APOLO-1* and *vim1-3*. Table S3. *APOLO* targets a subset of genes differentially methylated in the *vim1* mutant. Table S4. List of primers used in this study. Table S5. Lists of differentially expressed genes in WT, *OE APOLO-1* and *OE UPAT-1* plants.

Additional file 3. Peer review history.

## Data Availability

RNA sequencing data have been deposited in GEO with the accession codes GSE167879 [131] and GSE210828 [132]. The corresponding author should be contacted for the request of the materials related to this manuscript.

## References

[1] Yu M, Ren B (2017). The three-dimensional organization of mammalian genomes. Annu Rev Cell Dev Biol.

[2] Doğan ES, Liu C (2018). Three-dimensional chromatin packing and positioning of plant genomes. Nat Plants.

[3] Tamaru H, Selker EU (2001). A histone H3 methyltransferase controls DNA methylation in Neurospora crassa. Nature.

[4] Jaenisch R, Bird A (2003). Epigenetic regulation of gene expression: how the genome integrates intrinsic and environmental signals. Nat Genet.

[5] Matzke MA, Birchler JA (2005). RNAi-mediated pathways in the nucleus. Nat Rev Genet.

[6] Lister R, Pelizzola M, Dowen R, Hawkins R, Hon G, Nery J (2009). Human DNA methylomes at base resolution show widespread epigenomic differences. Nature.

[7] Ramsahoye BH, Biniszkiewicz D, Lyko F, Clark V, Bird AP, Jaenisch R (2000). Non-CpG methylation is prevalent in embryonic stem cells and may be mediated by DNA methyltransferase 3a. Proc Natl Acad Sci U S A.

[8] Suzuki MM, Bird A (2008). DNA methylation landscapes: provocative insights from epigenomics. Nat Rev Genet.

[9] Varley KE, Gertz J, Bowling KM, Parker SL, Reddy TE, Pauli-Behn F (2013). Dynamic DNA methylation across diverse human cell lines and tissues. Genome Res.

[10] Zhang X, Yazaki J, Sundaresan A, Cokus S, Chan SWL, Chen H (2006). Genome-wide high-resolution mapping and functional analysis of DNA methylation in Arabidopsis. Cell.

[11] Johnson LM, Bostick M, Zhang X, Kraft E, Henderson I, Callis J (2007). The SRA methyl-cytosine-binding domain links DNA and histone methylation. Curr Biol.

[12] Cao X, Jacobsen SE (2002). Role of the Arabidopsis DRM methyltransferases in de novo DNA methylation and gene silencing. Curr Biol.

[13] Law JA, Jacobsen SE (2010). Establishing, maintaining and modifying DNA methylation patterns in plants and animals. Nat Rev Genet.

[14] Cuerda-Gil D, Slotkin RK (2016). Non-canonical RNA-directed DNA methylation. Nat Plants.

[15] Wassenegger M, Heimes S, Riedel L, Sänger HL (1994). RNA-directed de novo methylation of genomic sequences in plants. Cell.

[16] Huettel B, Kanno T, Daxinger L, Bucher E, van der Winden J, Matzke AJM (2007). RNA-directed DNA methylation mediated by DRD1 and Pol IVb: a versatile pathway for transcriptional gene silencing in plants. Biochim Biophys Acta Gene Struct Express.

[17] Pikaard CS, Haag JR, Ream T, Wierzbicki AT (2008). Roles of RNA polymerase IV in gene silencing. Trends Plant Sci.

[18] Law JA, Ausin I, Johnson LM, Vashisht AA, Zhu JK, Wohlschlegel JA (2010). A protein complex required for polymerase V transcripts and RNA-directed DNA methylation in plants. Curr Biol.

[19] Finnegan EJ, Peacock WJ, Dennis ES (1996). Reduced DNA methylation in Arabidopsis thaliana results in abnormal plant development. Proc Natl Acad Sci U S A.

[20] Kankel MW, Ramsey DE, Stokes TL, Flowers SK, Haag JR, Jeddeloh JA (2003). Arabidopsis MET1 cytosine methyltransferase mutants. Genetics.

[21] Ebbs ML, Bender J (2006). Locus-specific control of DNA methylation by the Arabidopsis SUVH5 histone methyltransferase. Plant Cell.

[22] Henderson IR, Jacobsen SE (2007). Epigenetic inheritance in plants. Nature.

[23] Du J, Zhong X, Bernatavichute YV, Stroud H, Feng S, Caro E (2012). Dual binding of chromomethylase domains to H3K9me2-containing nucleosomes directs DNA methylation in plants. Cell.

[24] Stroud H, Do T, Du J, Zhong X, Feng S, Johnson L (2014). Non-CG methylation patterns shape the epigenetic landscape in Arabidopsis. Nat Struct Mol Biol.

[25] Stroud H, Greenberg MVC, Feng S, Bernatavichute YV, Jacobsen SE (2013). Comprehensive analysis of silencing mutants reveals complex regulation of the Arabidopsis methylome. Cell.

[26] Zemach A, Kim MY, Hsieh PH, Coleman-Derr D, Eshed-Williams L, Thao K (2013). The nucleosome remodeler DDM1 allows DNA methyltransferases to access H1-containing heterochromatin. Cell.

[27] Woo HR, Pontes O, Pikaard CS, Richards EJ (2007). VIM1, a methylcytosine-binding protein required for centromeric heterochromatinization. Genes Dev.

[28] Woo HR, Dittmer TA, Richards EJ. Three SRA-domain methylcytosine-binding proteins cooperate to maintain global CpG methylation and epigenetic silencing in Arabidopsis. Kakutani T, editor. PLoS Genet. 2008;4(8) Available from: https://dx.plos.org/10.1371/journal.pgen.1000156. Cited 2020 Apr 22. 10.1371/journal.pgen.1000156 PMC2491724 18704160

[29] Bostick M, Jong KK, Estève PO, Clark A, Pradhan S, Jacobsen SE (2007). UHRF1 plays a role in maintaining DNA methylation in mammalian cells. Science (80- ).

[30] Sharif J, Muto M, Takebayashi SI, Suetake I, Iwamatsu A, Endo TA (2007). The SRA protein Np95 mediates epigenetic inheritance by recruiting Dnmt1 to methylated DNA. Nature.

[31] Kraft E, Bostick M, Jacobsen SE, Callis J (2008). ORTH/VIM proteins that regulate DNA methylation are functional ubiquitin E3 ligases. Plant J.

[32] Bronner C, Alhosin M, Hamiche A, Mousli M (2019). Coordinated dialogue between UHRF1 and DNMT1 to ensure faithful inheritance of methylated DNA patterns. Genes.

[33] Xue B, Zhao J, Feng P, Xing J, Wu H, Li Y (2019). Epigenetic mechanism and target therapy of uhrf1 protein complex in malignancies. Onco Targets Ther.

[34] Taniue K, Kurimoto A, Sugimasa H, Nasu E, Takeda Y, Iwasaki K (2016). Long noncoding RNA UPAT promotes colon tumorigenesis by inhibiting degradation of UHRF1. Proc Natl Acad Sci U S A.

[35] Li H, Ilin S, Wang W, Duncan EM, Wysocka J, Allis CD (2006). Molecular basis for site-specific read-out of histone H3K4me3 by the BPTF PHD finger of NURF. Nature.

[36] Peña PV, Davrazou F, Shi X, Walter KL, Verkhusha VV, Gozani O (2006). Molecular mechanism of histone H3K4me3 recognition by plant homeodomain of ING2. Nature.

[37] Shi X, Hong T, Walter KL, Ewalt M, Michishita E, Hung T (2006). ING2 PHD domain links histone H3 lysine 4 methylation to active gene repression. Nature.

[38] Wysocka J, Swigut T, Xiao H, Milne TA, Kwon SY, Landry J (2006). A PHD finger of NURF couples histone H3 lysine 4 trimethylation with chromatin remodelling. Nature.

[39] Unoki M, Nishidate T, Nakamura Y (2004). ICBP90, an E2F-1 target, recruits HDAC1 and binds to methyl-CpG through its SRA domain. Oncogene.

[40] Shook MS, Richards EJ (2014). VIM proteins regulate transcription exclusively through the MET1 cytosine methylation pathway. Epigenetics.

[41] Soppe WJJ, Jasencakova Z, Houben A, Kakutani T, Meister A, Huang MS (2002). DNA methylation controls histone H3 lysine 9 methylation and heterochromatin assembly in Arabidopsis. EMBO J.

[42] Tariq M, Saze H, Probst AV, Lichota J, Habu Y, Paszkowski J (2003). Erasure of CpG methylation in Arabidopsis alters patterns of histone H3 methylation in heterochromatin. Proc Natl Acad Sci U S A.

[43] Deleris A, Stroud H, Bernatavichute Y, Johnson E, Klein G, Schubert D (2012). Loss of the DNA methyltransferase MET1 induces H3K9 hypermethylation at PcG target genes and redistribution of H3K27 trimethylation to transposons in Arabidopsis thaliana. PLoS Genet.

[44] Kim J, Kim JH, Richards EJ, Chung KM, Woo HR (2014). Arabidopsis VIM proteins regulate epigenetic silencing by modulating DNA methylation and histone modification in cooperation with MET1. Mol Plant.

[45] Mathieu O, Probst AV, Paszkowski J (2005). Distinct regulation of histone H3 methylation at lysines 27 and 9 by CpG methylation in Arabidopsis. EMBO J.

[46] Gaudin V, Libault M, Pouteau S, Juul T, Zhao G, Lefebvre D (2001). Mutations in LIKE HETEROCHROMATIN PROTEIN 1 affect flowering time and plant architecture in Arabidopsis. Development.

[47] Hsieh TF, Hakim O, Ohad N, Fischer RL (2003). From flour to flower: how Polycomb group proteins influence multiple aspects of plant development. Trends Plant Sci.

[48] Turck F, Roudier F, Farrona S, Martin-Magniette M-L, Guillaume E, Buisine N (2007). Arabidopsis TFL2/LHP1 specifically associates with genes marked by trimethylation of histone H3 lysine 27. PLoS Genet.

[49] Veluchamy A, Jégu T, Ariel F, Latrasse D, Mariappan KG, Kim SK (2016). LHP1 Regulates H3K27me3 spreading and shapes the three-dimensional conformation of the Arabidopsis genome. PLoS One.

[50] Berry S, Rosa S, Howard M, Bühler M, Dean C (2017). Disruption of an RNA-binding hinge region abolishes LHP1-mediated epigenetic repression. Genes Dev.

[51] Ariel F, Jegu T, Latrasse D, Romero-Barrios N, Christ A, Benhamed M (2014). Noncoding transcription by alternative rna polymerases dynamically regulates an auxin-driven chromatin loop. Mol Cell.

[52] Ariel F, Lucero L, Christ A, Mammarella MF, Jegu T, Veluchamy A (2020). R-Loop Mediated trans action of the APOLO long noncoding RNA. Mol Cell.

[53] Moison M, Pacheco JM, Lucero L, Fonouni-Farde C, Rodríguez-Melo J, Mansilla N (2021). The lncRNA APOLO interacts with the transcription factor WRKY42 to trigger root hair cell expansion in response to cold. Mol Plant.

[54] Ge SX, Jung D, Yao R (2020). ShinyGO: a graphical gene-set enrichment tool for animals and plants. Bioinformatics.

[55] Winter D, Vinegar B, Nahal H, Ammar R, Wilson GV, Provart NJ (2007). An “electronic fluorescent pictograph” browser for exploring and analyzing large-scale biological data sets. Baxter I, editor. PLoS One.

[56] Parent B, Tardieu F (2012). Temperature responses of developmental processes have not been affected by breeding in different ecological areas for 17 crop species. New Phytol.

[57] Franklin KA, Lee SH, Patel D, Kumar SV, Spartz AK, Gu C (2011). PHYTOCHROME-INTERACTING FACTOR 4 (PIF4) regulates auxin biosynthesis at high temperature. Proc Natl Acad Sci U S A.

[58] Mashiguchi K, Tanaka K, Sakai T, Sugawara S, Kawaide H, Natsume M (2011). The main auxin biosynthesis pathway in Arabidopsis. Proc Natl Acad Sci U S A.

[59] Sakata T, Oshino T, Miura S, Tomabechi M, Tsunaga Y, Higashitani N (2010). Auxins reverse plant male sterility caused by high temperatures. Proc Natl Acad Sci U S A.

[60] Cheng Y, Dai X, Zhao Y (2006). Auxin biosynthesis by the YUCCA flavin monooxygenases controls the formation of floral organs and vascular tissues in Arabidopsis. Genes Dev.

[61] Xu W, Xu H, Li K, Fan Y, Liu Y, Yang X (2017). The R-loop is a common chromatin feature of the Arabidopsis genome. Nat Plants.

[62] Johnson LM, Du J, Hale CJ, Bischof S, Feng S, Chodavarapu RK (2014). SRA/SET domain-containing proteins link RNA polymerase V occupancy to DNA methylation. Nature.

[63] Gyula P, Baksa I, Tóth T, Mohorianu I, Dalmay T, Szittya G (2018). Ambient temperature regulates the expression of a small set of sRNAs influencing plant development through NF-YA2 and YUC2. Plant Cell Environ.

[64] Feng S, Cokus SJ, Zhang X, Chen PY, Bostick M, Goll MG (2010). Conservation and divergence of methylation patterning in plants and animals. Proc Natl Acad Sci U S A.

[65] Fang J, Cheng J, Wang J, Zhang Q, Liu M, Gong R (2016). Hemi-methylated DNA opens a closed conformation of UHRF1 to facilitate its histone recognition. Nat Commun.

[66] Wang H, Cao D, Wu F (2018). Long noncoding RNA UPAT promoted cell proliferation via increasing UHRF1 expression in non-small cell lung cancer. Oncol Lett.

[67] Gruber AR, Lorenz R, Bernhart SH, Neuböck R, Hofacker IL (2008). The Vienna RNA websuite. Nucleic Acids Res.

[68] Lorenz R, Bernhart SH, Höner zu Siederdissen C, Tafer H, Flamm C, Stadler PF, et al. ViennaRNA Package 2.0. Algorithms Mol Biol. 2011;6(1) Available from: https://pubmed.ncbi.nlm.nih.gov/22115189/. Cited 2022 Apr 17. 10.1186/1748-7188-6-26 PMC3319429 22115189

[69] Rigo R, Bazin J, Romero-Barrios N, Moison M, Lucero L, Christ A (2020). The Arabidopsis lnc RNA ASCO modulates the transcriptome through interaction with splicing factors. EMBO Rep.

[70] Bardou F, Ariel F, Simpson CG, Romero-Barrios N, Laporte P, Balzergue S (2014). Long noncoding RNA modulates alternative splicing regulators in Arabidopsis. Dev Cell.

[71] Gray WM, Östin A, Sandberg G, Romano CP, Estelle M (1998). High temperature promotes auxin-mediated hypocotyl elongation in Arabidopsis. Proc Natl Acad Sci U S A.

[72] Casal JJ, Balasubramanian S (2019). Thermomorphogenesis. Annu Rev Plant Biol.

[73] Bellstaedt J, Trenner J, Lippmann R, Poeschl Y, Zhang X, Friml J (2019). A mobile auxin signal connects temperature sensing in cotyledons with growth responses in hypocotyls. Plant Physiol.

[74] Kumar SV, Wigge PA (2010). H2A.Z-containing nucleosomes mediate the thermosensory response in Arabidopsis. Cell.

[75] Cortijo S, Charoensawan V, Brestovitsky A, Buning R, Ravarani C, Rhodes D (2017). Transcriptional regulation of the ambient temperature response by H2A.Z nucleosomes and HSF1 transcription factors in Arabidopsis. Mol Plant.

[76] Pajoro A, Severing E, Angenent GC, Immink RGH (2017). Histone H3 lysine 36 methylation affects temperature-induced alternative splicing and flowering in plants. Genome Biol.

[77] Steffen A, Staiger D (2017). Chromatin marks and ambient temperature-dependent flowering strike up a novel liaison. Genome Biol.

[78] Susila H, Nasim Z, Ahn JH (2018). Ambient temperature-responsive mechanisms coordinate regulation of flowering time. Int J Mol Sci.

[79] Tasset C, Singh Yadav A, Sureshkumar S, Singh R, van der Woude L, Nekrasov M (2018). POWERDRESS-mediated histone deacetylation is essential for thermomorphogenesis in Arabidopsis thaliana. Queitsch C, editor. PLoS Genet.

[80] Dubin MJ, Zhang P, Meng D, Remigereau MS, Osborne EJ, Casale FP (2015). DNA methylation in Arabidopsis has a genetic basis and shows evidence of local adaptation. Elife.

[81] Kawakatsu T, Huang S, Shan C, Jupe F, Sasaki E, RJJ S, MAA U (2016). Epigenomic diversity in a global collection of Arabidopsis thaliana accessions. Cell.

[82] Shen X, De Jonge J, Forsberg SKG, Pettersson ME, Sheng Z, Hennig L (2014). Natural CMT2 variation is associated with genome-wide methylation changes and temperature seasonality. PLoS Genet.

[83] Popova OV, Dinh HQ, Aufsatz W, Jonak C (2013). The RdDM pathway is required for basal heat tolerance in Arabidopsis. Mol Plant.

[84] Naydenov M, Baev V, Apostolova E, Gospodinova N, Sablok G, Gozmanova M (2015). High-temperature effect on genes engaged in DNA methylation and affected by DNA methylation in Arabidopsis. Plant Physiol Biochem.

[85] Yu Y, Dong A, Shen WH (2004). Molecular characterization of the tobacco SET domain protein NtSET1 unravels its role in histone methylation, chromatin binding, and segregation. Plant J.

[86] Liu S, Yu Y, Ruan Y, Meyer D, Wolff M, Xu L (2007). Plant SET- and RING-associated domain proteins in heterochromatinization. Plant J.

[87] Sidaway-Lee K, Costa MJ, Rand DA, Finkenstadt B, Penfield S (2014). Direct measurement of transcription rates reveals multiple mechanisms for configuration of the Arabidopsis ambient temperature response. Genome Biol.

[88] Rizzardi K, Landberg K, Nilsson L, Ljung K, Sundãs-Larsson A (2011). TFL2/LHP1 is involved in auxin biosynthesis through positive regulation of YUCCA genes. Plant J.

[89] Chung BYW, Balcerowicz M, Di Antonio M, Jaeger KE, Geng F, Franaszek K (2020). An RNA thermoswitch regulates daytime growth in Arabidopsis. Nat Plants.

[90] Fazzio TG (2016). Regulation of chromatin structure and cell fate by R-loops. Transcription.

[91] Crossley MP, Bocek M, Cimprich KA (2019). R-loops as cellular regulators and genomic threats. Mol Cell.

[92] Luo H, Zhu G, Eshelman MA, Fung TK, Lai Q, Wang F (2022). HOTTIP-dependent R-loop formation regulates CTCF boundary activity and TAD integrity in leukemia. Mol Cell.

[93] Chalei V, Sansom SN, Kong L, Lee S, Montiel JF, Vance KW (2014). The long non-coding RNA Dali is an epigenetic regulator of neural differentiation. Elife.

[94] Arab K, Park YJ, Lindroth AM, Schäfer A, Oakes C, Weichenhan D (2014). Long noncoding RNA TARID directs demethylation and activation of the tumor suppressor TCF21 via GADD45A. Mol Cell.

[95] Arab K, Karaulanov E, Musheev M, Trnka P, Schäfer A, Grummt I (2019). GADD45A binds R-loops and recruits TET1 to CpG island promoters. Nat Genet.

[96] Mohammad F, Mondal T, Guseva N, Pandey GK, Kanduri C (2010). Kcnq1ot1 noncoding RNA mediates transcriptional gene silencing by interacting with Dnmt1. Development.

[97] Di Ruscio A, Ebralidze AK, Benoukraf T, Amabile G, Goff LA, Terragni J (2013). DNMT1-interacting RNAs block gene-specific DNA methylation. Nature.

[98] Ginno PA, Lott PL, Christensen HC, Korf I, Chédin F (2012). R-loop formation is a distinctive characteristic of unmethylated human CpG island promoters. Mol Cell.

[99] Grunseich C, Wang IX, Watts JA, Burdick JT, Guber RD, Zhu Z (2018). Senataxin mutation reveals how R-loops promote transcription by blocking DNA methylation at gene promoters. Mol Cell.

[100] Skourti-Stathaki K, Torlai Triglia E, Warburton M, Voigt P, Bird A, Pombo A. R-loops enhance polycomb repression at a subset of developmental regulator genes. Mol Cell. 2019;73(5):930-945.e4. Available from: 10.1016/j.molcel.2018.12.016 10.1016/j.molcel.2018.12.016 PMC6414425 30709709

[101] Smallwood A, Estève PO, Pradhan S, Carey M (2007). Functional cooperation between HP1 and DNMT1 mediates gene silencing. Genes Dev.

[102] Nady N, Lemak A, Walker JR, Avvakumov GV, Kareta MS, Achour M (2011). Recognition of multivalent histone states associated with heterochromatin by UHRF1 protein. J Biol Chem.

[103] Rothbart SB, Krajewski K, Nady N, Tempel W, Xue S, Badeaux AI (2012). Association of UHRF1 with H3K9 methylation directs the maintenance of DNA methylation. Nat Struct Mol Biol.

[104] Nishiyama A, Yamaguchi L, Sharif J, Johmura Y, Kawamura T, Nakanishi K (2013). Uhrf1-dependent H3K23 ubiquitylation couples maintenance DNA methylation and replication. Nature..

[105] Bashtrykov P, Jankevicius G, Jurkowska RZ, Ragozin S, Jeltsch A (2014). The UHRF1 protein stimulates the activity and specificity of the maintenance DNA methyltransferase DNMT1 by an allosteric mechanism. J Biol Chem.

[106] Zhao Q, Zhang J, Chen R, Wang L, Li B, Cheng H (2016). Dissecting the precise role of H3K9 methylation in crosstalk with DNA maintenance methylation in mammals. Nat Commun.

[107] Rose NR, Klose RJ (2014). Understanding the relationship between DNA methylation and histone lysine methylation. Biochim Biophys Acta Gene Regul Mech.

[108] Yao Y, Bilichak A, Golubov A, Kovalchuk I (2012). ddm1 plants are sensitive to methyl methane sulfonate and NaCl stresses and are deficient in DNA repair. Plant Cell Rep.

[109] Piacentini L, Fanti L, Negri R, Del Vescovo V, Fatica A, Altieri F (2009). Heterochromatin Protein 1 (HP1a) positively regulates euchromatic gene expression through RNA transcript association and interaction with hnRNPs in Drosophila. PLoS Genet.

[110] Shuai T, Khan MR, Zhang XD, Li J, Thorne RF, Wu M (2021). lncRNA TRMP-S directs dual mechanisms to regulate p27-mediated cellular senescence. Mol Ther - Nucleic Acids.

[111] Roulé T, Christ A, Hussain N, Huang Y, Hartmann C, Benhamed M, et al. The lncRNA MARS modulates the epigenetic reprogramming of the marneral cluster in response to ABA. Mol Plant. 2022; Available from: https://pubmed.ncbi.nlm.nih.gov/35150931/. Cited 2022 Apr 17. 10.1016/j.molp.2022.02.007 35150931

[112] Antunez-Sanchez J, Naish M, Ramirez-Prado JS, Ohno S, Huang Y, Dawson A (2020). A new role for histone demethylases in the maintenance of plant genome integrity. Elife.

[113] Déléris A, Berger F, Duharcourt S. Role of Polycomb in the control of transposable elements. Trends Genet. 2021:1–8. 10.1016/j.tig.2021.06.003. 10.1016/j.tig.2021.06.003 34210514

[114] Wang Y, Fan X, Lin F, He G, Terzaghi W, Zhu D (2014). Arabidopsis noncoding RNA mediates control of photomorphogenesis by red light. Proc Natl Acad Sci U S A..

[115] Severing E, Faino L, Jamge S, Busscher M, Kuijer-Zhang Y, Bellinazzo F (2018). Arabidopsis thaliana ambient temperature responsive lncRNAs. BMC Plant Biol.

[116] Clough SJ, Bent AF (2008). Floral dip: a simplified method for Agrobacterium-mediated transformation of Arabidopsis thaliana. Plant J.

[117] Lampropoulos A, Sutikovic Z, Wenzl C, Maegele I, Lohmann JU, Forner J (2013). GreenGate - a novel, versatile, and efficient cloning system for plant transgenesis. Janssen PJ, editor. PLoS One.

[118] Fauser F, Schiml S, Puchta H (2014). Both CRISPR/Cas-based nucleases and nickases can be used efficiently for genome engineering in Arabidopsis thaliana. Plant J.

[119] Morineau C, Bellec Y, Tellier F, Gissot L, Kelemen Z, Nogué F (2017). Selective gene dosage by CRISPR-Cas9 genome editing in hexaploid Camelina sativa. Plant Biotechnol J.

[120] Waadt R, Kudla J. In planta visualization of protein interactions using bimolecular fluorescence complementation (BiFC). Cold Spring Harb Protoc. 2008;3(4) Available from: http://www.ncbi.nlm.nih.gov/pubmed/21356813. Cited 2020 Apr 22. 10.1101/pdb.prot4995 21356813

[121] Liu ZW, Zhao N, Su YN, Chen SS, He XJ (2020). Exogenously overexpressed intronic long noncoding RNAs activate host gene expression by affecting histone modification in Arabidopsis. Sci Rep.

[122] Schindelin J, Arganda-Carreras I, Frise E, Kaynig V, Longair M, Pietzsch T (2012). Fiji: an open-source platform for biological-image analysis. Nat Methods.

[123] Chu C, Quinn J, Chang HY. Chromatin isolation by RNA purification (ChIRP). J Vis Exp. 2012;(61):3912. 10.3791/3912 PMC3460573 22472705

[124] Bolger AM, Lohse M, Usadel B (2014). Trimmomatic: a flexible trimmer for Illumina sequence data. Bioinformatics.

[125] Kopylova E, Noé L, Touzet H (2012). SortMeRNA: fast and accurate filtering of ribosomal RNAs in metatranscriptomic data. Bioinformatics.

[126] Cheng C-Y, Krishnakumar V, Chan AP, Thibaud-Nissen F, Schobel S, Town CD (2017). Araport11: a complete reannotation of the *Arabidopsis thaliana* reference genome. Plant J.

[127] Love MI, Huber W, Anders S (2014). Moderated estimation of fold change and dispersion for RNA-seq data with DESeq2. Genome Biol.

[128] Kolde R (2019). pheatmap: Pretty Heatmaps version 1.0.12 from CRAN.

[129] Sorenson R, Bailey-Serres J (2015). Rapid immunopurification of ribonucleoprotein complexes of plants. Methods Mol Biol.

[130] Nagymihály M, Veluchamy A, Györgypál Z, Ariel F, Jégu T, Benhamed M (2017). Ploidy-dependent changes in the epigenome of symbiotic cells correlate with specific patterns of gene expression. Proc Natl Acad Sci U S A.

[131] Fonouni-Farde C, Christ A, Blein T, Ariel F. The lncRNA *APOLO* and the methylcytosine-binding protein *VIM1* are thermomorphogenesis regulators. Dataset GSE167879. Samples of WT 23°C and 29°C, OE *APOLO-1* and *vim1-3*. 2021. https://www.ncbi.nlm.nih.gov/geo/query/acc.cgi?acc=GSE167879.

[132] Fonouni-Farde C, Christ A, Blein T, Ariel F. The Human *UPAT* and the Arabidopsis *APOLO* lncRNA regulated a comparable set of genes in Arabidopsis. Dataset GSE210828. Samples of WT, OE *APOLO-1* and OE *UPAT-1*. 2022. https://www.ncbi.nlm.nih.gov/geo/query/acc.cgi?acc=GSE210828.

[133] Hall TA (1999). BioEdit: a user-friendly biological sequence alignment editor and analysis program for Windows 95/98/NT. Nucl Acids Symp Ser.

