# Supplementary material for: The Arabidopsis APOLO and human UPAT sequence-unrelated long noncoding RNAs can modulate DNA and histone methylation machineries in plants
Source: Genome Biol. 2022 Aug 29;23:181. doi: 10.1186/s13059-022-02750-7 (PMC9422110; doi:10.1186/s13059-022-02750-7)
Supplement: Supplementary file 1 — Additional file 1: Figure S1. GFP-VIM1 localizes at the nucleus. Figure S2. Characterization of Arabidopsis thaliana VIM1 over-expression and vim1 homozygous T-DNA insertion lines. Figure S3. VIM1, VIM2 and VIM3 are differentially regulated in reponse to heat. Figure S4. APOLO and VIM1 regulate hypocotyle elongation in response to heat. Figure S5. Gene categories transcriptionally regulated in thermomorphogenesis. Figure S6. Epigenetic profile of the YUCCA2 locus. Figure S7. de novo DNA methylation and its maintenance regulate thermomorphogenesis. Figure S8. Bimolecular Fluorescence Complementation (BiFC) assay in transiently transformed Nicotiana benthamiana leaves. Figure S9. APOLO and UPAT lncRNAs share mechanisms of interaction with methylcytosine-binding proteins but no sequence similarity [133]. Figure S10. Constitutive expression of the lncRNA UPAT in Arabidopsis seedlings impairs YUCCA2 transcriptional accumulation. Figure S11. CRISPR/Cas9 strategy for APOLO deletion. [file 13059_2022_2750_MOESM1_ESM.pdf]

Additional File 1, Fig. S1

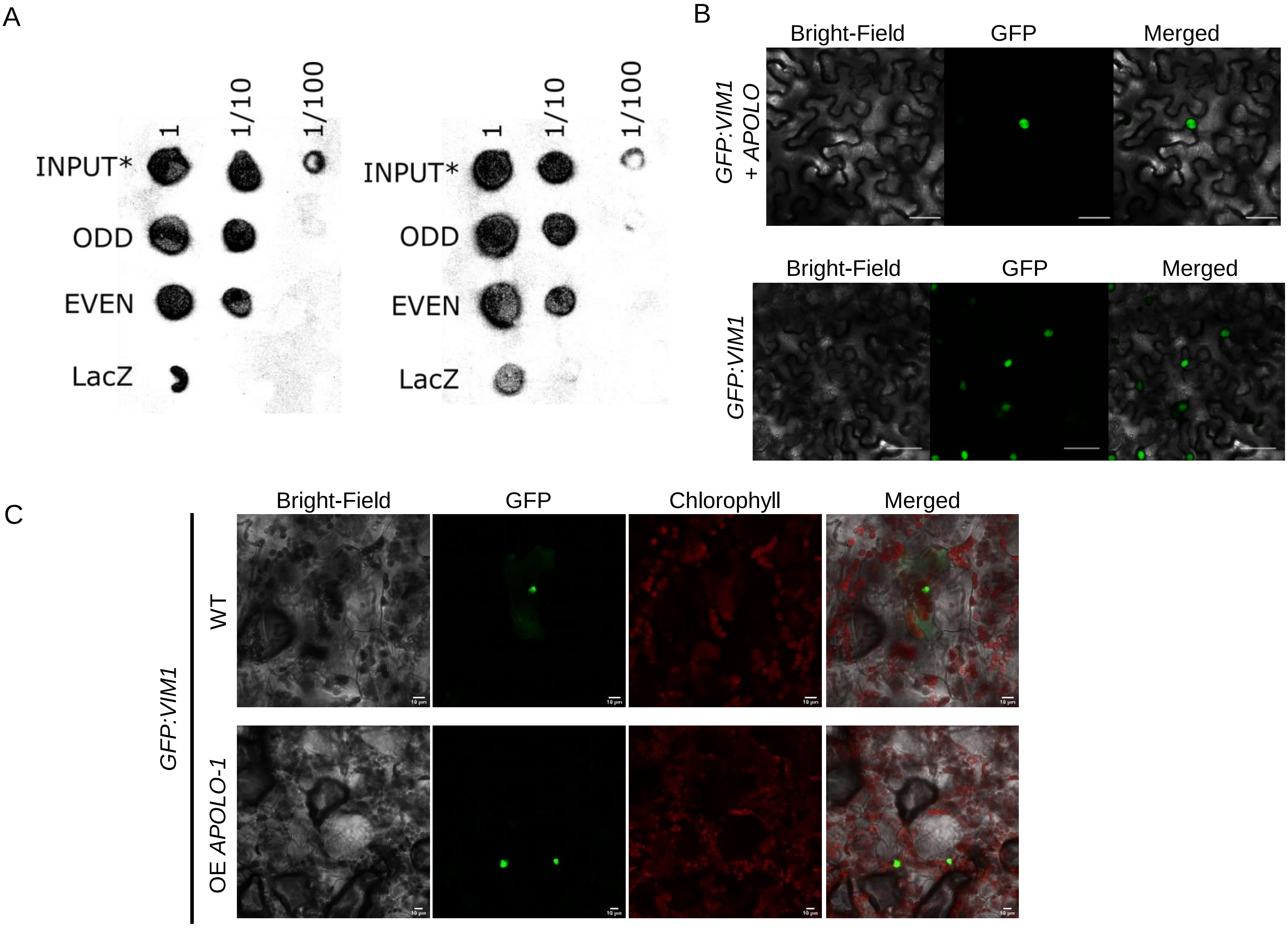

Figure S1: GFP-VIM1 localizes at the nucleus

(**A**) Chromatin isolation by RNA purification (ChIRP)-Dot blot analysis of *APOLO* interaction with GFP-VIM1. ChIRP was performed using ODD and EVEN sets of probes against *APOLO* or using LacZ probes as a negative control. Dot blots are revealed with an anti-GFP antibody and an HRP-conjugated secondary antibody. Diluted INPUT (*1/50) were used as loading control. Two replicates are shown. (**B**) Sub-cellular localization of GFP-VIM1 translational fusion transiently expressed under the control of the 35S-CaMV promoter in *Nicotiana benthamiana* leaves in presence (left panel) or absence (right panel) of *APOLO*. Bright-field image (left), GFP fluorescence alone (middle) and bright-field merged images (right) are shown. Scale bars, 50µm. (**C**) Sub-cellular localization of GFP-VIM1 translational fusion transiently expressed under the control of the 35S-CaMV promoter in *Arabidopsis thaliana* wild-type (WT) and *APOLO* over-expression (OE *APOLO-1*) leaves. Bright-field image (left), GFP or chlorophyll fluorescence alone (middle), and bright-field merged images (right) are shown. Scale bars, 10µm.
In (**B-C**), one representative picture out of three biological replicates is shown.

Additional File 1, Fig. S2

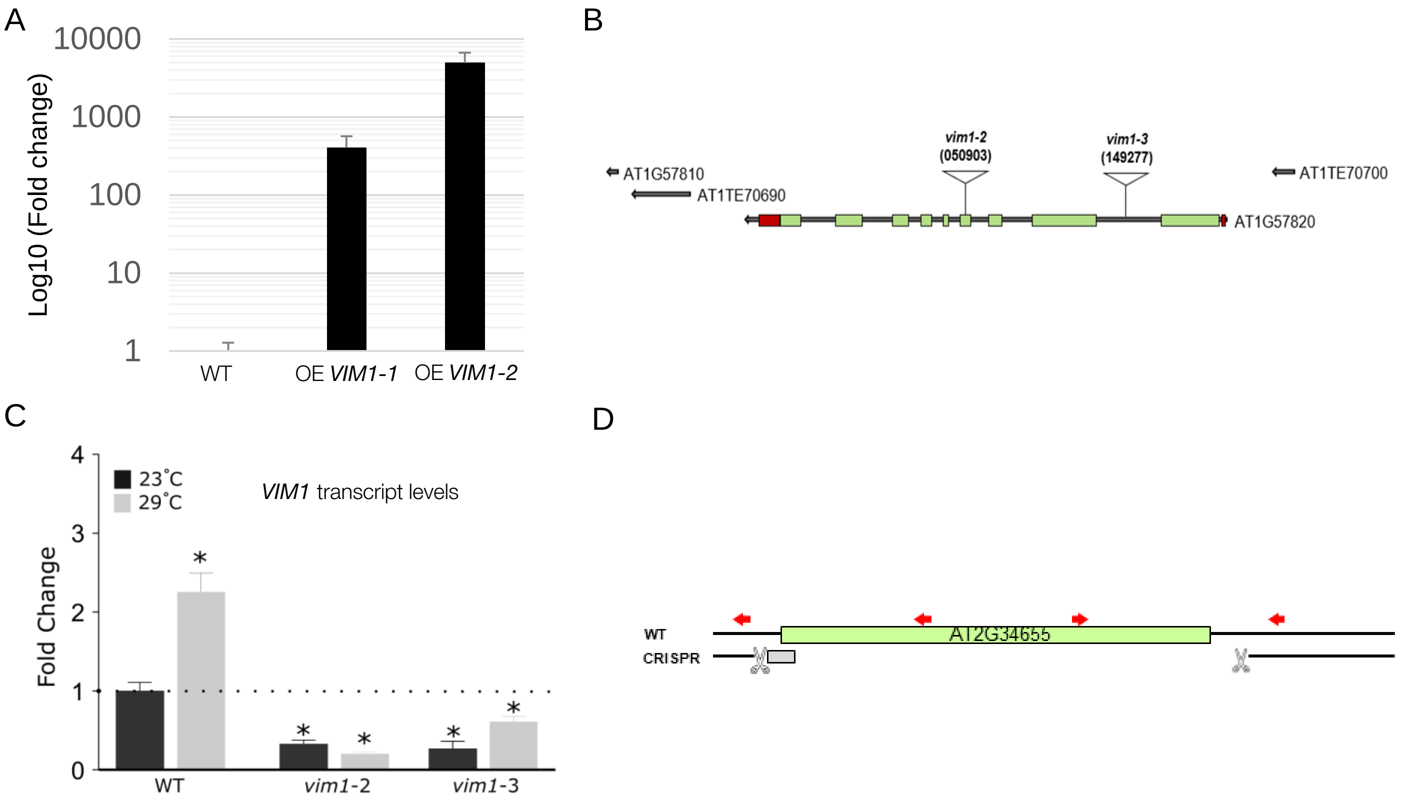

Figure S2: Characterization of *Arabidopsis thaliana VIM1* over-expression and *vim1* homozygous T-DNA insertion lines

(**A**) *VIM1* basal transcript levels in wild-type (WT), *VIM1-1* (OE *VIM1-1*) and *VIM1-2* (OE *VIM1-2*) over-expression lines. (**B**) Schematic representation of the intron-exon structure of the *VIM1* gene (AT1G57820) and positions of T-DNA insertions with allele designations (SALK collection: *vim1-2*: 050903; *vim1-3*: 149277). Exons are indicated by green boxes and introns by grey lines. 5' and 3' UTR regions are indicated by red boxes. AT1G57810 (reverse transcriptase pseudogene), AT1TE70690 and AT1TE70700 (LINE1 transposons) located near AT1G57820 are indicated by grey arrows. (**C**) *VIM1* basal transcript levels in 4-day-old WT, *vim1-2* and *vim1-3* homozygous T-DNA insertion lines at 23°C and after 6h at 29°C. Asterisks indicate Student's t-test ≤ 0.05 (n = 3) between each corresponding genotype/condition and WT at 23°C. (**D**) Schematic representation of the *APOLO* gene (AT2G35655) in WT and CRISPR lines. *APOLO* gene is indicated by a green box and sgRNA guides are represented by red arrows. Deletion borders in the CRISPR/Cas9 *APOLO* line and inserted DNA are respectively indicated by scissors and grey box.

In (**A**), (**C**), transcript levels are normalized to *PP2A* expression levels. Bars represent average ± SD (n = 3 independent pools of seedlings).

Additional File 1, Fig. S3

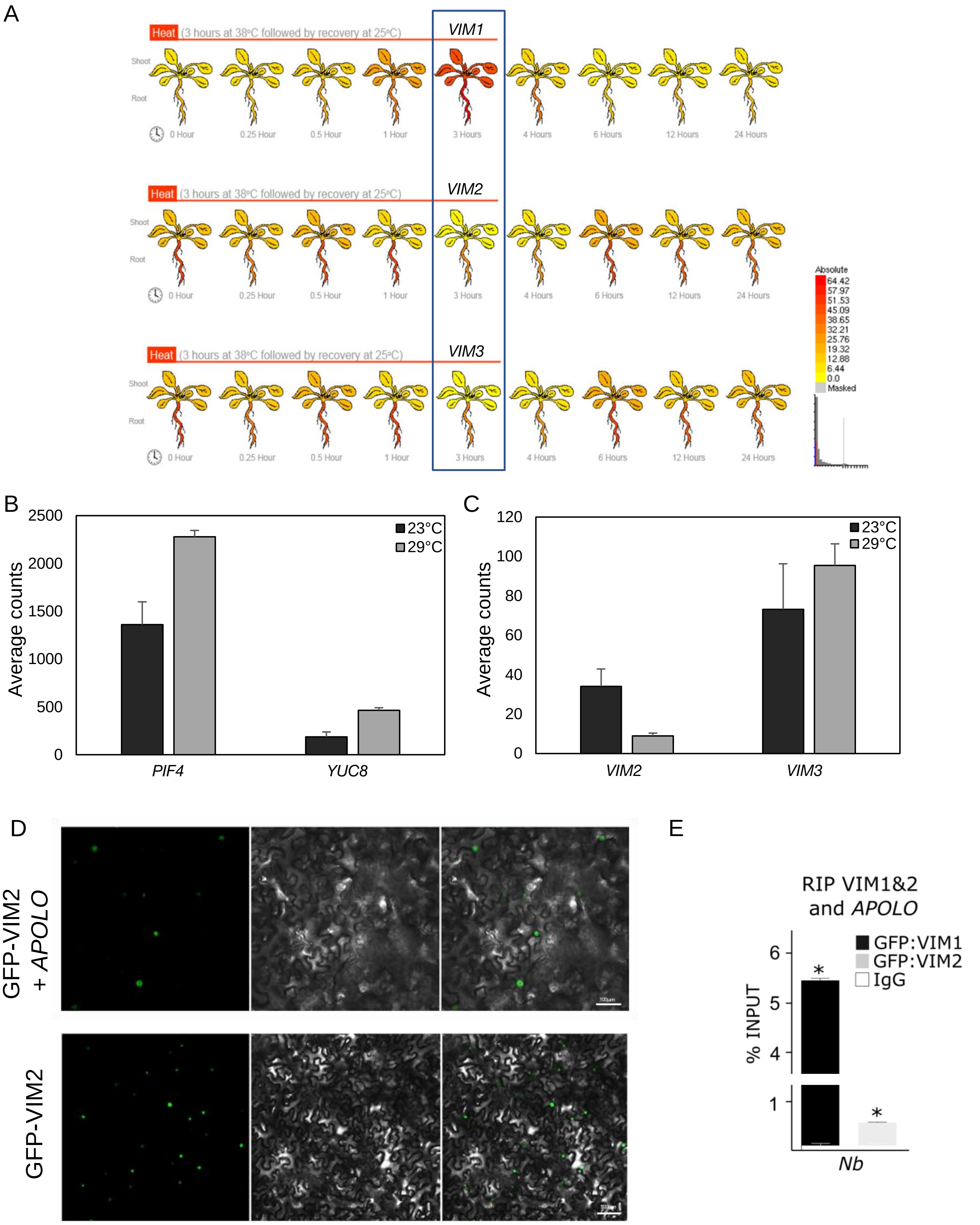

Figure S3: *VIM1*, *VIM2* and *VIM3* are differentially regulated in reponse to heat
(**A**) eFP browser view of *VIM1*, *VIM2* and *VIM3* expression patterns in Arabidopsis shoot and root in response to heat (3 hours at 38°C followed by recovery at 25°C). Expression strength coded by color: yellow = low, red = high. The Arabidopsis eFP Browser is located at bar.utoronto.ca and published in (54). The blue rectangle points at the major difference among genes. (**B-C**) Expression level of the heat markers *PIF4* and *YUC8* (in **B**), and *VIM1* homologs *VIM2* and *VIM3* (in **C**), in 4-day-old wild-type (WT) seedlings treated with heat (29°C) for 6h, related to Figure 1. (**D**) Sub-cellular localization of GFP-VIM2 translational fusion transiently expressed under the control of the 35S-CaMV promoter in *Nicotiana benthamiana* leaves in presence (upper panel) or absence (lower panel) of *APOLO*. GFP fluorescence alone (left), bright-field image (middle) and bright-field merged images (right) are shown. Scale bars, 100 µm. (**E**) RNA immunoprecipitation (RIP) assay in *Nicotiana benthamiana* leaves transiently co-transformed with *APOLO* and *GFP-VIM1* or *GFP-VIM2* translational fusion expressed under the control of the 35S-CaMV promoter. Results are expressed as a percentage of the INPUT fraction. Anti-IgG antibodies were used as a negative control, bars represent average ±SD and the asterisks indicate Student's t-test ≤ 0.05 (n = 3) between anti-GFP and anti-IgG RIPs for each construct.
In (**B-C**), results are expressed as normalized counts obtained by RNA-sequencing. Bars represent average ± SD (n = 3 biological replicates).

Additional File 1, Fig. S4

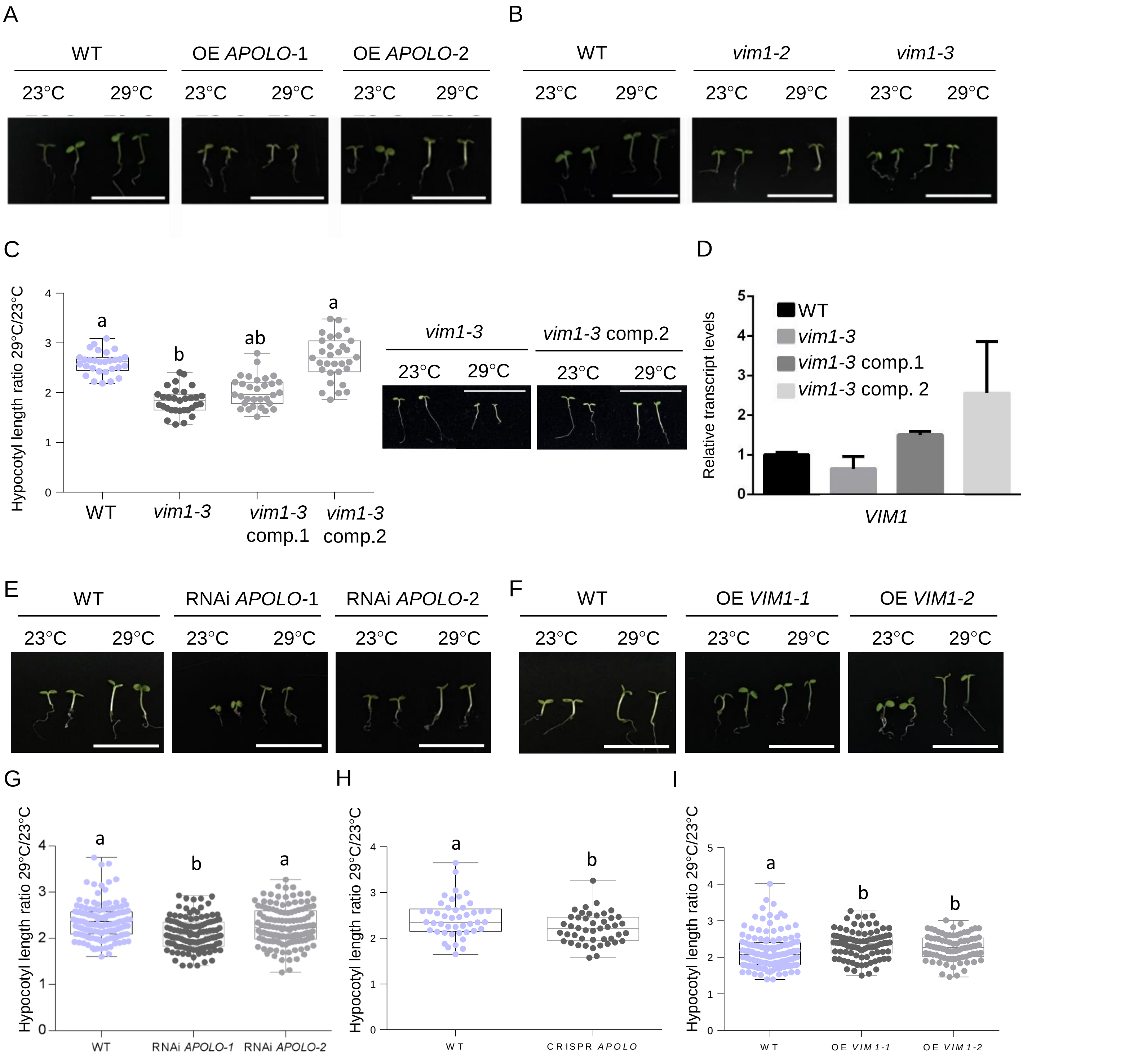

Figure S4: ***APOLO* and VIM1 regulate hypocotyle elongation in response to heat**

(**A-B and E-F**) Representative morphological phenotypes of 4-day-old *APOLO* overexpression (OE *APOLO-1*, OE *APOLO-2*) (**A**), *vim1* mutants (*vim1-2*, *vim1-3*) (**B**), RNAi *APOLO* (RNAi *APOLO*-1, RNAi *APOLO*-2) (**E**) or *VIM1* overexpression (OE *VIM1-1*, OE *VIM1-2*) (**F**) seedlings and their associated wild-type (WT) grown at 23°C or 29°C. Scale bars, 1 cm. (**C** and **G-I**) Boxplots showing hypocotyl length at 29°C over 23°C of 4-day-old *vim1-3* and the corresponding *vim1-3* tranformed with the *proVIM1:GFP:VIM1* construct (**C**), RNAi *APOLO* (**G**), CRISPR *APOLO* (**H**) or *VIM1* over-expression (**I**) seedlings and their associated WT. Values are represented by colored points. Representative morphological phenotypes are shown on the left. (**D**) Transcript levels measured by RT-qPCR of *VIM1* in the plants used in (**C**).
In (**C, G-I**), results are the mean of three biological replicates and letters indicate significant differences compared to WT, based on a Kruskal-Wallis test (α = 0.05; n ≥ 29). In (H), results are the mean of three biological replicates and letters indicate significant differences compared to WT, based on a Mann and Whitney test (α = 0.05; n ≥ 30).

Additional File 1, Fig. S5

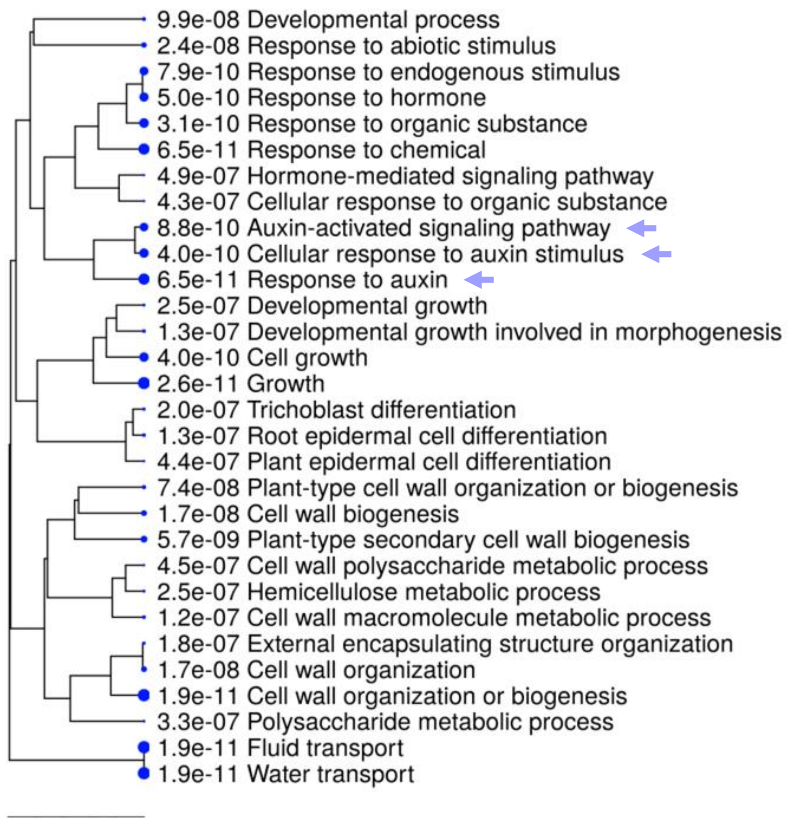

Figure S5: Gene categories transcriptionally regulated in thermomorphogenesis

Gene ontology (GO) enrichment analyses of upregulated transcripts in 4-day-old wild-type (WT) seedlings treated with heat (29°C) for 6h. The hierarchical clustering trees summarize the correlation among significant pathways. Pathways with many shared genes are clustered together. Bigger dots indicate more significant P-values. Arrows indicate auxin-related pathways. The ShinyGO Browser is located at bioinformatics.sdstate.edu and published in (57).

Additional File 1, Fig. S6

A

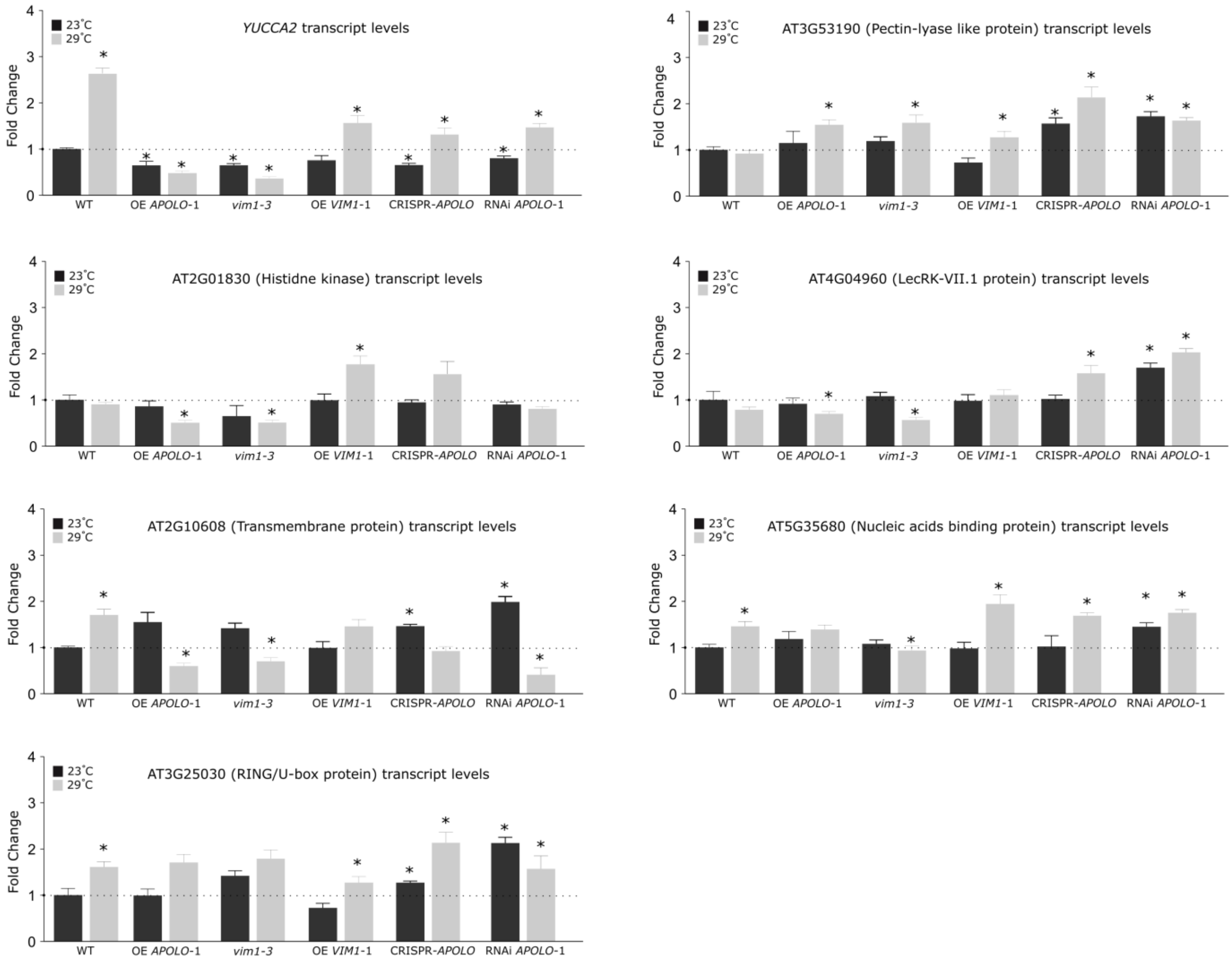

B

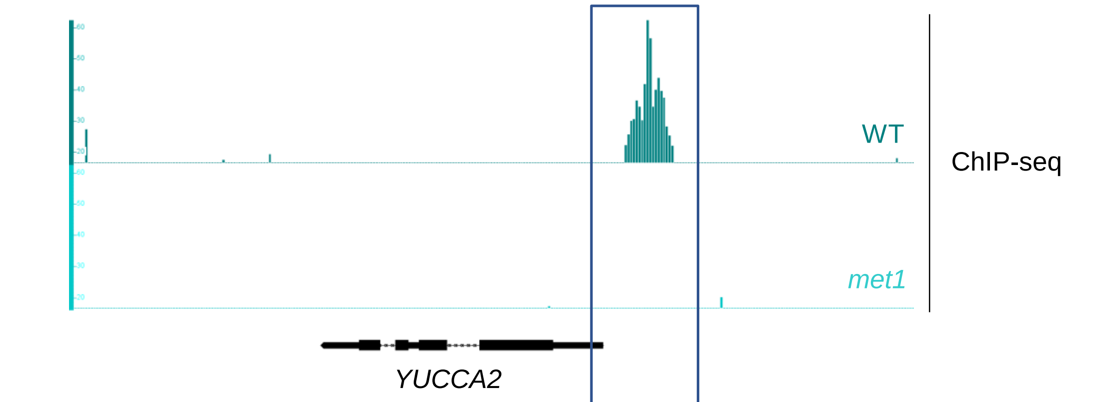

C

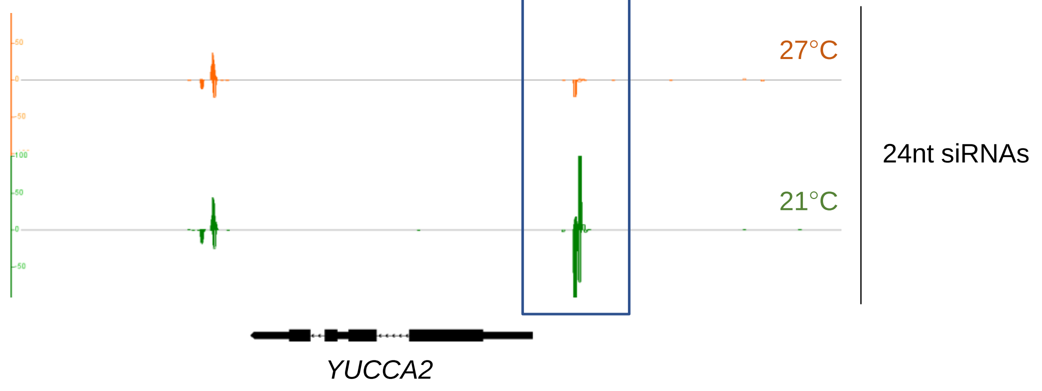

Cont. in next page

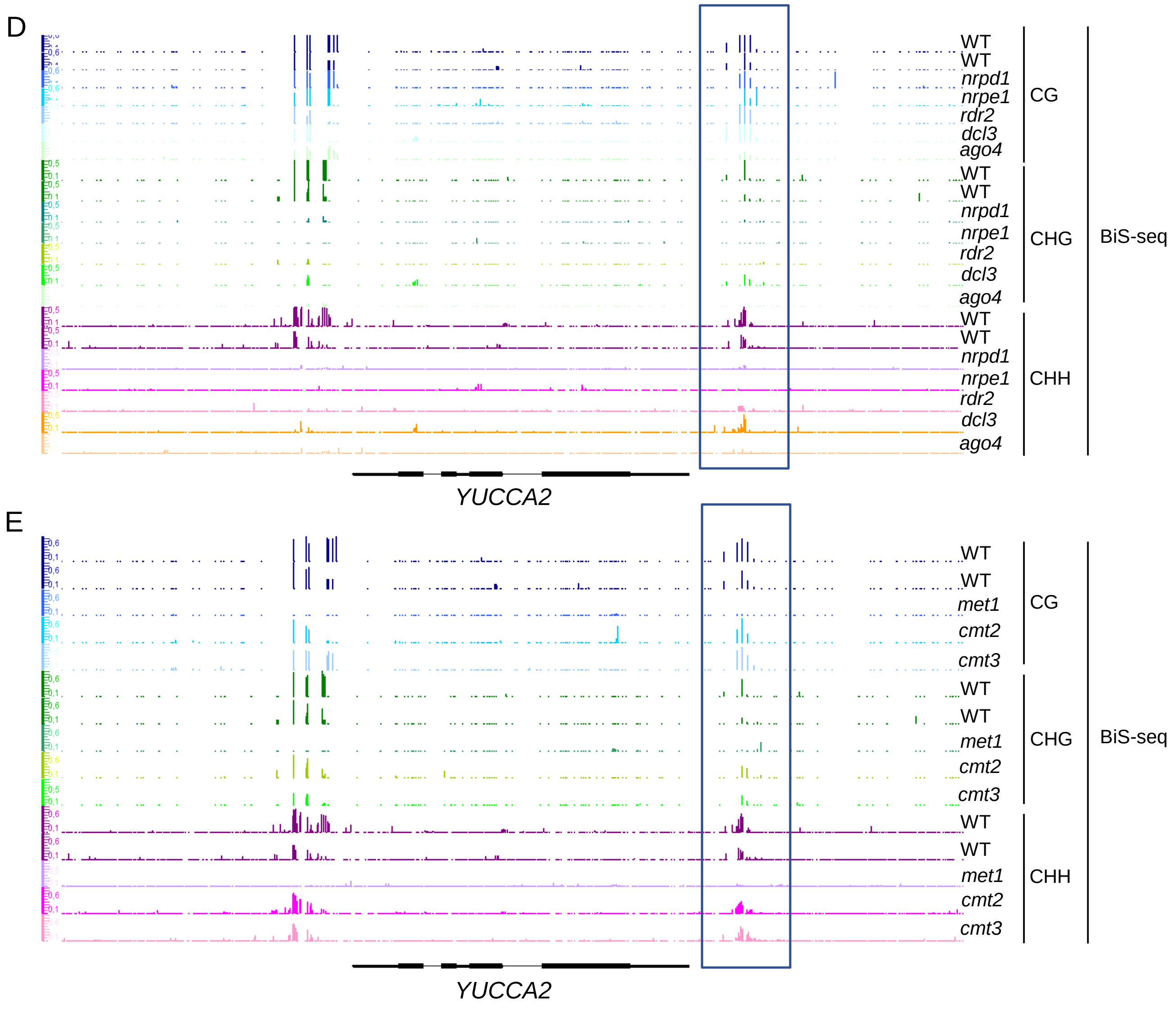

Figure S6: Epigenetic profile of the *YUCCA2* locus

(**A**) Transcript levels of potential common targets of VIM1 and *APOLO* which are deregulated in CHH methylation in *vim1* mutants (Supplementary Table 3) in WT, OE *APOLO*-1, *vim1-3*, OE *VIM1*-1 (*35S:GFP:VIM1* in the WT background), CRISPR-*APOLO*, RNAi *APOLO*-1 lines at 23°C and 29°C. Asterisks indicate Student's t-test ≤ 0.05 (n = 3) between WT at 23°C and 29°C, or between each genotype and WT at the same temperature. Basal transcript levels of AT2G15420, AT1G43730, AT2G12405 were too low for quantification by qPCR (Ct ≥ 35). AT4G05612 was too short for primer design. (**B**) PolV binding at the *YUCCA2* (*YUC2*) locus by NRPE1 subunit-chromatin immunoprecipitation (ChIP)-sequencing in wild-type (WT; Track 1) and *met1* mutant (Track 2) (62). (**C**) 24nt siRNA coverage at the *YUC2* locus at 27°C (Track 1) or 21°C (Track 2) (63). (**D**) Distribution of DNA methylation at the *YUC2* locus by bisulfite (BiS)-sequencing, in the three sequence contexts CG (Tracks 1 to 7), CHG (Tracks 8 to 14) and CHH (Tracks 15 to 21), in *nrpd1* (Tracks 3, 10, 17), *nrpe1* (Tracks 4, 11, 18), *rdr2* (Tracks 5, 12, 19), *ago4* (Tracks 6, 13, 20) or *dcl3* (Tracks 7, 14, 21) mutants and their associated WT (Tracks 1, 2, 8, 9, 15, 16) (25). (**E**) Distribution of DNA methylation at the *YUC2* locus by BiS-seq, in the three sequence contexts CG (Tracks 1 to 5), CHG (Tracks 6 to 10) and CHH (Tracks 11 to 15), in *met1* (Tracks 3, 8, 13), *cmt2* (Tracks 4, 9, 14), or *cmt3* (Tracks 5, 10, 15) mutants and their associated WT (Tracks 1, 2, 6, 7, 11, 12) (25). In (**B-E**), gene annotation is shown at the bottom. The vertical rectangles highlight the same region of interest in all panels.

Additional File 1, Fig. S7

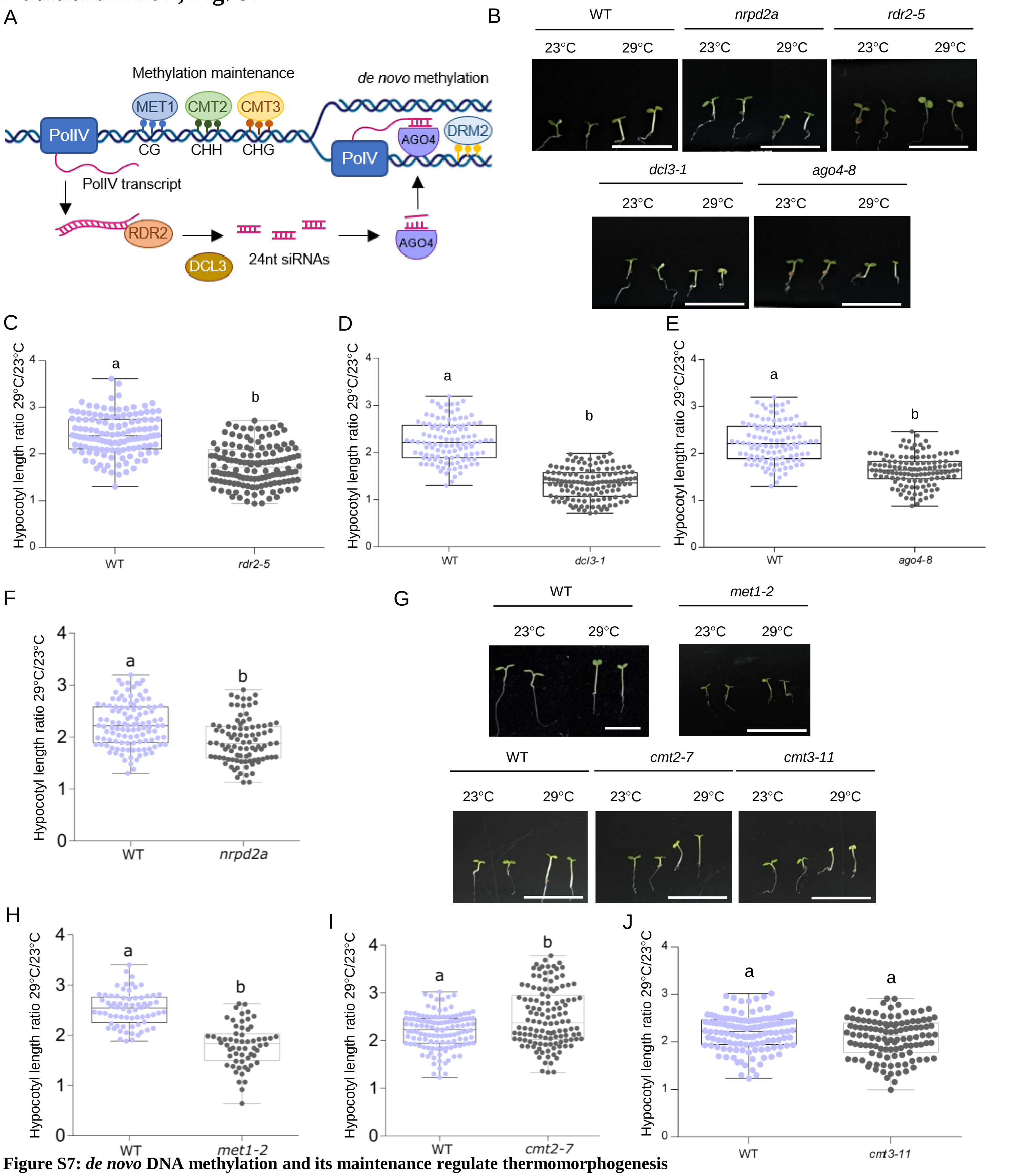

Figure S7: *de novo* DNA methylation and its maintenance regulate thermomorphogenesis

(**A**) Schematic representation of maintenance and *de novo* DNA methylationthe induced by the RNA-directed DNA methylation (RdDM) pathway. AGO4: ARGONAUTE 4; CMT2: CHROMOMETHYLASE 2; CMT3: CHROMOMETHYLASE 3; DCL3: DICER-LIKE 3; DRM2: DOMAINS REARRANGED METHYLTRANSFERASE 2; MET1: METHYLTRANSFERASE 1; PolIV: RNA Polymerase IV; PolV: RNA Polymerase V; RDR2: RNA-DEPENDENT RNA POLYMERASE 2; 24nt siRNA: 24 nucleotide small interfering RNA. CG, CHG and CHH represent the three sequence contexts of DNA methylation. H is A, T, or C. (**B**) Representative morphological phenotypes of 4-day-old RdDM-related mutant seedlings *rdr2-5*, *ago4-8*, *dcl3-1*, *nrpd2a* and their associated wild-type (WT), grown at 23°C or 29°C. Scale bars, 1 cm. (**C-F**) Boxplots showing hypocotyl length at 29°C over 23°C of 4-day-old RdDM-related mutant seedlings *rdr2-5* (**C**), *dcl3-1* (**D**), *ago4-8* (**E**), *nrpd2a* (**F**) and their associated WT. (**G**) Representative morphological phenotypes of 4-day-old methyltransferase mutant *met1-2*, *cmt2-7*, *cmt3-11* seedlings and their associated WT, grown at 23°C or 29°C. Scale bars, 1 cm. (**H-J**) Boxplots showing hypocotyl length at 29°C over 23°C of 4-day-old methyltransferase mutant *met1-2* (**H**), *cmt2-7* (**I**), *cmt3-11* (**J**) seedlings and their associated WT.

In (**C-F**) and (**H-J**), results are the mean of three biological replicates and letters indicate significant differences compared to WT, based on a Kruskal-Wallis test (α = 0.05; n $\geq$ 60).

Additional File 1, Fig. S8

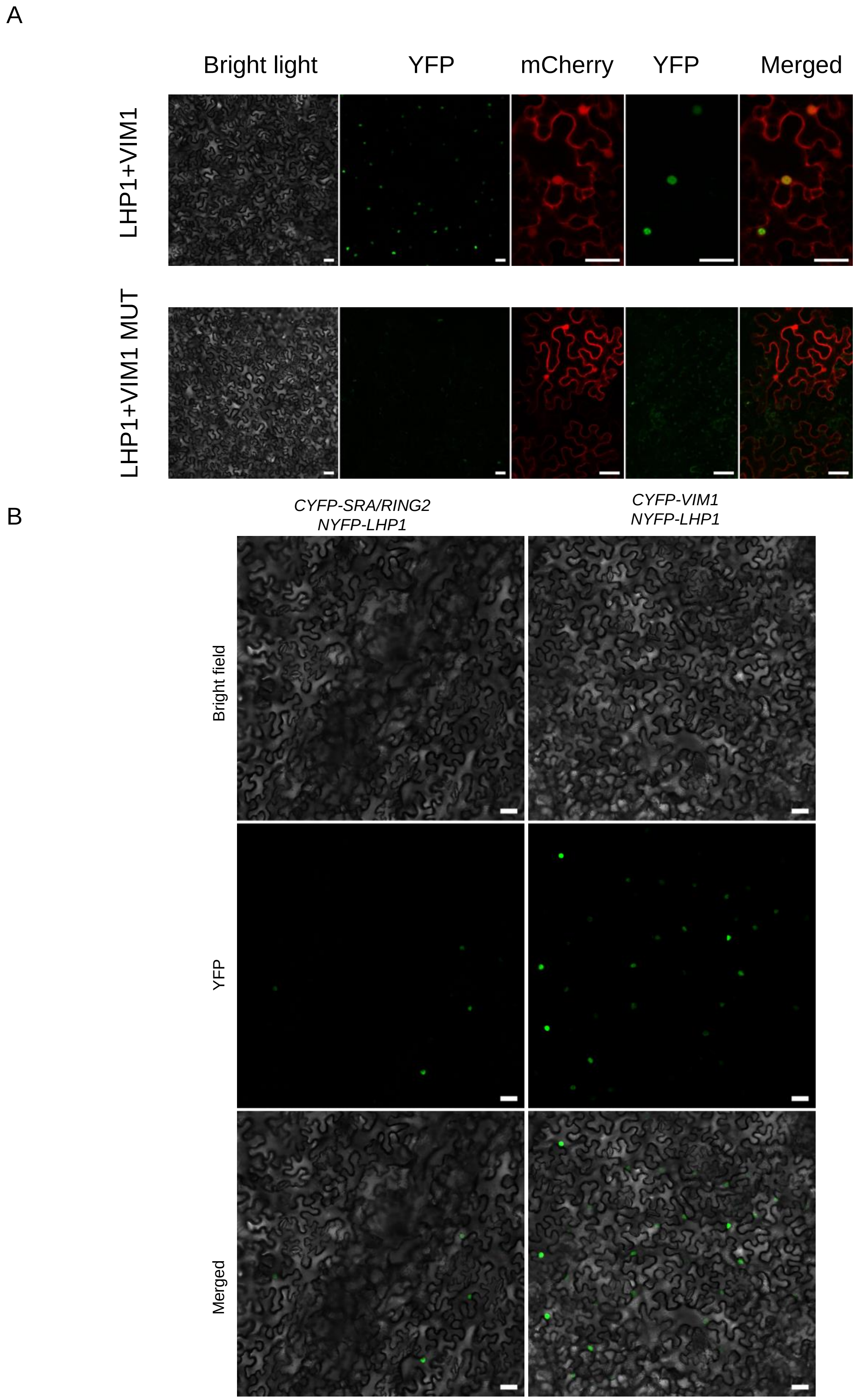

Figure S8: Bimolecular Fluorescence Complementation (BiFC) assay in transiently transformed *Nicotiana benthamiana* leaves

(**A**) BiFC equivalent to Figure 3J, including the transformation with 35S:mCherry for the identification of membranes and nuclei. CYFP was fused to VIM1 or SRA/RING2 and NYFP was fused to LHP1. In both panels, bright-field (left), YFP fluorescence, and zoomed-in mCherry, YFP and merged images (right) are shown. Scale bars, 20μm.

(**B**) CYFP was fused to VIM1 or SRA/RING2 and NYFP was fused to LHP1. Only five nuclei were observed in one out of six biological replicates when CYFP-SRA/RING2 is co-expressed with NYFP-LHP1. In both panels, bright-field images (top), YFP fluorescence alone (middle) and bright-field merged images (bottom) are shown. Scale bars, 50μm.

Additional File 1, Fig. S9

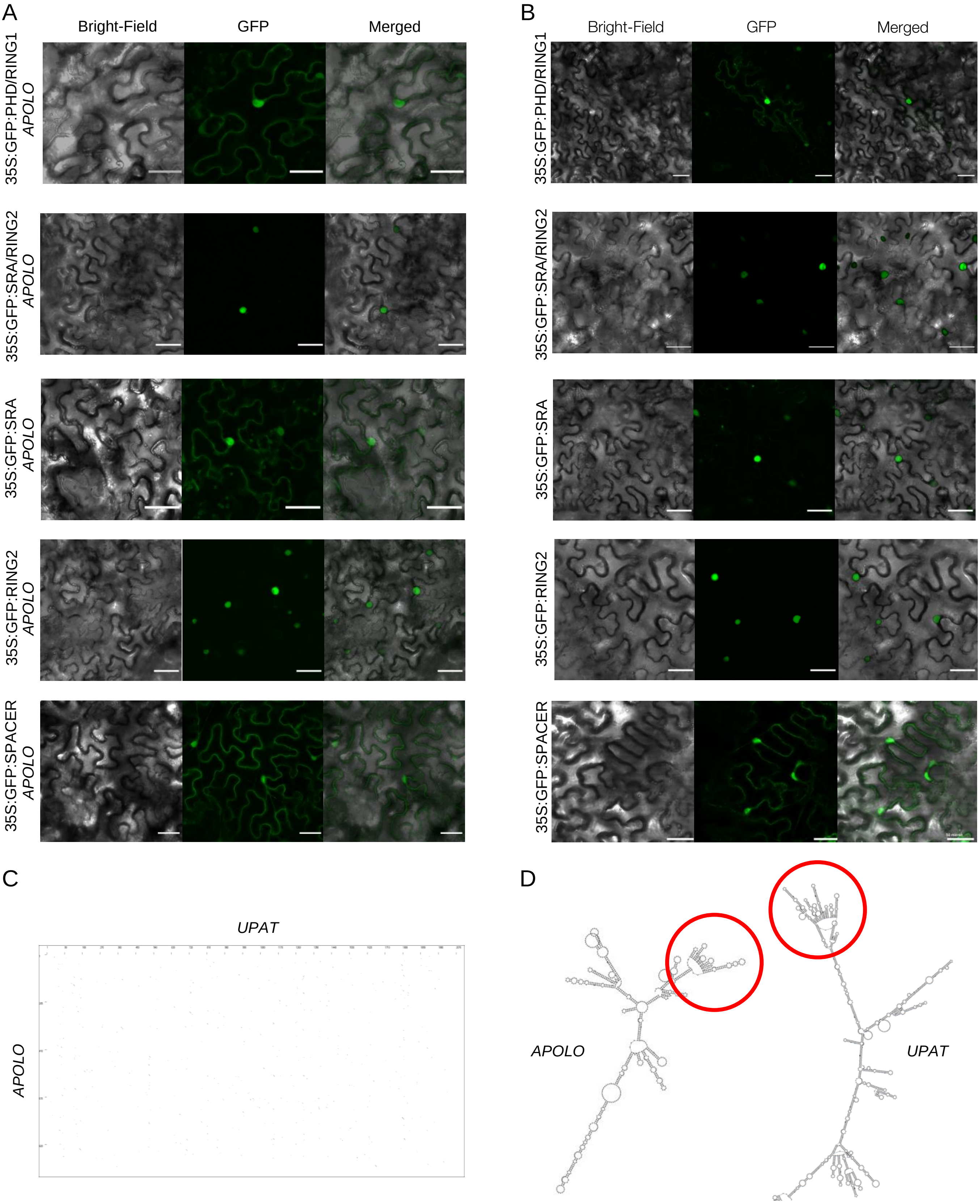

Figure S9: *APOLO* and *UPAT* lncRNAs share mecanisms of interaction with methylcytosine-binding proteins but no sequence similarity

(**A-B**) Sub-cellular localization of *GFP-PHD/RING1*, *GFP-SRA/RING2*, *GFP-SRA*, *GFP-RING2* and *GFP-SPACER* translational fusions transiently expressed from the 35S-CaMV promoter in *Nicotiana benthamiana* leaves in presence (in **A**) or absence (in **B**) of *APOLO*. Bright-field image (left), GFP fluorescence alone (middle) and bright-field/GFP fluorescence merged images (right) are shown. Scale bars, 50µm. One representative picture out of three biological replicates is shown. (**C**) Dot plot pairwise sequence comparison of *APOLO* and *UPAT* lncRNAs showing low similarity between the two sequences. BioEdit program is published in (133). (**D**) Prediction of the secondary structure of *APOLO* and *UPAT* by using RNAfold with default parameters (67, 68)). Red circles indicate potentially similar structurated regions. Determination of the secondary structure by biochemical approaches is required to propose new hypotheses of common structure-based functions of lncRNAs.

Additional File 1, Fig. S10

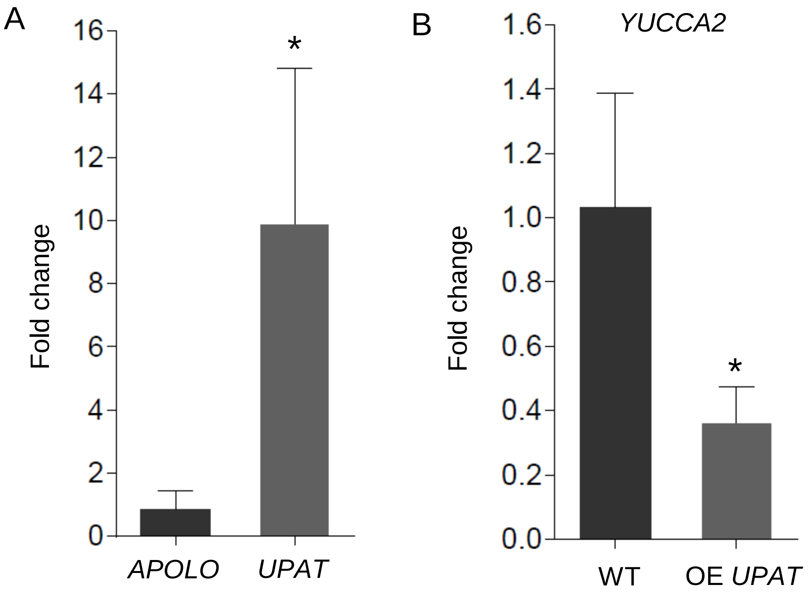

Figure S10: Constitutive expression of the lncRNA *UPAT* in Arabidopsis seedlings impairs *YUCCA2* transcriptional accumulation

(**A**) *UPAT* transcript levels in OE *UPAT* Arabidopsis seedlings determined as the ratio with endogenous *APOLO* accumulation taken as 1. (**B**) *YUCCA2* basal transcript levels in wild-type (WT) and OE *UPAT* 14-day-old seedlings. Bars represent average ± SD (n = 3 independent pools of seedlings). Asteriks (*) indicate Student's t-test ≤ 0.05 (n = 3). In (A), *UPAT* levels were compared with *APOLO* endogenous levels.

Additional File 1, Fig. S11

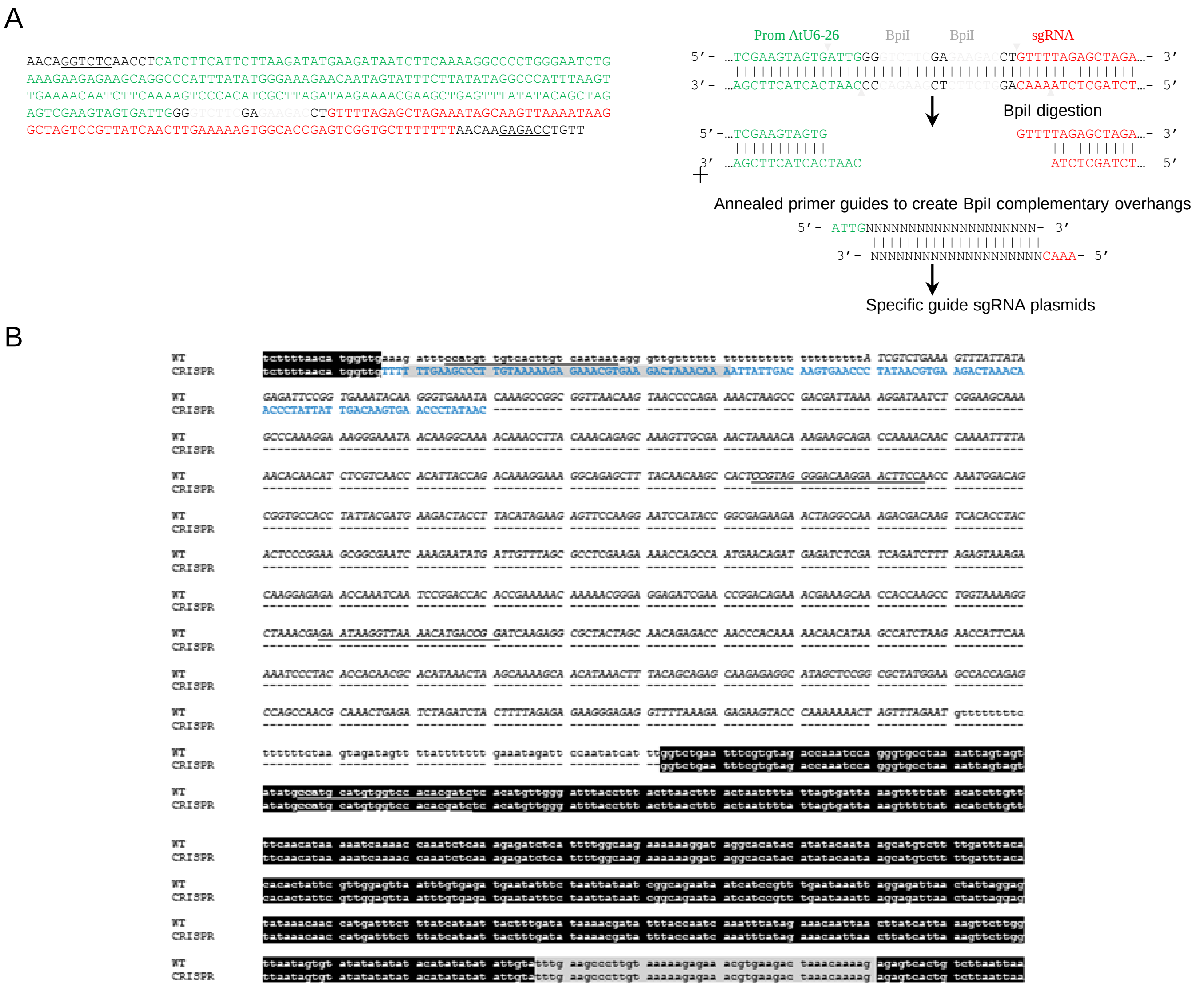

Figure S11: CRISPR/Cas9 strategy for *APOLO* deletion

(**A**) AtU6-26 promotor (green), BpiI restriction sites (blue) and sgRNA scaffold (red) between two BsaI restriction sites (underlined) synthetized sequence, subcloned in pGGA000 to create pGGA010. Below is the central sequence of pGGA/B/C/D010 plasmids. After BpiI (blue) digestion, annealed oligo guides are ligated through complementary overhangs. AtU6-26 promotor and sgRNA scaffold are respectively indicated in green and red. (**B**) AT2G34655 (*APOLO*) sequence is indicated in capital and italic letters, sgRNA guides are underlined with PAM in bold, inserted DNA sequence in the CRISPR/Cas9 *APOLO* line is indicated in blue, duplicate DNA region and similarities are respectively highlighted in grey and black.
