# Supplementary material for: The Arabidopsis APOLO and human UPAT sequence-unrelated long noncoding RNAs can modulate DNA and histone methylation machineries in plants
Source: Genome Biol. 2022 Aug 29;23:181. doi: 10.1186/s13059-022-02750-7 (PMC9422110; doi:10.1186/s13059-022-02750-7)
Supplement: Supplementary file 3 — Additional file 3. Peer review history. [file 13059_2022_2750_MOESM3_ESM.docx]

**Review history**

**First round of review**

**Reviewer 1**

Sequence-unrelated long noncoding RNAs converged to modulate the activity of conserved epigenetic machineries across kingdoms
GBIO-D-21-01236
In this manuscript, Fonouni-Fardi and colleagues proposes a model where two long non-coding RNAs with little sequence similarity and from two different organisms regulate gene expression via a conserved mechanism. This mechanism is activated by the binding of the lncRNA to proteins that control the epigenic environment of the regulated gene's promoter.
I think this a solid and well-written manuscript that has the potential to be published in Genome Biology. However, I have some concerns that should be addressed before it can be accepted for publication. Foremost, the title makes a big claim that this is conserved across kingdoms, this is not shown by the experiments in the manuscript. Thus, the authors might want to change the title to better reflect their results.
Major concerns:
1. qPCR:
qPCR normalizations have been performed using PP2A as a reference gene universally by the authors wherever applicable. Expression of PP2A genes is suggested to undergo changes during heat stress (Latorre et. al, 1997). Also, eFP browser indicate that chosen PP2A (AT1G13320) might respond to abiotic stress such as heat. Consequently, considering the significance of qPCR results, PP2A (AT1G13320) perhaps is not the best choice for the normalization of Ct values for qPCR data presented in this manuscript. Contemplating narrow range of fold changes in gene expression at 29⁰ C vs 23⁰ C, authors maybe required to show evidence that PP2A (AT1G13320) expression remains unaltered at 23⁰ C and 29⁰ C.
2. vim mutants:
- Downregulation levels of VIM1 in vim1 insertional mutants upon heat stress is missing, authors have also not shown at what developmental stage and temperature (supplementary figure 2C), the transcripts were quantified for VIM1 in vim 1-2, 1-3 mutants? This is critical because in ChIRP experiments authors have preferred to include vim 1-3 mutant that carry T-DNA insertion in the intron (carry a possibility of producing mature mRNA) unlike in vim 1-2 where T-DNA is in exon.
- The authors state that only VIM1 is involved in thermomorphogenesis. In Fig S3, VIM2 is downregulated in response to heat. This is not elaborated in the manuscript, but it could have an opposite effect compared to VIM1.
3. APOLO RNAi and full length CRISPR knockout lines:
- While APOLO OE lines phenotype is slightly stronger. However, mean hypocotyl ratio for APOLO RNAi and CRISPR-APOLO line largely falling in same range and its harder to notice tendency opposite to that of APOLO OE lines if applied statistical tests are given less weight. Therefore, more stringent statistical analysis might be performed again using absolute original values from WT, RNAi lines and CRISPR knockout lines at 29⁰ C directly instead of comparing mean ratios from 29⁰ and 23⁰ C.
- APOLO have been suggested to act upon multiple targets in trans. To clearly distinguish its actions from potentially unknown cis affects, CRISPR-APOLO line (together with RNAi lines) should have been employed in ChRIP assays. This could have served a nice negative control as well.
- Altered YUC2 expression levels are selectively shown only in APOLO-OE, vim 1-3, lhp1 mutant. However, transcript abundance for YUC2 in CRISPR-APOLO, RNAi lines, VIM-OE is not shown despite the inclusion of associated hypocotyl length phenotype. These results are necessary to not only to correlate phenotypic effects of CRISPR-APOLO, RNAi lines, VIM-OE with YUC2 expression regulation but also to substantiate the fact that YUC2 is a 'direct' target for APOLO. Additional experiments including overexpression of YUC2 in CRISPR-APOLO background for phenotypic effects and/ or data showing results for prom::YUC2 activity in CRISPR-APOLO background is perhaps necessary to be secure that YUC2 activity is regulated by APOLO.
4. UPAT:
- PRC1 and LHP1 could be largely promiscuous in their recognition and binding pattern for a range of coding and non-coding RNAs. The big claim by authors regarding similar consequences of varied mechanisms of actions for APOLO and UPAT raises concerns about specificity of UPAT effects over YUC2 promoter. UPAT was overexpressed in WT Col-0 background instead in CRISPR-APOLO line. How the overexpression of an unrelated UPAT overtakes and doesn't interfere with endogenous levels of APOLO to specifically act upon YUC2 promoter. How authors exclude the fact that it's not an 'indirect consequence' of higher binding affinity of UPAT, compared to APOLO, with promiscuous LHP1-VIM1 complex leading to transcriptional changes at loci regulated by LHP1-VIM1 complex such as YUC2 promoter. To validate these claims, authors might be required to show that global transcriptomic changes in APOLO-OE and OE-UPAT occurs in similar fashion. Also, to show whether UPAT is liberated from YUC2 promoter upon heat stress, YUC2 transcripts levels may be quantified at 23⁰ C and 29⁰ C in OE-UPAT line.
- Considering described UPAT effects over YUC2 promoter and no sequence homology with APOLO, it is necessary to explain whether formation of R-loop is integral or secondary for the specificity driven trans-actions of APOLO over YUC2 promoter. Does disruption of bases (introduction of noncomplementarity) in transcript region of APOLO that forms R-loop still carry the potential to drive transcriptional regulation of YUC2?
- I believe there is a need for some structure data to further strengthen the model proposed by the authors. Structure data from APOLO and UPAT at 23 and 29 ⁰ C would be a key experiment.
5. DRIP:
The DRIP analysis should be expanded. Does the R-loop disappear in 29 ⁰ C (this would be the prediction from the model)? Also, inclusion of the vim mutant or APOLO-OE would further strengthen the claims of the model.
6. BiFC:
The authors should also include some nuclear marker like DAPI staining.
7. vim x lhp1 double mutant:
It would be nice to have some genetic evidence of the model, such as a vim x lhp1 double mutant.
Minor concerns:
1. line 602-606- It is unclear how exactly heat stress experiments were performed. Whether plants were grown 23⁰ C till 4 days and then shifted to 3h at 23⁰ C followed by transfer to 29⁰ C or maintained at 23⁰ C? At what temperature seed germination and initial growth occurred? At which time point (days after germination) hypocotyl quantification was performed? In addition, it is also unclear how the ratio was obtained for hypocotyl length elongation? Which corresponding individual data points (independent hypocotyl length measurements) from 29⁰ C were divided by which data sets from 23⁰ C to obtain individual values for final hypocotyl length ratio as indicated by data points in violin plots and for which data sets statistical comparisons are performed (derived values for ratio vs original measurements for hypocotyl length elongation at 29⁰ C?
2. line 804- please include method of analysis for qPCR data, for e.g., primer efficiency (PE)-ΔΔCT.
3. line 817-818- What is indicated by n? does the sample mean represent independent data set from separate experiments or the number of groups? For instance, applied non-parametric statistical tests in supplementary figure 4 (E, F vs G) does not follow the described criteria for tests i.e. number of groups >2. Please specify the reasons as why in supplementary figure 4F Kruskal-Wallis test was applied while in supplementary figure 4G Mann-Whitney test is employed despite its limitation to compare for only two groups.
4. Figure 1E and supplementary figure 4G- hypocotyl length mean ratio is shown consistently and close to 2.5 for WT wherever applicable. However, WT mean ratio values upon comparison with associated VIM mutants lines (~2.5 mean, figure 1E) and overexpression lines (~2.0 mean, supplementary figure 4G) are different. Since the hypocotyl length phenotype follow a very narrow range, authors might be required to clarify why WT is different in these two cases as it potentially suggests bias towards selection of favourable WT in these data sets.
5. Figure2B (right panel), figure 3E (right panel), supplementary figure 4 (A, B, C, D), Supplementary figure 7 (B, G) -please check the representative pictures at 23⁰ C and 29⁰ C on par with mean values from corresponding quantitative box plots.
6. Supplementary figure 10B- please include the temperature and growth conditions (23⁰ C or 29⁰ C) at which transcript quantification for YUCCA2 was performed in OE-UPAT.
7. What is the phenotype of VIM1-OE?

**Reviewer 2**

Long non-coding RNAs (lncRNAs) play important roles in recruiting chromatin regulatory proteins and regulating three-dimensional chromatin organization. The Arabidopsis lncRNA APOLO was previously shown to interact with the Polycomb-group (PcG) member LHP1. Here, the authors identify a novel interaction with the DNA methylation regulator VIM1. They identify a potential role for the APOLO-VIM1-LHP1 complex in regulating thermophogenesis by transcriptional regulation of the auxin biosynthesis gene YUCCA2.
Generally, the potential novelty of the findings is high, as it connects two major epigenetic pathways in the regulation of thermomorphogenesis, which may be bridged by the lncRNA APOLO.
However, I have major concerns on the conclusions, the presentation and evaluation of the data and statistical analyses (see below), which make a proper evaluation of this manuscript difficult.
Note: I did not find sup table 2 and 3, so I could not evaluate these.

Major comments:
1. The screen which identified VIM1 is not described at all. Here, a table of interactors and negative control results need to be provided. It also needs to be explained why VIM1 (and not others) were analysed and chosen.
2. Statistical analyses are missing in most of the figures, for ChIP-PCR and RT-PCR, e.g. Fig. 1B, C, 2C etc.
3. The work is focused on VIM1, but information is lacking on VIM2 or VIM3 which would be perfect controls to show that the interaction of VIM1-APOLO is specific (referring to figure 1B). While VIM1 is slightly induced (significance unclear) at higher temperature, VIM2 is repressed, but this is not further analysed, e.g. to see whether vim2 mutants have elongated hypocotyl.
4. While the two vim mutants have a shorter hypocotyl at 29 °C, a complementation of the phenotypes/mutants need to be provided.
5. Transcriptomic data: I miss here the comparisons at 29 °C for OE APOLO-1 and vim1-3 and wt 23 °C.
6. While YUC2 appears to be regulated by APOLO and VIM1, its relevance for the phenotype is unclear. Currently, it is picked as one candidate, but genetic analyses would need to be provided.
7. The authors show decreased LHP1 binding in a vim1-3 mutant (fig.3). I am missing controls, also for other ChIP analyses, that the overall level of LHP1 (at other target genes), is not affected. As the level of YUC2 expression is changed in the mutants, this could be also an indirect effect. Again, genetic analyses would be required to support these correlations.

Minor comments:
- L112: PRC1 does not maintain H3K27me3, this needs to be properly described
- The hypocotyl elongation assays are only presented as ratios 29/23 °C, but should also be presented just at 23 °C and 29 °C, with statistical analyses compared to the wildtype. The table provided is not sufficient, as it lacks the statistical analyses.

**Authors’ response to reviewers**

Reviewer #1: Review

Sequence-unrelated long noncoding RNAs converged to modulate the activity of

conserved epigenetic machineries across kingdoms GBIO-D-21-01236

In this manuscript, Fonouni-Fardi and colleagues proposes a model where two long non-coding RNAs with little sequence similarity and from two different organisms regulate gene expression via a conserved mechanism. This mechanism is activated by the binding of the lncRNA to proteins that control the epigenic environment of the regulated gene's promoter.

I think this a solid and well-written manuscript that has the potential to be published in Genome Biology. However, I have some concerns that should be addressed before it can be accepted for publication.

Foremost, the title makes a big claim that this is conserved across kingdoms, this is not shown by the experiments in the manuscript. Thus, the authors might want to change the title to better reflect their results.

R. We thank the Reviewer for his/her interest in our work and his/her valuable feedback.

We have now included in Figure 5C a panel showing how the human DNMT1/UHRF1/HP1+lncRNA complex has a parallel in Arabidopsis, including the homolog proteins MET1/VIM1/LHP1+lncRNA. Although the claim is big, we think that the fact of UPAT mimicking APOLO action in plant cells (including the new data included in this improved version of the manuscript, notably the RNAseq results), is an important breakthrough towards the cross talk between different kingdoms of lncRNA action. We wanted to catch the attention of the community and the wide audience of Genome Biology to this conserved action in the frame of comparing the literature in animals and plants to draw relevant conclusions and propose new hypotheses.

Nevertheless, we are open to other title if the Editor or the reviewer can suggest a better version.

Major concerns:

1.	qPCR:

qPCR normalizations have been performed using PP2A as a reference gene universally by the authors wherever applicable. Expression of PP2A genes is suggested to undergo changes during heat stress (Latorre et. al, 1997). Also, eFP browser indicate that chosen PP2A (AT1G13320) might respond to abiotic stress such as heat. Consequently, considering the significance of qPCR results, PP2A (AT1G13320) perhaps is not the best choice for the normalization of Ct values for qPCR data presented in this manuscript. Contemplating narrow range of fold changes in gene expression at 29⁰ C vs 23⁰ C, authors maybe required to show evidence that PP2A (AT1G13320) expression remains unaltered at 23⁰ C and 29⁰ C.

R. This is a very important point, indeed. For equivalent amounts of cDNA, PP2A exhibited constant Cts across the samples assessed. Moreover, we have now checked the behavior of PP2A in all the RNA-Seq approaches included in this work (WT 23°C vs. 29°C, OE APOLO, vim1-3 and OE UPAT plants), and it appeared as a housekeeping gene. Considering that PP2A is responsive to stress in other developmental contexts and to clarify that in our conditions (mild stress different from Latorre et al., 1997) PP2A behaves constitutively, we have added a phrase in the Methods section making this point clear (lines 880-881).

2.	vim mutants:

-	Downregulation levels of VIM1 in vim1 insertional mutants upon heat stress is missing, authors have also not shown at what developmental stage and temperature (supplementary figure 2C), the transcripts were quantified for VIM1 in vim 1-2, 1-3 mutants? This is critical because in ChIRP experiments authors have preferred to include vim 1-3 mutant that carry T-DNA insertion in the intron (carry a possibility of producing mature mRNA) unlike in vim 1-2 where T-DNA is in exon.

R. We have now included the analysis of transcriptional levels of VIM1 at 23°C vs. 29°C in 4-day-old seedlings in Supplementary Figure 2C. Just to clarify, ChIRP-qPCR was performed in WT, RNAi-APOLO (and CRISPR-APOLO in this new version) lines, not in vim1 mutants. Based on the similar phenotype exhibited by vim1-2 and vim1-3 (Figure 1E), we chose the latest for transcriptomics and further analyses.

-	The authors state that only VIM1 is involved in thermomorphogenesis. In Fig S3, VIM2 is downregulated in response to heat. This is not elaborated in the manuscript, but it could have an opposite effect compared to VIM1.

R. Thanks for pointing this out. Based on both Reviewer’s comments, we have now cloned 35S:GFP:VIM2 and performed a RIP-qPCR in N. benthamiana leaves. We now show that although very weakly compared to VIM1, VIM2 is able to also recognize APOLO in planta (Supplementary Figure 3). We have expanded our Results and Discussion sections (lines 170-175 and 504-509), proposing that the VIM2-APOLO interaction may occur during Arabidopsis development, and that lncRNA-mediated regulation of VIM proteins seems to be a general feature.

3.	APOLO RNAi and full length CRISPR knockout lines:

-	While APOLO OE lines phenotype is slightly stronger. However, mean hypocotyl ratio for APOLO RNAi and CRISPR-APOLO line largely falling in same range and its harder to notice tendency opposite to that of APOLO OE lines if applied statistical tests are given less weight. Therefore, more stringent statistical analysis might be performed again using absolute original values from WT, RNAi lines and CRISPR knockout lines at 29⁰ C directly instead of comparing mean ratios from 29⁰ and 23⁰ C.

R. In Supplementary Table 1 we have now included all the respective tests at 23°C and 29°C, separately.

-	APOLO have been suggested to act upon multiple targets in trans. To clearly distinguish its actions from potentially unknown cis affects, CRISPR-APOLO line (together with RNAi lines) should have been employed in ChRIP assays. This could have served a nice negative control as well.

R. Thanks for this suggestion. We have now included a new ChIRP experiment using CRISPR-APOLO seedlings compared to WT and RNAi APOLO (Figure 2E) that confirm our results.

-	Altered YUC2 expression levels are selectively shown only in APOLO-OE, vim 1- 3, lhp1 mutant. However, transcript abundance for YUC2 in CRISPR-APOLO, RNAi lines, VIM-OE is not shown despite the inclusion of associated hypocotyl length phenotype. These results are necessary to not only to correlate phenotypic effects of CRISPR-APOLO, RNAi lines, VIM-OE with YUC2 expression regulation but also to substantiate the fact that YUC2 is a 'direct' target for APOLO. Additional experiments including overexpression of YUC2 in CRISPR-APOLO background for phenotypic effects and/ or data showing results for prom::YUC2 activity in CRISPR-APOLO background is perhaps necessary to be secure that YUC2 activity is regulated by APOLO.

R. We have now included the analyses of YUC2 transcript levels in all the related backgrounds (Supplementary Figure 6A, together with additional VIM1-APOLO targets), as indicated in lines 240-243 and 251-256. proYUC2:GUS lines were used to further support that YUC2 is a warmth-responsive gene in the developmental context studied here. Crossing proYUC2:GUS with APOLO deregulated lines may be interesting. However, APOLO recognizes YUC2 first exon, not the promoter (as shown in Figure 2D and explained in lines 260-265). Therefore, the epigenetic regulation of YUC2 mediated by APOLO cannot be assessed by such an approach. Furthermore, the epigenetic status may not be equivalent on the transgene than on the WT promoter which may lead to ambiguous results. Finally, expressing YUC2 in the CRISPR- APOLO background would help to understand better the genetics behind this mechanism, although it will not contribute to the understanding of the lncRNA-mediated epigenetic regulation of the locus, which is the main focus of this work. Moreover, additional APOLO targets are likely involved in this auxin-dependent response, and deeper genetics on YUC2 would not be sufficient to contradict our biochemical approaches demonstrating APOLO direct recognition of the YUC2 locus and the related epigenetic regulation.

4.	UPAT:

-	PRC1 and LHP1 could be largely promiscuous in their recognition and binding pattern for a range of coding and non-coding RNAs. The big claim by authors regarding similar consequences of varied mechanisms of actions for APOLO and UPAT raises concerns about specificity of UPAT effects over YUC2 promoter. UPAT was overexpressed in WT Col-0 background instead in CRISPR-APOLO line. How the overexpression of an unrelated UPAT overtakes and doesn't interfere with endogenous levels of APOLO to specifically act upon YUC2 promoter. How authors exclude the fact that it's not an 'indirect consequence' of higher binding affinity of UPAT, compared to APOLO, with promiscuous LHP1-VIM1 complex leading to transcriptional changes at loci regulated by LHP1-VIM1 complex such as YUC2 promoter.

R. We decided to study OE UPAT compared to OE APOLO in the same WT background, so as to compare the molecular output of both lines. Please note that we did not propose that UPAT recognizes specifically YUC2, a DNA-RNA interaction which likely relies on sequence complementarity. Instead, we proposed that the overaccumulation of UPAT in the nucleus mimics the overaccumulation of APOLO, and both can decoy VIM1 and LHP1 away from chromatin targets. In the new model in Figure 5B we included UPAT and APOLO to make this point clear. We think that cross- complementation experiments are nice leads for future experiments but, precisely due to their large sequence variation, many constructs about biologically significant sequences inside these transcirpts should be characterized. This is clearly out of the scope of our ms.

To validate these claims, authors might be required to show that global transcriptomic changes in APOLO-OE and OE-UPAT occurs in similar fashion. Also, to show whether UPAT is liberated from YUC2 promoter upon heat stress, YUC2 transcripts levels may be quantified at 23⁰ C and 29⁰ C in OE-UPAT line.

R. As explained above, we do not consider that UPAT binds to the YUC2 locus. Alternatively, we have proposed that the over-accumulation of UPAT resembles APOLO overexpression. We have followed the Reviewer’s suggestion and we performed a new RNA-Seq approach comparing WT, OE APOLO and OE UPAT. As shown in Figure 4I, almost a half of DEG in OE APOLO were also deregulated (up or down, respectively) in OE UPAT, with pvalues of 10-127 and 10-264 (Supplementary Table 5). The result is amazing and we want to thank the Reviewer for his/her suggestion. With this new experiment, we can now demonstrate that the global effect of overexpressing UPAT on the Arabidopsis transcriptome significantly overlaps with the effect of APOLO overexpression.

-	Considering described UPAT effects over YUC2 promoter and no sequence homology with APOLO, it is necessary to explain whether formation of R-loop is integral or secondary for the specificity driven trans-actions of APOLO over YUC2 promoter. Does disruption of bases (introduction of noncomplementarity) in transcript region of APOLO that forms R-loop still carry the potential to drive transcriptional regulation of YUC2?

R. Consistently with our previous responses, we have now made it very clear also in the text (lines 550-553, legend) and in the model of Figure 5B that we do not think that UPAT forms R-loops over the YUC2 locus.

-	I believe there is a need for some structure data to further strengthen the model proposed by the authors. Structure data from APOLO and UPAT at 23 and 29 ⁰ C would be a key experiment.

R. We have now included a prediction of APOLO and UPAT secondary structure by using RNAfold, in Supplementary Figure 9 (and lines 419-420 in the text), and we stated (as perspectives for future research) that determination of the secondary structure by biochemical approaches is required to propose new hypotheses of common structure-based functions of lncRNAs (see the legend of the Supplementary Figure 9D). A phrase about the relevance of this point was already included in the Discussion (lines 536-538).

5.	DRIP:

The DRIP analysis should be expanded. Does the R-loop disappear in 29 ⁰ C (this would be the prediction from the model)? Also, inclusion of the vim mutant or APOLO- OE would further strengthen the claims of the model.

R. Thanks for such a valuable suggestion. We have now performed a DRIP-qPCR experiment at 23⁰C vs 29⁰C in WT and OE APOLO seedlings and we showed that the significant reduction in R-loop formation observed in WT plants is impaired in OE APOLO plants (Figure 2G).

6.	BiFC:

The authors should also include some nuclear marker like DAPI staining.

R. We have performed the BiFC again including the expression of mCherryto visualize nuclei and membranes (Supplementary Figure 8A).

7.	vim x lhp1 double mutant:

It would be nice to have some genetic evidence of the model, such as a vim x lhp1 double mutant.

R. Although this idea is good, please note that vim1 and lhp1 mutants show the same phenotypic trend (i.e. impaired hypocotyl elongation in warmth) and a similar repressive effect over YUC2, thus precluding a genetic analysis based on crosses. Moreover, lhp1 single mutant is already very pleiotropic.

Minor concerns:

1.	line 602-606- It is unclear how exactly heat stress experiments were performed. Whether plants were grown 23⁰ C till 4 days and then shifted to 3h at 23⁰ C followed by transfer to 29⁰ C or maintained at 23⁰ C? At what temperature seed germination and initial growth occurred? At which time point (days after germination) hypocotyl quantification was performed? In addition, it is also unclear how the ratio was obtained for hypocotyl length elongation? Which corresponding individual data points (independent hypocotyl length measurements) from 29⁰ C were divided by which data sets from 23⁰ C to obtain individual values for final hypocotyl length ratio as indicated by data points in violin plots and for which data sets statistical comparisons are performed (derived values for ratio vs original measurements for hypocotyl length elongation at 29⁰ C?

R. As indicated lines 664-666, “For phenotypic characterization of hypocotyl elongation, seedlings were grown for 3h at 23°C and transferred to 29°C or maintained at 23°C for 4 days.”. Seed germination and initial growth occur at 23°C. Hypocotyl quantification is performed 4 days after germination.

Individual data points from 29°C are divided by the mean of data points from 23°C to obtain individual values for final hypocothyl length ratio. Comparisons are performed between genotypes using these derived values for ratio. We have now additionally included all the stats between mutants and their respective WT controls in Supplementary Table 1 at 23°C and 29°C, independently.

2.	line 804- please include method of analysis for qPCR data, for e.g., primer efficiency (PE)-ΔΔCT.

R. This is now clarified in Methods (lines 879-881).

3.	line 817-818- What is indicated by n? does the sample mean represent independent data set from separate experiments or the number of groups? For instance, applied non-parametric statistical tests in supplementary figure 4 (E, F vs G) does not follow the described criteria for tests i.e. number of groups >2. Please specify the reasons as why in supplementary figure 4F Kruskal-Wallis test was applied while in supplementary figure 4G Mann-Whitney test is employed despite its limitation to compare for only two groups.

R. In line 817-818 n indicates the number of independent groups compared. We have now modified the statement to make it clear. In figure 4C, G, I a Kruskal-Wallis test was applied while in H a Mann-Whitney test was applied. We apologize for the mistake in writing the corresponding legend.

4.	Figure 1E and supplementary figure 4G- hypocotyl length mean ratio is shown consistently and close to 2.5 for WT wherever applicable. However, WT mean ratio values upon comparison with associated VIM mutants lines (~2.5 mean, figure 1E) and overexpression lines (~2.0 mean, supplementary figure 4G) are different. Since the hypocotyl length phenotype follow a very narrow range, authors might be required to clarify why WT is different in these two cases as it potentially suggests bias towards selection of favourable WT in these data sets.

R. WT are grown and collected together with mutants at each new generation to avoid potential generational effects. Although growth conditions are carefully controlled, it is common to observe slight variations in the response of WT generated independently.

5. Figure2B (right panel), figure 3E (right panel), supplementary figure 4 (A, B, C, D), Supplementary figure 7 (B, G) -please check the representative pictures at 23⁰ C and 29⁰ C on par with mean values from corresponding quantitative box plots.

R. Pictures selected are representative of the corresponding measurements, including the new data of vim1 complemented lines (in Supplementary Figure 4).

6.	Supplementary figure 10B- please include the temperature and growth conditions (23⁰ C or 29⁰ C) at which transcript quantification for YUCCA2 was performed in OE- UPAT.

R. This is now clarified in the corresponding legend. Transiently transformed leaves were obtained in 14-day-old seedlings. However, we have now used stably transformed plants for the new RNA-Seq approach including OE UPAT vs WT.

7.	What is the phenotype of VIM1-OE?

R. This was already shown in Supplementary Figure 4F and I.

Reviewer #2: Long non-coding RNAs (lncRNAs) play important roles in recruiting chromatin regulatory proteins and regulating three-dimensional chromatin organization. The Arabidopsis lncRNA APOLO was previously shown to interact with the Polycomb- group (PcG) member LHP1. Here, the authors identify a novel interaction with the DNA methylation regulator VIM1. They identify a potential role for the APOLO-VIM1-LHP1 complex in regulating thermophogenesis by transcriptional regulation of the auxin biosynthesis gene YUCCA2.

Generally, the potential novelty of the findings is high, as it connects two major epigenetic pathways in the regulation of thermomorphogenesis, which may be bridged by the lncRNA APOLO.

However, I have major concerns on the conclusions, the presentation and evaluation of the data and statistical analyses (see below), which make a proper evaluation of this manuscript difficult.

Note: I did not find sup table 2 and 3, so I could not evaluate these.

R. We thank the Reviewer for his/her positive appreciation of our work. We are sorry that in the first submission we uploaded by mistake Supplementary Table 2 twice, instead of Supplementary Table 3. They are both available now.

Major comments:

1. The screen which identified VIM1 is not described at all. Here, a table of interactors and negative control results need to be provided. It also needs to be explained why VIM1 (and not others) were analysed and chosen.

R. The exploratory screen was explained in the main text (lines 140-146) and in the Methods section (lines 735-793, in-detail description). It was stated that probes against LacZ were used as a negative control. This was considered an exploratory approach because we had 1 replicate of ODD and 1 of EVEN probes against APOLO. We cannot offer a full list of potential interactors because we do not have enough replicates to perform quantitative proteomics. As we did for WRKY42 (Moison et al., 2021, Mol Plant), we confirmed here the interaction between APOLO and VIM1 by RIP in Arabidopsis and N. benthamiana (Figure 1B). In addition, we included here a ChIRP- dot blot in Arabidopsis plants expressing GFP:VIM1 (and using antibodies against GFP; Figure 1A and Supplementary Figure 1A). Altogether, upon the identification of VIM1 as a potential APOLO partner, we further validated this interaction by three additional independent approaches, including a ChIRP-dot blot itself.

2. Statistical analyses are missing in most of the figures, for ChIP-PCR and RT-PCR,

e.g. Fig. 1B, C, 2C etc.

R. We have included all the statistical analyses where required.

3. The work is focused on VIM1, but information is lacking on VIM2 or VIM3 which would be perfect controls to show that the interaction of VIM1-APOLO is specific (referring to figure 1B). While VIM1 is slightly induced (significance unclear) at higher temperature, VIM2 is repressed, but this is not further analysed, e.g. to see whether vim2 mutants have elongated hypocotyl.

R. Thanks for pointing this out. Based on both Reviewer’s comments, we have now cloned 35S:GFP:VIM2 and performed a RIP-qPCR in N. benthamiana leaves. We now show that although very weakly compared to VIM1, VIM2 is able to recognize APOLO in planta (Supplementary Figure 3). We have expanded our Results and Discussion sections (lines 170-175 and 504-509), proposing that the VIM2-APOLO interaction may occur during Arabidopsis development, and that lncRNA-mediated regulation of VIM proteins seems to be a general feature.

4. While the two vim mutants have a shorter hypocotyl at 29 °C, a complementation of the phenotypes/mutants need to be provided.

R. We have now added the phenotyping of vim1-3 plants transformed with proVIM1:GFP:VIM1 in Supplementary Figure 4C and D.

5. Transcriptomic data: I miss here the comparisons at 29 °C for OE APOLO-1 and vim1-3 and wt 23 °C.

R. Sorry, we uploaded a mistaken Sup Table 2. We have compared the transcriptomics of WT plants a 23°C and 29°C, together with OE APOLO-1 and vim1-3 at 23°C (Supplementary Table 2, 3 and Figure 2).

6. While YUC2 appears to be regulated by APOLO and VIM1, its relevance for the phenotype is unclear. Currently, it is picked as one candidate, but genetic analyses would need to be provided.

R. It was previously shown that APOLO also regulates auxin transport (Ariel et al., 2020 Mol Cell), which may be related to the thermomorphogenic response. The observed phenotype in response to warmth may be due to alternative pathways controlled by APOLO, and we have picked YUC2 as a novel and interesting target for validation of action of the LHP1-VIM1-APOLO complex. We have now clarified that YUC2 is just one among several common targets (lines 240-243 and 251-256 in Results and 512-513 in Discussion) and we have analyzed the transcriptional behavior of a subset of them including YUC2 (Supplementary Figure 6A). Please note that yuc2 simple mutant is already impaired in hypocotyl elongation and that proYUC2:GUS plants served to reveal YUC2 promoter activation at warm temperatures.

7. The authors show decreased LHP1 binding in a vim1-3 mutant (fig.3). I am missing controls, also for other ChIP analyses, that the overall level of LHP1 (at other target genes), is not affected. As the level of YUC2 expression is changed in the mutants, this could be also an indirect effect. Again, genetic analyses would be required to support these correlations.

R. This is an excellent suggestion. We have now included LHP1 binding analysis of an APOLO-independent gene as a negative control, i.e. AT4G23720 (taken from our previous work by Veluchamy et al., 2016) in Figure 3H and I. Furthermore, we have also expanded our discussion including the possibility that indirect effects may also be contributing to the interplay between H3K27me3 and meDNA (lines 629-632).

Minor comments:

- L112: PRC1 does not maintain H3K27me3, this needs to be properly described

R. Sorry for the misleading phrase. We meant “maintained” by LHP1, which in plants is considered a component of PRC1 (and also a bridge between PRC1 and 2, including the activity of H3K27me3 spreading, as we showed in Veluchamy et al., 2016). We have now rephrased the sentence to avoid the misunderstanding, thank you.

- The hypocotyl elongation assays are only presented as ratios 29/23 °C, but should also be presented just at 23 °C and 29 °C, with statistical analyses compared to the wildtype. The table provided is not sufficient, as it lacks the statistical analyses.

R. Thanks for the suggestion, we have now included all the stats between mutants and their respective WT controls in Supplementary Table 1 at 23°C and 29°C, independently.

**Second round of review**

**Reviewer 1**

Sequence-unrelated long noncoding RNAs converged to modulate the activity of conserved epigenetic machineries across kingdoms
GBIO-D-21-01236R1
I would like to start by saying that the authors have made a great effort to improve the manuscript in response to the reviewer’s comments from the original submission. However, there are still a few concerns that need to be addressed before it can be published in Genome Biology, in my opinion.
I still think the title is too exaggerated and probably needs to be changed. A full fledged molecular and in-depth genetic characterization for UPAT may be needed (in Arabidopsis) together with biochemical data presented in the study. Authors are pushing too much the fact lncRNAs from humans and Arabidopsis share commonalities in their mechanisms. However, this commonality is due to the fact that chromatin remodelers such as VIM and LHP are promiscuous in their RNA binding capacity and can be recruited to target loci by sequences unrelated but structurally similar lncRNAs, and this is a known fact, explaining the global transcriptional effects of UPAT in Arabidopsis. Nevertheless, these results (UPAT, APOLO transcriptomics) are novel, interesting and advances the field of lncRNAs. My suggestion would be to include APOLO and UPAT in the title and remove the “across kingdoms” since this is not shown in the manuscript.

A concern is that two independent APOLO OE lines shows significantly decreased hypocotyl length (p value- <0.005) at 23 degrees already. In contrast, YUC2 knock out (yuc2) under normal conditions shows no changes in hypocotyl length meaning that YUC2 has no role in hypocotyl elongation at 23 degrees, as shown in suppl table. Therefore, Considering the proposed model that at 23 degrees APOLO-VIM-LHP1 complex keeps YUC2 downregulated, why in both OE-APOLO lines including in one APOLO RNAi line hypocotyl length is affected at 23 degrees when hypocotyl length in yuc2 at 23 remains unaffected? This in planta effect at 23 degree contradicts with presented biochemical evidence in the manuscript. Could authors explain? Based upon these observations, the claims by authors regarding regulation of YUC2 by APOLO cannot be considered true.

Another concern is regarding PromYUC2::GUS line data. Under normal conditions (23 degrees) PromYUC2::GUS shows no reporter activity. In this line only promoter sequence is included excluding exons. As shown and replied by authors (fig. 2D) that APOLO recognizes first exon not the promoter and that exon1 is integral to YUC2 regulation by APOLO together with VIM1-LHP1. At 23 degrees PromYUC2::GUS must shows reporter activity because APOLO should be unable to specifically recognize it as a target as exon is missing. On the contrary and argument by authors in reviewers’ response, more importantly promoter alone is responding normally upon heat stress like WT indicating heat response akin to endogenous YUC2 loci (fig1c) which imply YUC2 regulation independent of APOLO. As per proposed model and data presented, this data (PromYUC2::GUS line) completely contradicts the proposed mechanism of YUC2 regulation by APOLO. Therefore, the claims made by authors regarding YUC2 regulation by APOLO doesn’t appear to be completely true.

Figure 1C- The description is missing in the manuscript.

Suppl. Fig 10 please include statistics. Also, wherever mentioned in the manuscript, the term ‘t-test’ should be replaced with Student’s t-test (maybe author can read story behind use of pseudonym ‘Student’), unless otherwise stated which type of test was applied in analysis.

In suppl. Fig 6A, the legend says VIM quantification of transcripts. Are authors showing VIM1 levels or YUCCA2 levels? Which one is correct? Based upon reviewers’ responses, it is assumed that transcript quantification is for YUCCA2. Please correct it. In addition, what is OE-VIM1-1? is this the overexpression of VIM1 in vim1-1 insertional mutant? Or this is X-axis labelling error?

The RT-qPCR primers for YUCCA2 are missing in provided table. Did authors use ChIP-qPCR primers for RT-qPCR as well?

L42-43, L135- please include a phrase, evolutionary unrelated lncRNA with potentially similar structures may exert similar functions

Please include information/figure about the nature of genes that are commonly affected in OE-APOLO and OE-UPAT.

**Reviewer 2**

The authors have addressed most of my concerns and the manuscript has improved a lot.
Still a few points remain:

Previous remark 3: I still miss the genetic analysis of vim2 and vim3 mutants to analyse their role in thermomorphogenesis.

Previous remark 6: Genetic interaction between YUC2 and VIM1 and APOLO: the authors stated that a genetic interaction analyses is not possible because yuc2 has shorter hypocotyls. I do not agree here as effects in a double mutant may be additive or epistatic and 35S::YUC2 may rescue the vim1 phenotype.

Generally thermomorphogenesis phenotypes: only the ratios of hypocotyl length at 23/29 °C are provided, also the data without the normalization should be provided for the experiments.

**Authors’ response to reviewers**

Reviewer #1: Review

Sequence-unrelated long noncoding RNAs converged to modulate the activity of conserved epigenetic machineries across kingdoms

GBIO-D-21-01236R1

I would like to start by saying that the authors have made a great effort to improve the manuscript in response to the reviewer’s comments from the original submission.

However, there are still a few concerns that need to be addressed before it can be published in Genome Biology, in my opinion.

R/ We thank the Reviewer for his/her positive comments on our manuscript.

I still think the title is too exaggerated and probably needs to be changed. A full fledged molecular and in-depth genetic characterization for UPAT may be needed (in Arabidopsis) together with biochemical data presented in the study. Authors are pushing too much the fact lncRNAs from humans and Arabidopsis share commonalities in their mechanisms. However, this commonality is due to the fact that chromatin remodelers such as VIM and LHP are promiscuous in their RNA binding capacity and can be recruited to target loci by sequences unrelated but structurally similar lncRNAs, and this is a known fact, explaining the global transcriptional effects of UPAT in Arabidopsis. Nevertheless, these results (UPAT, APOLO transcriptomics) are novel, interesting and advances the field of lncRNAs. My suggestion would be to include APOLO and UPAT in the title and remove the “across kingdoms” since this is not shown in the manuscript.

R/ We have modified the title to better reflect the content of the paper: The Arabidopsis APOLO and human UPAT sequence-unrelated long noncoding RNAs can modulate DNA and histone methylation machineries in plants.

We have also modified the conclusions of the abstract removing the statement about the activity across kingdoms. We hope that the Reviewer will find this title more suitable.

A concern is that two independent APOLO OE lines shows significantly decreased hypocotyl length (p value- <0.005) at 23 degrees already. In contrast, YUC2 knock out (yuc2) under normal conditions shows no changes in hypocotyl length meaning that YUC2 has no role in hypocotyl elongation at 23 degrees, as shown in suppl table. Therefore, Considering the proposed model that at 23 degrees APOLO-VIM-LHP1 complex keeps YUC2 downregulated, why in both OE-APOLO lines including in one APOLO RNAi line hypocotyl length is affected at 23 degrees when hypocotyl length in yuc2 at 23 remains unaffected? This in planta effect at 23 degree contradicts with presented biochemical evidence in the manuscript. Could authors explain? Based upon these observations, the claims by authors regarding regulation of YUC2 by APOLO cannot be considered true.

R/ Please note that direct regulation of YUC2 by APOLO is supported by solid evidence: (i) APOLO ChIRP-Seq, validated by ChIRP-qPCR in WT, RNAi and CRISPR-APOLO lines, (ii) R-loop formation impaired in APOLO knockdown/out lines,

(iii) YUC2 deregulation upon APOLO over expression. As we have already explained in the manuscript and the previous response to reviewers, we found that APOLO regulates YUC2 among a large subset of genes related to auxin synthesis, transport and response. Our data demonstrates that APOLO directly regulates YUC2 although YUC2 is not the only target mediating APOLO action. Thus, these other targets may explain the fact that we do not observe identical phenotypes in APOLO-deregulated lines and yuc2 mutants. This is explained and discussed in lines 253-257 and 516-520.

Another concern is regarding PromYUC2::GUS line data. Under normal conditions (23 degrees) PromYUC2::GUS shows no reporter activity. In this line only promoter sequence is included excluding exons. As shown and replied by authors (fig. 2D) that APOLO recognizes first exon not the promoter and that exon1 is integral to YUC2 regulation by APOLO together with VIM1-LHP1. At 23 degrees PromYUC2::GUS must shows reporter activity because APOLO should be unable to specifically recognize it as a target as exon is missing. On the contrary and argument by authors in reviewers’ response, more importantly promoter alone is responding normally upon heat stress like WT indicating heat response akin to endogenous YUC2 loci (fig1c) which imply YUC2 regulation independent of APOLO. As per proposed model and data presented, this data (PromYUC2::GUS line) completely contradicts the proposed mechanism of YUC2 regulation by APOLO. Therefore, the claims made by authors regarding YUC2 regulation

by APOLO doesn’t appear to be completely true.

R/ As mentioned before, the direct regulation of YUC2 by APOLO was demonstrated by a combination of approaches. Also, as mentioned above, YUC2 is one among many common targets of APOLO and VIM1. We needed to choose one target for further dissecting the APOLO-mediated mechanism of action over VIM1-LHP1. To this end, we searched for a gene expressed in the hypocotyl and modulated by warmth. YUC2 had been linked to the response to heat in flowers. We needed to show that it was relevant in the developmental context of our study. Thus, we showed that (i) yuc2 is affected in hypocotyl elongation, (ii) its induction by warmth is partially impaired in APOLO, VIM1 and LHP1-deregulaed plants, and (iii) that the YUC2 promoter is induced by warmth. These promoter-reporter plants shed light on the activity of the YUC2 promoter activation under the control, for example, of transcription factors. Our experiments served then to link YUC2 to warmth and to locate its expression in the hypocotyl. However, as mentioned in the manuscript and in our previous response to reviewers, this construct is in a different genomic position and does not reflect the complete activity of APOLO over YUC2. Even if we included the 1st exon, the use of transgenes (i.e. the T-DNA of ProYUC2:GUS) is well known to show epigenetic profiles different from the endogenous locus. Indeed, the genomic context of the insertion site is completely different and APOLO modulates the activity of epigenetic factors involved in YUC2 regulation in a specific genomic position. Therefore, reporter genes under the control of a given promoter serve to uncover the regulation by TFs controlling the activity of the promoter itself, but not the epigenetic fine-tuning or the chromatin status of the endogenous locus. Summing-up, we used the existing lines of ProYUC2:GUS to link YUC2 to the thermomorphogenic response in 4-day-old seedlings. Then we analyzed the endogenous YUC2 locus to define, using a combination of a wide range of complementary approaches, the proposed model for this epigenetic regulation. In the text it is clear that we used the result of ProYUC2:GUS together with many other arguments to characterize the action of APOLO on this target gene. We have not drawn our conclusions based on a single experiment.

Figure 1C- The description is missing in the manuscript.

R/ Thanks for this remark! We included the citation in the manuscript, line 170.

Suppl. Fig 10 please include statistics. Also, wherever mentioned in the manuscript, the term ‘t-test’ should be replaced with Student’s t-test (maybe author can read story behind use of pseudonym ‘Student’), unless otherwise stated which type of test was applied in analysis.

R/ Student’s t-test was indicated across the MS and we included stats in Figure S10.

In suppl. Fig 6A, the legend says VIM quantification of transcripts. Are authors showing VIM1 levels or YUCCA2 levels? Which one is correct? Based upon reviewers’ responses, it is assumed that transcript quantification is for YUCCA2. Please correct it. In addition, what is OE-VIM1-1? is this the overexpression of VIM1 in vim1-1 insertional mutant? Or this is X-axis labelling error?

R/ As indicated above each panel, several genes were analyzed in all the mutant and transgenic lines. The previous legend said “Transcript levels of VIM1 and APOLO potential common targets”, we did not mean VIM1 levels, we meant the levels of its targets. We have now rephrased the legend for clarity. OE-VIM1-1 is the 35S:GFP:VIM1 in the WT background, as indicated previously in the manuscript. We have now added a clarification in the legend as well.

The RT-qPCR primers for YUCCA2 are missing in provided table. Did authors use ChIP-qPCR primers for RT-qPCR as well?

R/ Thanks for pointing this out. Primers (taken from Cheng et al., 2006) were different and we have included the missing ones in the list.

L42-43, L135- please include a phrase, evolutionary unrelated lncRNA with potentially similar structures may exert similar functions

R/ Thanks for this suggestion, we have changed the text accordingly.

Please include information/figure about the nature of genes that are commonly affected in OE-APOLO and OE-UPAT.

R/ Thanks for this suggestion. Out of the 493 common DEG in OE:APOLO and OE:UPAT in control conditions, 263 (53%) are modulated by warmth in our RNA-Seq. This is now indicated in the text, and the list of genes is indicated in new Table S5b. Lines 443-444 and 466-467.

……………………………………………………………………………………………………

….

Reviewer #2: The authors have addressed most of my concerns and the manuscript has improved a lot.

R/ We thank the Reviewer for his/her positive remark. Still a few points remain:

Previous remark 3: I still miss the genetic analysis of vim2 and vim3 mutants to analyse their role in thermomorphogenesis.

R/ In the latest version of our work, we have included novel data showing that although weak, we could detect the interaction between VIM2 and APOLO in tobacco leaves.

The characterization of vim2 and vim3 mutants in response to warmth is out of the scope of this work, mainly focused VIM1-LHP1-APOLO complex regulating common target genes. As we mentioned in the text VIM1 behaves differently than VIM2 and 3 during the thermomorphogenic response and we have the phenotype with the single vim1 mutant further supporting the lack of redundancy on this specific response.

Nevertheless, we have now included a phrase in the Discussion proposing that further research will be needed to uncover if VIM2 and VIM3 are involved in thermomorphogenesis (line 513-514).

Previous remark 6: Genetic interaction between YUC2 and VIM1 and APOLO: the authors stated that a genetic interaction analyses is not possible because yuc2 has shorter hypocotyls. I do not agree here as effects in a double mutant may be additive or epistatic and 35S::YUC2 may rescue the vim1 phenotype.

R/ We think that our previous response was not clear enough. We include here your previous comment and our corresponding response:

Previous 6. While YUC2 appears to be regulated by APOLO and VIM1, its relevance for the phenotype is unclear. Currently, it is picked as one candidate, but genetic analyses would need to be provided.

R. It was previously shown that APOLO also regulates auxin transport (Ariel et al., 2020 Mol Cell), which may be related to the thermomorphogenic response. The observed phenotype in response to warmth may be due to alternative pathways controlled by APOLO, and we have picked YUC2 as a novel and interesting target for validation of action of the LHP1-VIM1-APOLO complex. We have now clarified that YUC2 is just one among several common targets (lines 240-243 and 251-256 in Results and 512-513 in Discussion) and we have analyzed the transcriptional behavior of a subset of them including YUC2 (Supplementary Figure 6A). Please note that yuc2 simple mutant is already impaired in hypocotyl elongation and that proYUC2:GUS plants served to reveal YUC2 promoter activation at warm temperatures.

New clarification/ We didn’t say that the cross is not possible. We meant that considering that yuc2 single mutant exhibits a hypocotyl elongation phenotype, then YUC2 is clearly related to this developmental context (together with the activity of the promoter) with no redundancy with other YUCCA genes. What we stated in our previous response and we still think is YUC2 is just one among other auxin-related targets of APOLO (pay attention to this phrase included in our previous response: "The observed phenotype in response to warmth may be due to alternative pathways controlled by APOLO, and we have picked YUC2 as a novel and interesting target for validation of action of the LHP1-VIM1-APOLO complex"). Thus, the phenotype linked to APOLO may be the result of a global deregulation of auxin homeostasis, including, among other features, the action of YUC2. Therefore, we do not propose that YUC2 is the only target mediating APOLO action, and we think that the suggested genetic study will not contribute to the main message of this article.

Generally thermomorphogenesis phenotypes: only the ratios of hypocotyl length at 23/29 °C are provided, also the data without the normalization should be provided for the experiments.

R/ Please note that in response to your previous request, we had already included all the separate measurements and their related statistics in Supplementary Table 1: Quantification of hypocotyl length of Arabidopsis over expression or mutant lines grown at 23 °C or 29 °C.
